# Optimising the use of caesarean section: a generic formative research protocol for implementation preparation

**DOI:** 10.1186/s12978-019-0827-1

**Published:** 2019-11-19

**Authors:** Meghan A. Bohren, Newton Opiyo, Carol Kingdon, Soo Downe, Ana Pilar Betrán

**Affiliations:** 1 Gender and Women’s Health Unit, Centre for Health Equity, University of Melbourne School of Population and Global Health, 207 Bouverie St, Carlton, VIC Australia; 2 UNDP/UNFPA/UNICEF/WHO/World Bank Special Programme of Research, Development and Research Training in Human Reproduction (HRP), Department of Reproductive Health and Research, World Health Organization, 20 Avenue Appia, Genève, Switzerland; 3 School of Community Health and Midwifery, Faculty of Health and Wellbeing, University of Central Lancashire, Preston, UK

**Keywords:** Caesarean section, Implementation science, Complex intervention, Childbirth, Formative research, Qualitative research, Behavioural change

## Abstract

**Background:**

Caesarean section rates are rising across all geographical regions. Very high rates for some groups of women co-occur with very low rates for others. Both extremes are associated with short and longer term harms. This is a major public health concern. Making the most effective use of caesarean section is a critical component of good quality, sustainable maternity care. In 2018, the World Health Organization published evidence-based recommendations on non-clinical interventions to reduce unnecessary caesarean section. The guideline identified critical research gaps and called for formative research to be conducted ahead of any interventional research to define locally relevant determinants of caesarean birth and factors that may affect implementation of multifaceted optimisation strategies. This generic formative research protocol is designed as a guide for contextual assessment and understanding for anyone planning to take action to optimise the use of caesarean section.

**Methods:**

This formative protocol has three main components: (1) document review; (2) readiness assessment; and (3) primary qualitative research with women, healthcare providers and administrators. The document review and readiness assessment include tools for local mapping of policies, protocols, practices and organisation of care to describe and assess the service context ahead of implementation. The qualitative research is organized according to twelve identified interventions that may optimise use of caesarean section. Each intervention is designed as a “module” and includes a description of the intervention, supporting evidence, theory of change, and in-depth interview/focus group discussion guides. All study instruments are included in this protocol.

**Discussion:**

This generic protocol is designed to underpin the formative stage of implementation research relating to optimal use of caesarean section. We encourage researchers, policy-makers and ministries of health to adapt and adopt this design to their context, and share their findings as a catalyst for rapid uptake of what works.

## Resumen

**Antecedentes:**

El uso de la cesárea está aumentando en todo el mundo. Tasas muy altas de cesárea en algunos grupos de mujeres coexisten con tasas muy bajas en otros grupos. Ambos extremos conllevan riesgos a corto y a largo plazo y constituyen un importante problema de salud pública. Un uso adecuado de la cesárea es crucial para proveer una atención clínica sostenible de calidad durante el embarazo y el parto. En 2018 la Organización Mundial de la Salud publicó recomendaciones de base fáctica para reducir las cesáreas innecesarias a través de intervenciones no clínicas. Dicha guía detecta importantes lagunas en la investigación e insta a que se realice una investigación formativa previa a la implementación de estrategias destinadas a reducir u optimizar el uso de cesáreas, investigación que tiene como objetivo identificar los determinantes del uso de la cesárea y otros factores que pueden afectar la implementación de dichas estrategias a nivel local. El presente artículo ofrece un protocolo genérico de investigación formativa que puede ser utilizado como guía por todos aquellos que quieran planificar una intervención para optimizar el uso de la cesárea. Tiene como objetivo evaluar y entender el contexto en el que se piensa actuar.

**Métodos:**

Este protocolo de investigación formativa tiene tres componentes: (1) examen de la documentación; (2) evaluación de la factibilidad; y (3) investigación cualitativa con las mujeres, los proveedores de servicios de salud y la administración de los centros. Los dos primeros componentes incluyen herramientas para llevar a cabo un mapeo local de las políticas de salud, las prácticas y los protocolos clínicos, y la organización de la atención que se ofrece, lo que permite describir y evaluar el contexto local antes de empezar a actuar. La investigación cualitativa está organizada en torno a 12 intervenciones que podrían reducir las cesáreas innecesarias. Cada una de ellas se presenta en un «módulo» que contiene la descripción de la intervención y las pruebas que la refrendan, la teoría del cambio y la planilla para las entrevistas o los grupos de debate. Todos los instrumentos necesarios están incluidos en el protocolo.

**Discusión:**

Este protocolo genérico esta diseñado para llevar a cabo una investigación formativa previa a la realización de intervenciones destinadas a optimizar el uso de la cesárea a nivel local. Investigadores, responsables de la formulación de políticas y ministerios de salud en todo el mundo pueden adoptar y adaptar el protocolo para usarlo en su contexto particular. Alentamos a que se compartan los resultados obtenidos a fin de catalizar la investigación y promover el acceso a intervenciones efectivas.

## Abstrait

**Contexte:**

Les taux de césariennes sont en hausse dans toutes les régions du monde. Des taux très élevés dans certains groupes de femmes coexistent avec des taux très bas dans d’autres. Ces deux extrêmes sont associés à des conséquences négatives à court et à long terme. Ceci constitue un problème de santé publique majeur. Optimiser l’utilisation de la césarienne est crucial pour des soins de santé maternelle durables et de bonne qualité. En 2018, sur la base des recherches réalisées, l’Organisation Mondiale de la Santé a publié des recommandations d’interventions non-cliniques pour la réduction des césariennes non nécessaires. Ce guide a identifié des lacunes notables en matière de recherche et a appelé à la réalisation de recherches formatives avant la réalisation d’une intervention, afin de définir les déterminants locaux des naissances par césarienne et les facteurs qui pourraient affecter la mise en œuvre de stratégies d’optimisation multidimensionnelles. Ce protocole de recherche formative original a été créé comme un guide pour l’évaluation et la compréhension du contexte à l’attention de ceux qui prévoient d’agir pour optimiser l’utilisation de la césarienne.

**Méthodes:**

Ce protocole de recherche formative inclut trois composantes principales: (1) une revue de la littérature; (2) une évaluation rapide de l’état de préparation; (3) une recherche qualitative initiale avec les femmes, les prestataires de soins de santé et les administratifs. La revue de la littérature et l’évaluation rapide de l’état de préparation incluent des outils pour la cartographie de la mise en œuvre de politiques locales, de protocoles, de pratiques et d’organisation des soins afin de décrire et d’évaluer le contexte avant toute mise en œuvre. La recherche qualitative est organisée en 12 interventions susceptibles de réduire les césariennes non nécessaires. Chaque intervention est conçue comme un « module » et comprend une description de l’intervention, des éléments de revue de la littérature, une théorie du changement, ainsi que des guides pour des entretiens approfondis et des discussions de groupe. Tous les outils d’études sont inclus dans ce protocole.

**Discussion:**

Ce protocole original a été conçu pour venir en appui d’une recherche formative qui constitue une étape d’une recherche de mise en œuvre visant à une utilisation optimale de la césarienne. Nous encourageons les chercheurs, les responsables de politiques et les ministres de la santé à adopter et adapter ce protocole à leur contexte, ainsi qu’à partager et utiliser leurs résultats comme un catalyseur pour l’adoption rapide de mesures efficaces.

## Plain English summary

Many women across the world give birth by caesarean section, which can be a life-saving intervention for both the woman and her baby. However, some women have a caesarean section even if there is not a medical need to have one. This can lead to short- and long-term risks for the woman and her baby. We use the term “optimising the use of caesarean section” to refer to making the best possible use of caesarean section to improve the health and well-being of women and their babies.

There are many factors contributing to high caesarean section rates including incentives for healthcare providers, a culture of intervention in the hospital setting, healthcare providers’ fear of blame, beliefs about provider-patient relationships, perceptions about the convenience about different types of birth, and social norms around birth. These multiple factors need to be addressed when designing research, evaluation or health services to optimise the use of caesarean section.

In this paper, we propose a research protocol that can be used by researchers, ministries of health, or others to understand and address caesarean section. We propose to start with formative research. Formative research refers to gathering data that might be useful to understand the issue and people the issue is affecting, then use this data to develop programs or further research. We provide a template for how this research could be conducted, including the research protocol (background, rationale, objectives, design, methods and logistics) and study tools (a document review, readiness assessment, and qualitative research guides).

## Background

Caesarean section is a surgical procedure that can prevent maternal and newborn mortality when used for medically indicated reasons [1]. However, there is no evidence of benefits for women or babies who do not have a medical indication [2]. As with all surgical procedures, caesarean section is associated with short and long-term risks for women, children, and future pregnancies, as well as substantial healthcare costs [2–5]. These risks are higher in settings where women have limited access to comprehensive obstetric and post-surgical care. Rapid rises in caesarean section without concurrent decreases in maternal or perinatal morbidity or mortality suggest that a large proportion of caesarean sections are unnecessary. Across 150 countries, 18.6% of all births are estimated to occur by caesarean section, ranging from 1.4 to 56.4% across different countries [6]. The global caesarean section rate increased by 12.4% (from 6.7 to 19.1%) from 1990 to 2014 [6].

Rising caesarean section rates are a global challenge that affects high-, middle-, and low-income countries across all geographical regions [1, 6, 7]. The causes of increased rates vary across and within contexts, and may include differences in professional practices, fear of medical litigation and associated professional risk-aversion, changes to the characteristics of the population (e.g.: increasing prevalence of obesity, or increasing proportion of older women or multiple births), as well as economic, organisational, and sociocultural factors such as generational shifts in work and family responsibilities, women’s increasing desire to determine how and when their babies are born, and physician preferences [1, 8–10]. Sustained increases in caesarean section rates are a major public health concern and there is an urgent need for evidence-based guidance to address this trend [1]. Optimising caesarean section rates (i.e. making the best or most effective use of caesarean section to improve the health and well-being of women and their babies) is also a critical component of improving the quality of care during childbirth [11]. This includes ensuring the availability, accessibility and affordability of caesarean section when needed by a woman and/or her baby. However women give birth, providers should ensure that care is provided with respect, maintaining dignity, and that women understand and consent to both what is happening in the moment and to what may happen in the future [11].

In 2018, the World Health Organization (WHO) published evidence-based recommendations on non-clinical interventions to reduce unnecessary caesarean section [1]. Evidence for the effectiveness of interventions was derived from an updated Cochrane review of 29 studies [12]. This was complemented by an analysis of values, acceptability, equity, resource implications, and feasibility of the included interventions, derived from three qualitative evidence syntheses [13–15]. The guideline contains five recommendations on non-clinical interventions to reduce unnecessary caesarean section (Table 1), which are designed to inform the development of national and subnational policies and protocols in this area, and should be implemented in conjunction with other interventions to improve the quality of care during childbirth [1].

**Table 1:** The three groups of interventions included in the WHO recommendations: non-clinical interventions to reduce unnecessary caesarean section [1]. Interventions are grouped according to their target population: women, healthcare professionals, and health organizations, facilities or systems

A. Interventions targeted at women 1.0 Health education for women, including childbirth training workshops, nurse-led applied relaxation training programmes, psychosocial couple-based prevention programmes, and psychoeducation (*context-specific recommendation, only with targeted monitoring and evaluation*). B. Interventions targeted at healthcare professionals 2.1 Implementation of evidence-based clinical practice guidelines, combined with structured, mandatory second opinion for caesarean section indication (*context-specific recommendation, only in settings with adequate resources and senior clinicians able to provide mandatory second opinion*) 2.2 Implementation of evidence-based clinical practice guidelines, caesarean section audits and timely feedback to healthcare professionals (*recommended*) C. Interventions targeted at health organisations, facilities or systems 3.1 Collaborative midwifery-obstetrician model of care (e.g.: a model of staffing based on care provided primarily by midwives, with a 24-h obstetrician back up who provides in-house labour and delivery coverage without other competing clinical duties) (*context-specific recommendation, only in the context of rigorous research*) 3.2 Financial strategies (e.g. insurance reforms equalising physician fees for vaginal births and caesarean sections) for healthcare professionals or healthcare organizations (*context-specific recommendation, only in the context of rigorous research*)	

The WHO guideline highlighted key research gaps around uncertainty in the effects of interventions, applicability of evidence to other settings, and limited evidence contributing to the guideline questions [1]. In line with implementation science principles for complex interventions, the WHO guideline also highlighted that future intervention and implementation research in this area should be preceded by formative research to define locally relevant determinants of caesarean birth and potential interventions [1]. Given the complex factors contributing to rising caesarean section rates, implementation research is a useful approach to engage key stakeholders across multiple disciplines to better understand and design interventions. Using an implementation research approach is particularly useful for changing organisational structures and individual behaviours around practices that may be resistant to change, including sub-optimal use of caesarean section.

In this paper, WHO is proposing a generic formative research protocol that can be adapted and implemented in different contexts to guide the design and implementation of interventions to reduce unnecessary caesarean section rates. This protocol is designed as a generic protocol for the formative stage in preparation for the implementation of targeted interventions and/or trials, and is expected to be adapted and adopted by different sites (by WHO, ministries of health, or other research partners). The protocol provides guidance and a range of tools to assist teams in this endeavour, and can be tailored for what works in different settings. Local findings based on this protocol could be used to design implementation studies and to frame implementation strategies, including formal baseline and endline assessment of the effectiveness of the implementation process as a whole. Theoretically, this formative research would represent the first phase in a multi-phase project, using different methods and approaches:

- Phase 1: formative phase consisting of a document review, readiness assessment and primary qualitative research
- Phase 2: intervention design and preparation of implementation strategy
- Phase 3: intervention implementation and evaluation, including baseline and endline assessment

The objective of the overall project would be to design and implement a multifaceted strategy that is locally relevant, culturally accepted by women and providers and can be implemented effectively to reduce unnecessary caesarean sections. This protocol outlines Phase 1 of this project *only* (formative phase), to inform the development of interventions.

### Conceptual framework

**Fig. 1:**
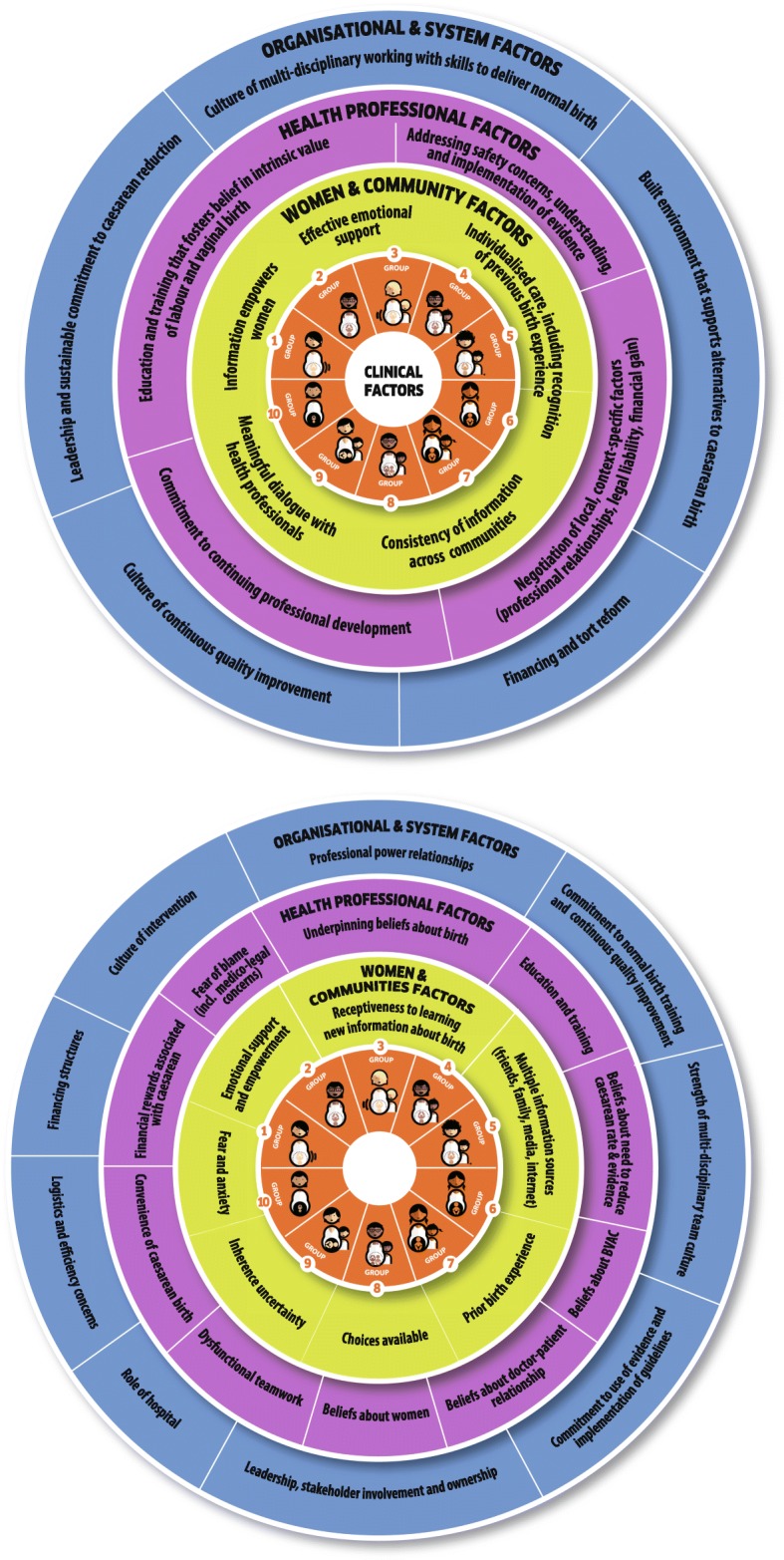
Ecological model to understand factors influencing caesarean section rates related to women, society, health providers, and healthcare organizations that affect caesarean section use at the local level. These factors surround the obstetric and clinical factors that also affect the frequency of births by caesarean section, which are represented in the middle by the Robson 10-group classification (reproduced with permission from [16])

The WHO guideline proposes a new ecological framework for understanding the different levels of factors affecting caesarean section rates (Fig. 1) [1]. This includes influences from clinical factors, women and their families, communities, health professionals and larger organisational and systems factors [1]. Women receive information about pregnancy and childbirth from multiple informal and formal information sources, including their friends, families, media and internet. This information can shape their opinions and choices about their preferred mode of childbirth, where to give birth, and how to take care of their babies [17, 18]. Women’s networks can provide them with emotional support and empowerment, and can influence their levels of fear, anxiety and uncertainty. Likewise, women’s own previous birth experiences may influence her choices and preferences for subsequent pregnancies. Discussing a woman’s previous birth experiences with a healthcare provider may help to provide more individualised care for a woman and ensure that she has a meaningful dialogue with her care team. These influences and experiences ultimately shape women’s preferences for her preferred mode of childbirth [13].

Health professionals working on maternity wards also shape the context of women’s mode of childbirth. Providers have sets of beliefs developed from their own training and experiences regarding the intrinsic value of different modes of birth and childbirth practices [13–15]. Likewise, their training and education shapes their skillset for managing different modes of birth, their opinions regarding the necessity of reducing caesarean section rates, and their opinions and skills for managing vaginal birth after caesarean section. Providers work within the structures of care and teams within the health facility, which may impact the perceived convenience and financial rewards of caesarean birth. They must navigate decisions about patient care within the context of locally-relevant factors such as professional relationships, legal liability, and financial gain.

Organisational culture also influences caesarean section. A culture of continuous quality improvement may promote the identification of potential issues and promote action to resolve issues, such as high or increasing caesarean section rates. Committing to a practice of evidence-based childbirth care and local implementation of guidelines and protocols may influence the use of caesarean section. Leadership and promoting team-based care can improve skills to manage vaginal birth and provide a supportive learning environment. The built environment of the facility, such as the available bed space and structure of the labour ward, plays an important role in the organisation of care. A culture of medicalisation of childbirth may lead to higher than necessary rates of caesarean section, among other unnecessary interventions. Lastly, legal liability for the health outcomes of women and babies may influence the level of intervention or risk that providers are comfortable with [14, 15].

Interventions to reduce unnecessary caesarean section should consider these multi-level influences on the decision-making process for mode of birth. Given the multiple levels of influences, this protocol includes formative research about a package of interventions that addresses different factors contributing to unnecessary caesarean section. This protocol is designed to help local teams to select intervention(s) that are most likely to work in their settings and provide critical information on how to implement them.

### Objectives

1. To explore how national, sub-national and facility-level policies and practices influence the feasibility, availability, and implementability of interventions to reduce unnecessary caesarean section;
2. To explore the readiness of health facilities to implement interventions to reduce unnecessary caesarean sections;
3. To explore how different interventions to reduce unnecessary caesarean section should be implemented in a specific context;
4. To explore implementation considerations, including expectations, preferences, feasibility and acceptability of different interventions to reduce unnecessary caesarean section, from the perspectives of the following stakeholders:
  - Women (nulliparous, and multiparous with and without previous caesarean section)
  - Providers (midwives, nurses, doctors, administrators working on the maternity ward)
  - Policy-makers; and
5. To assess potential facilitators and barriers to the implementation of different interventions to reduce unnecessary caesarean section in a specific context.

## Methods

### Project description

**Fig. 2:**
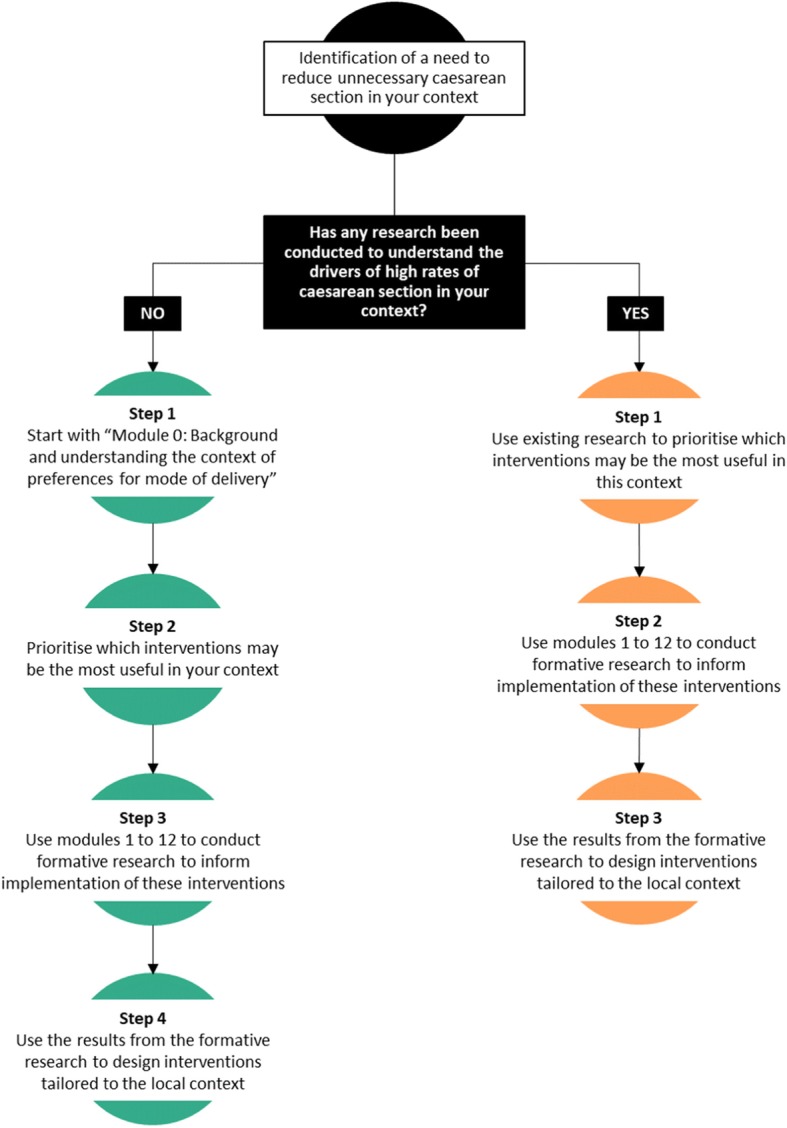
Flow chart to determine where to begin reducing unnecessary caesarean section in your context

This study protocol outlines the formative phase activities to inform the development of interventions to reduce unnecessary caesarean section. Table 2 provides an overview of how to use this protocol, and Fig. 2 depicts a flow chart to determine where to begin reducing unnecessary caesarean section in a specific context. The formative phase of this project has three main components: (1) document review; (2) readiness assessment; and (3) primary qualitative research. Detailed overviews of each component are described in the following sections.

**Table 2:** How to use this protocol. This table provides an overview of how to use this protocol

How can you use this guide? There are complex and multiple factors contributing to rising caesarean section rates, and these factors may vary widely between countries. Prior to implementing any interventions to reduce caesarean section rates, research should be conducted to understand women’s and providers’ views on why rates are increasing in a particular setting. To inform this process, WHO proposes this template for formative research that can be adapted and implemented in different contexts. Conducting this formative research will help you to identify the local reasons for increasing caesarean section rates and how to design and develop locally feasible and acceptable interventions to reduce rates. Who can use this guide? The primary audience for this guideline and research protocol template are health professionals responsible for developing regional, national, and local health protocols and policies, as well as midwives, obstetricians, nurses, medical practitioners, healthcare managers and policymakers in all settings and countries [1]. Any countries, research teams or program developers who are interested and committed to reduce the use of unnecessary caesarean section in their context are welcome to use this protocol to conduct their own research. What is included in this guide? This formative research project consists of three main research activities: (1) document review; (2) readiness assessment; and (3) primary qualitative research with women, healthcare providers, and healthcare administrators. The document review and readiness assessment will help the research teams to identify important barriers and enablers about policies, protocols, practices and organization of care to describe and assess the service context ahead of implementation. The qualitative research is organized according to interventions that may reduce unnecessary caesarean section, and will help to develop key implementation considerations for each intervention. Twelve potential interventions have been identified through the guideline development process (see Additional file 1). It is envisioned that the research team and key stakeholders will select one or more interventions for potential implementation in their context, based on a prioritisation exercise. Each intervention is described in detail in a “module” that outlines the following: 1. Background and overview of the intervention 2. Supporting evidence 3. Theory of change 4. Guiding principles 5. Interview/focus group discussion guide for each participant group The WHO guideline recommends that multifaceted (rather than single-component) interventions are used to reduce unnecessary caesarean section. If two or more interventions are prioritised, then it is envisioned that data collection efforts can be combined for the participant groups. For example, if both interventions include a component for in-depth interviews with healthcare providers, then it would be reasonable to combine the discussion guide questions into one interview to improve efficiency. Where do I begin? Given the current state of research evidence about caesarean section rates and drivers, we hypothesize that there are two scenarios in which research teams would use this protocol (Fig. 2). Once the research team has identified which scenario they fit into, and prioritised which interventions to implement, then local ethics approval should be sought.

Twelve potential interventions that may influence use of caesarean section were identified through the guideline development process (see Additional file 1 for a description of each intervention, theory of change, and supporting evidence). The document review (Additional file 2), readiness assessment (Additional file 3) and qualitative research focus on the potential for implementation of these interventions, as well as a background module to understand the context of preferences for mode of birth (Additional file 4). The twelve interventions are:

1. Prenatal education and support (Additional file 5)
2. Decision-aids for mode of birth (Additional file 6)
3. Psychosocial support for women with a fear of childbirth (Additional file 7)
4. Labour companionship (Additional file 8)
5. Public dissemination of caesarean rates at a facility-level (Additional file 9)
6. Audit and feedback (including Robson classification and external review of labour and delivery records) (Additional file 10)
7. Mandatory second opinion for caesarean birth (Additional file 11)
8. In-service training and implementation of clinical protocols (Additional file 12)
9. Equalizing physician pay for vaginal and caesarean birth (Additional file 13)
10. Opinion leader education (Additional file 14)
11. Setting a goal for caesarean section rate at a facility-level (Additional file 15)
12. Policies limiting legal liability and malpractice lawsuits (Additional file 16)

### Study sites

This study will be conducted in a set of health facilities where interventions to reduce unnecessary caesarean section are planned to be implemented. This protocol is designed to be applied to both public and private health facility contexts. The recruitment of participants (particularly women) may need to be modified if this protocol is implemented in contexts with a large private health sector, as it is possible that women may attend public facilities for antenatal care (where recruitment would take place) but give birth in a private facility. These considerations should be discussed with the country research team prior to implementation and addressed in site specific protocols. Some changes may be required to ensure the protocol is relevant for specific sites.

### Document review

Using a structured approach to identify important barriers and enablers to implementing interventions to reduce unnecessary caesarean section will help to ensure that important barriers are not overlooked, and that important enablers are considered in the implementation plan [19]. These barriers and enablers may be national, regional, or contextually-specific, and therefore dependent on local circumstances to inform judgments about what decisions and actions to take for implementation [20]. For the purposes of this study, “local” refers to facility, district, subnational and national levels, and the goal is to identify important factors for implementation considerations [20]. For example, this could include national guidelines, professional policies, professional responsibilities (which cadre of providers make decisions about interventions), capacity of the health facilities and system, the availability of providers and equipment, political traditions or other cultural norms, costs of implementation, and the characteristics of the implementation area and of those who reside there [20]. This information is useful to assess the applicability and use of global reviews of effectiveness to the local context [12]. Furthermore, this information can inform assessments of the impact of the intervention, inform judgments about the likely values and preferences of users and providers, determine what resources are available and what might need to be sourced, and suggest how the sustainability of the intervention(s) may be maintained after the end of the formal implementation project [20]. Local information to address these topics may be obtained from several different sources, such as reviewing policy documents and existing research on caesarean section conducted in a similar context [13, 14], as well as through qualitative research with key stakeholders (explained in detail in this protocol).

For this study, the starting point for exploring the local context is through a document review of the relevant resources to answer key questions about the context of maternity care and caesarean section (full document review form located in Additional file 2). The document review should be conducted systematically in order to ensure that important data are not omitted or overlooked. The protocol follows the principles of evidence-informed policy-making outlined in the SUPPORT tools to ensure that information from the local context is adequately considered [20].

### Readiness assessment

After the document review, the local team will be better informed to conduct the readiness assessment. The readiness assessment will allow the team to describe and assess the service delivery context ahead of implementation, and may be carried out concurrently with the qualitative research in each study site. Providing a systematic approach to assessing readiness will ensure that the local situation informs and tailors the intervention(s) in a way that is suitable for implementation in that context. Responses to the domains in the readiness assessment will be combined with the findings from the qualitative research to identify and prioritise barriers and to develop potential multifaceted interventions and considerations for implementation. The readiness assessment template is available in Additional file 3.

### Qualitative research

To meet the study objectives, qualitative research methods will be used, specifically in-depth interviews (IDIs) and focus group discussions (FGDs) among different cadres of stakeholders, including (at a minimum) women, healthcare providers, and administrators. It may be appropriate to include other key stakeholders, such as partners, husbands, family members, community influencers or service funders, depending on the local drivers for caesarean section rates. The appropriate mix of stakeholders will be identified by the local research team in consultation with clinical staff and community members. The purpose of these IDIs and FGDs is to explore implementation considerations for interventions to reduce unnecessary caesarean section (expectations, preferences, feasibility, and acceptability), facilitators and barriers to successful implementation, and what the components of the intervention should look like in a specific context.

#### Participants and recruitment

The following participants are proposed:

1. ***Maternity service users***, including:
  - Pregnant nulliparous women
  - Pregnant multiparous women with a previous caesarean section
  - Pregnant multiparous women without a previous caesarean section
2. ***Maternity service providers***, including:
  - Obstetricians and other doctors working on the maternity ward, including trainees, medical officers
  - Midwives and/or nurses
  - Other skilled birth attendants as appropriate
3. ***Facility administrators***, including:
  - Matron-in-charge of the labour ward
  - Head of obstetrics
  - Medical director
  - Other administrators such as head of finance, legal director.

In each site, we suggest that all three main groups of participants are included, though specific sub-groups may vary depending on who is most likely to influence the use of caesarean section locally. For example, in some contexts it might be appropriate to include other groups of maternity service users such as partners/husbands, other family members, or community leaders.

Pregnant women aged 18 to 49 years who attend antenatal care will be invited to participate in FGDs. If appropriate in a given context, pregnant adolescents (e.g. aged 15 to 17 years) may also be included, for example in settings where adolescent pregnancy rates are high, or where pregnant minors are considered emancipated. The local research team will ensure that a diverse group of women are included, including a mix of urban/rural, parity, age, ethnicity and religion, in order to account for the views of multiple end users. The research teams will facilitate contact with women during their visit to the health facility for antenatal care. In the appropriate area of the health facility (e.g.: antenatal care waiting area and patient rooms) informational materials (in appropriate local languages) about the study will be displayed, such as posters and pamphlets using visual information to ensure accessibility of information. The informational materials will contain information about the study, eligible participants, and how to participate. To help facilitate recruitment and participation, at least one researcher will be on site, and they will not be a part of the staff taking care of the patient (and ideally, not a clinician). This will help to ensure that consent to participate is not influenced by power imbalances.

We have defined ***providers*** as doctors, nurses, midwives, and other skilled birth attendants working on the maternity ward in the study facilities. These providers will be invited to participate in IDIs. The research team will ensure that, to the extent possible given health workforce constraints, a diverse group of providers are interviewed in each facility, including by age, gender, and years of experience. We have defined ***administrators*** as those working as managers on the maternity ward or health facility (e.g.: medical/clinical director, head of obstetrics, matron-in-charge, and finance/legal officers). These administrators will be invited to participate in IDIs. Given the small number of people in these leadership positions, we do not expect that it will be possible to stratify by additional sociodemographic characteristics. Providers and administrators will be contacted at their place of work in the study hospital. In the appropriate area of the health facility (e.g.: staff break room or resting area), informational materials about the study will be displayed, such as posters and pamphlets. The informational materials will contain information about the study, eligible participants, and how to participate. Potentially eligible participants who are providers or administrators may also be identified from staff records, then contacted on an individual basis to participate (e.g.: via email or telephone).

Each individual will be provided with information about the study and invited to participate. If they agree to participate, they will be asked to provide consent. All IDIs and FGDs will take place in a private setting and will be audio recorded. IDIs are anticipated to last from 20 to 60 min (depending on the number of modules/interventions included), and FGDs are anticipated to last approximately 60 min (although this may be longer in some contexts). IDIs and FGDs will be facilitated by the research teams. For the FGDs conducted with women, the research assistants will be female.

#### Sampling

Maximum variation sampling will be used to achieve a diverse sample of participants, to make sure that the findings are a reasonable reflection of the views and experiences of local stakeholders. This method uses pre-specified parameters to stratify the sample [21] and encourages recruitment and sampling based on diversity. Table 3 outlines the sampling grid with the stratification proposed for conducting IDIs with providers and service users, for each facility included in the study. In each of the study facilities, healthcare providers will be sampled based on their cadre, such as nurse/midwives or doctors/specialists. In each facility, facility administrators will be selected based on the managerial organization of the facility. We expect the type or designation of facility administrators to vary by facility, but at the minimum would include the medical administrative head of the facility, the head of the obstetrics and gynaecology department, and relevant administrative staff responsible for financial and legal matters.

**Table 3:** Sampling grid per study facility

	n (per study facility)
Providers	3–6 IDIs
Nurses/midwives	1–2 IDIs
Doctors	1–2 IDIs
Administrators	1–2 IDIs
Users	3–6 FGDs
Pregnant nulliparous women	1–2 FGDs
Pregnant multiparous women with a previous caesarean section	1–2 FGDs
Pregnant multiparous women without a previous caesarean section	1–2 FGDs

Depending on the scale of implementation, IDIs with users (women) may either be conducted in all of the study facilities, or in a subset of the study facilities. For example, if ten facilities are included in this study, then it may be more feasible to conduct IDIs with users in four or five facilities, provided that there is not expected to be substantial variation in the characteristics of users (for example, if two or more facilities are located in the same city). This subset of facilities should be chosen to ensure diversity between facilities, such as by geographical region, urban/rural location, or level of care (e.g.: secondary/tertiary or district/state levels). Efforts will be taken to have a diverse sample of users, including nulliparous and multiparous women, older and younger women, and women of different religions or ethnicities.

The proposed sampling grids (Table 3) will guide the data collection process; however it is advisable for data collection to continue until no new insights emerge (the point of data saturation). If this point is not reached after data collection with the pre-specified number of participants, more participants should be recruited until the team agrees that saturation has been reached. Sampling may also need to be adjusted if minority groups (e.g. based on religion, ethnicity, migration status, Indigeneity, etc) are specified as key populations. These adjustments will be made on a site-specific basis. Once the IDIs and FGDs are conducted, the study participants will not be followed up on an individual basis, unless member-checking occurs.

#### Follow-up procedures

After the completion of data collection, transcription and translation (if necessary), a data analysis workshop will be held in order to facilitate analysis and interpretation of findings at a local level. This workshop should be facilitated by the research teams and include key stakeholders such as hospital staff, community members, and women's groups. The workshops will assist in refining which interventions and approaches may work best and how they could be put into practice. The emerging findings from the providers and administrators will be fed back to and discussed with the facility teams during the data analysis process to improve trustworthiness of the interpretation. The findings will be discussed with different stakeholders and these findings will be used to inform the development of the implementation research intervention. The research findings will also be communicated back to the community members, for example through presentations, group meetings, posters in the facility, or (when available) through mobile technologies and social media..

#### Study instruments

The instruments include a document review form, readiness assessment form, and semi-structured interview guides. Eligible individuals will also complete a consent form prior to participation. The instruments are available in Additional files 2, 3, 4, 5, 6, 7, 8, 9, 10, 11, 12, 13, 14, 15 and 16.

#### Data management and quality assurance

Prior to data collection, a training session will be conducted for all the research teams. We suggest that this training workshop is up to 3-5 days in duration to cover the background of the project, research design, manual of operations, review and pilot the study tools, ethical considerations, informed consent, and project implementation plan. Depending on the composition of the research team, this may include country PIs, social scientists, research coordinators, research assistants, transcribers/translators and any other relevant team members. This training session will ideally build the research capacity in each site, guide all involved in the study in to the objectives of the study, ethical and governance considerations and data collection procedures. It will cover practice sessions with the tools, as well with undertaking recruitment and consent procedures appropriately.

During the data collection period, principal investigators will be in constant communication with the research assistants in the field in order to respond to any issues that arise during data collection. Ideally, transcription will occur in parallel to data collection and will be shared on an on-going basis with the study team to ensure the quality of the data and to determine if certain themes need to be further explored. This may include providing feedback on topics that could be probed more deeply during future interviews, identification of areas for improvement, and facilitating dialogue with the country teams regarding saturation of data.

All digitally recorded qualitative data will first be transcribed verbatim in the original language used for collection using a structured format. Verbatim transcription will be performed close to the time of completion of the interviews to maintain the originality of the interview without loss of themes. Ideally, the person transcribing the data will be the same person who conducted the interview to improve trustworthiness of the data. If translation is needed, the research team will collectively decide on the most appropriate time to translate, which will depend on the study context, considering the following options:

- **Translate all study transcripts into a mutually intelligible language prior to analysis**: in situations where the research may have been conducted in dialects or local languages that are not fully understood by all members of the research team.
- **Conduct analyses in local or contextually-relevant language and translate final themes and key quotations for dissemination**: in situations where the research team fully understands the language(s) used for data collection, in order to help preserve the linguistic nuances.

Back translation should be conducted on a subset of transcripts or analytic units in order to validate the translations. Observations and assessments during interviews will be written up as field notes. The transcripts will be complemented with notes taken during the interviews. Data transcription will be performed under the supervision of the designated social scientist who will review all transcripts for completeness.

#### Data analysis

It is recommended that the qualitative data are analysed and interpreted using a thematic analysis approach [22]. A thematic analysis approach was chosen to identify, analyse and report patterns and themes within the data [22]. The thematic analysis will be performed according to the following steps: (1) organizing the data; (2) generating categories, themes, and patterns; (3) testing emergent hypothesis; and (4) searching for alternative explanations. The research team will conduct a thematic analysis to explore findings related to the objectives of the study. The team will explore common themes that span geographic and cultural differences while noting important differences across settings that need to be accounted for during the implementation phase. Throughout data collection, data analysis will take place in parallel to the point of data saturation. This close collaboration between the qualitative research team, the lead social scientists, and the data collectors will ensure quality analysis and interpretation of the data across sites and has proven to be an efficient process in previous studies coordinated by WHO [23–29].

#### Researcher reflexivity

Throughout the study design, analysis and interpretation process, the research team should reflect and discuss how their own training, life experiences, and perspectives may influence the analysis process (reflexivity). Considering reflexivity of the research team is a key component of conducting qualitative research, and should be reflected on throughout the project on an ongoing basis (including research design, selection of co-investigators, data collection training, analysis, interpretation and write-up). Given the expected multi-disciplinary nature of the research team (which may include, for example, obstetricians, social scientists, and maternal health researchers), we expect that the process of reflexive dialogue will yield enriching discussions about how to best design the interventions to reduce unnecessary caesarean section in a manner that accounts for the preferences and needs of women, healthcare providers, and healthcare administrators.

### Research team composition

In all cases, teams should involve project staff who are skilled at both quantitative and qualitative data collection, analysis, and interpretation, and stakeholders who can take the standpoint of local service users, community influencers. To ensure feasibility, reliability, and validity of the project, we suggest a diverse research team composition consisting of the following individuals. A principal/lead investigator whose responsibility is overall coordination and leadership. A lead social scientist with experience in qualitative data collection and analysis to coordinate the qualitative component and understand social science implications of findings. Facility-level research coordinators at each study site to act as the gate-keeper for the facility-based data collection (for example, this may be an obstetrics trainee, medical officer, or midwife/nurse). Research assistants to conduct the qualitative interviews (ideally female research assistants with a non-clinical background for data collection with women, and research assistants with some clinical knowledge or experience for data collection with providers). The team may also need transcribers and translators for the qualitative component, or research assistants may also be able to do these tasks.

## Discussion

### Gender, social equity and rights

All women have the right to high quality, respectful care during childbirth, and healthcare services need to be structured and organized in a fashion that helps protect and promote these rights. Overuse of interventions that are not needed undermines these rights, as does underuse of required interventions. This protocol is offered to those who aim to understand and improve how to implement interventions to reduce unnecessary caesarean section. Feminist and gender theories on human reproduction have historically struggled to understand and explain why some women actively pursue medical interventions during childbirth, including caesarean section. Contemporary feminist engagement with the medicalization of childbirth may view women who seek caesarean sections as relinquishing control to medical professionals, or consider why some women feel positive and empowered by relinquishing control [30, 31]. For example, a study conducted in India found that young women may seek caesarean birth as a means of gaining control over their bodies during the postnatal period, as their in-laws allowed them to have a longer recovery period after the birth [32]. In other settings, such as Brazil, women may view caesarean birth as the norm, and place value on caesarean birth as the highest quality of care attainable to them [33]. In Taiwan, women have conceptualised caesarean birth as a means to avoid “suffering twice” from the pain of labour and childbirth, and potential complications of vaginal birth on future sexual pleasure [34]. Therefore, some women may seek caesarean birth as a means to manipulate societal structures of power and gender inequality [33].

Public health research has demonstrated that women’s preferences for and knowledge of caesarean section is highly influenced by their sociocultural contexts, including the perspectives of their peers, families, religious communities, and the healthcare systems that they interact with [35, 36]. These entities can shape the way that women feel about their own bodies before, during and after pregnancy, such as the expectation of “returning to normal” after birth. This suggests that some women’s preferences for caesarean birth may be influenced by implicit socialisation by dominant values [34]. Feminist arguments emphasize the need to understand these influences and values, but also allow space for women to make choices over their bodies. A key challenge is ensuring that women can make informed choices based on adequate and accurate information of the benefits and risks of caesarean section, and to understand how these choices can be influenced by hospital or provider interests that may be rooted in patriarchal structures of medicine [37].

Research has demonstrated the existence of substantial within country economic inequalities in caesarean section across 72 low- and middle-income countries, where caesarean section rates were lowest in the poorest wealth quintile (median 3.7%) and highest in the richest wealth quintile (median 18.4%) [7]. Boatin and colleagues hypothesized that these inequalities may be due to inadequate access in emergency obstetric care among the poorest groups, and higher use of caesarean section without medical indication among the richest groups [7]. The protocol we offer in this paper will contribute to addressing these inequalities by providing foundational evidence on how to best design and implement interventions to reduce unnecessary caesarean sections, from the perspective of all relevant stakeholders, including pregnant and postnatal women, and junior front-line staff. Many of the proposed interventions have the potential to reduce inequalities, and promote health equity, although this has not yet been explored in the literature. For example, audit and feedback cycles may help to ensure that all women who have a medical indication for caesarean section receive one. Likewise, group therapy and decision aids for women may be particularly useful to women with a fear of childbirth or women with a previous caesarean section.

For each context that this project is implemented in, the intention is for the research team to consider how to include participants with different backgrounds and experiences. For example, when identifying groups of women to participate in the qualitative research, care should be taken to include women from different ethnic/racial groups, religions, and geographical residence. This will help to ensure that diverse perspectives are included when developing future interventions, which may help to reduce any existing inequalities in use of or access to caesarean section. Broad participation criteria should also ensure the inclusion, as far as possible, of all cadres of healthcare providers and women with different life situations (including religion, sexual orientation, socioeconomic status, ethnicity, age). Sub-groups of healthcare providers or women could include adolescents, unmarried women, women of different ethnicities, migrant women, women who are HIV positive, and junior staff who are not in positions of power. We consider it important to ensure the selection of participants does not discriminate against any group, as women in this category may be at greater risk of receiving poor quality care in the facility. If such women are included, they will be protected by the universal standards of confidentiality and privacy that apply to all participants. However, all women, including these vulnerable groups, should be free to refuse to participate, both confidentially and without prejudice.

### Ethical considerations

All potential participants will receive information about the study in their language of choice, conforming to ethical requirements for research involving human subjects. The language should be easy to understand and free of technical jargon. Participants will be given sufficient time to reflect on the information and ask questions. Those who consent to participate in the study will be requested to sign the informed consent form, and it will be made clear that they are free to withdraw from the study at any stage without risk of any negative consequences. For women with low levels of literacy, an impartial witness will be present during the entire informed consent process. Both the witness and the individual discussing the consent will sign and date the consent form. The contact details of the local investigators, including telephone numbers, will be made available to the participants should they require further information and assistance.

The study does not involve any intervention. Participants will not experience any direct and/or immediate benefits for participating in the study. However, this study aims to collection information to inform the implementation phase of a project to reduce unnecessary caesarean section which ultimately has the potential to improve the quality of care around childbirth in the future. Study participants and other women using or intending to use facilities for childbirth will benefit from the increased scientific knowledge on this topic, which will ultimately promote high-quality, woman-centred care in the facilities. Improving societal knowledge on this topic will improve the awareness of quality of care in maternity services.

Other safeguards include the use of unique participant numbers on all study forms, and ensuring that interviewers and data collectors are not current or previous employees of the study facility. Study participants will receive a reimbursement to cover their transportation to the venue of the interview, if applicable. The value of this payment will be determined in consultation with the research teams, to ensure that it does not constitute an inducement. Refreshments will be available during focus group discussions.

This protocol has been adapted for implementation in Argentina, Burkina Faso, Thailand, and Viet Nam as part of the "Appropriate use of Caesarean section through **QUALI**ty **DEC**ision-making by women and providers" (QUAL-DEC) project [38]. In the QUALI-DEC project, we have used the readiness assessment, document review, and adapted the qualitative modules specific to the intervention components: (1) opinion leader education to implement clinical practice guidelines, (2) labour companionship, (3) decision-analysis tool for mode of birth, and (4) caesarean section audit and feedback.

## Supplementary information

**Additional file 1.** Interventions that may reduce the rate of unnecessary caesarean sections. Twelve potential interventions that may influence use of caesarean section were identified through the WHO guideline development. This file provides a description of each intervention, theory of change, and supporting evidence for each potential intervention.

**Additional file 2.** Data collection form: document review.

**Additional file 3.** Data collection form: readiness form assessment.

**Additional file 4.** Qualitative module 0: Background and understanding context of preferences for mode of delivery.

**Additional file 5.** Qualitative module 1: Prenatal education and support.

**Additional file 6.** Qualitative module 2: Decision-aids for mode of birth.

**Additional file 7.** Qualitative module 3: Psychosocial support for women with fear of childbirth.

**Additional file 8.** Qualitative module 4: Labour companionship.

**Additional file 9.** Qualitative module 5: Public dissemination of caesarean section rates at a facility-level.

**Additional file 10.** Qualitative module 6: Audit and feedback including external review of labour and delivery records and use of Robson classification as a feedback too.

**Additional file 11.** Qualitative module 7: Mandatory second opinion before conducting a caesarean section.

**Additional file 12.** Qualitative module 8: In-service training and implementation of clinical practice guidelines.

**Additional file 13.** Qualitative module 9: Equalising physician pay for vaginal and caesarean birth.

**Additional file 14.** Qualitative module 10: Opinion leader education.

**Additional file 15.** Qualitative module 11: Setting a goal for caesarean section rate at a facility-level.

**Additional file 16.** Qualitative module 12: Policies limiting legal liability and malpractice lawsuits.

## Data Availability

All data generated or analysed during this study are included in this published article and its supplementary information files.

## Abbreviations

FGD: Focus group discussion
IDI: In-depth interview
WHO: World Health Organization
