# Supplementary material for: Optimising the use of caesarean section: a generic formative research protocol for implementation preparation
Source: Reprod Health. 2019 Nov 19;16:170. doi: 10.1186/s12978-019-0827-1 (PMC6862737; doi:10.1186/s12978-019-0827-1)
Supplement: Supplementary file 1 — Additional file 1. Interventions that may reduce the rate of unnecessary caesarean sections. Twelve potential interventions that may influence use of caesarean section were identified through the WHO guideline development. This file provides a description of each intervention, theory of change, and supporting evidence for each potential intervention. [file 12978_2019_827_MOESM1_ESM.docx]

**Additional file 1. Interventions that may reduce the rate of unnecessary caesarean sections**

| **Intervention** | **Background and description of the intervention** | **Theory of change** | **Supporting evidence from WHO guideline [1]** |
| --- | --- | --- | --- |
| ***Interventions targeted at women, communities, and/or to the general public*** | | |  |
| **Prenatal education and support**  **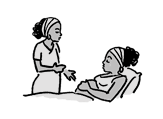** | Health education for women about pregnancy and childbirth is an essential component of antenatal care [1, 13]. Health topics that may be include nutrition, physical activity, breastfeeding, what to expect during labour and childbirth, comfort techniques during labour, and what to expect and how to manage in the postpartum period including caring for herself and the baby [1]. Research has suggested that women’s preference for mode of birth evolves during pregnancy [15]. Women form opinions about their preferred mode of birth early in their pregnancy, and preferences for caesarean section may increase from early pregnancy, through late pregnancy and the early postpartum periods [15]. Health education about mode of birth during antenatal education may help inform women’s decisions about how they plan to give birth.  Women can also learn breathing and relaxation techniques to use during labour and birth, and this can help them to give birth spontaneously [16, 17]. These could include guided relaxation or meditation to visualise the process of labour with a calm mindset, and may target all women, nulliparous women, or women with anxiety. | Good health literacy, for example “people’s ability to obtain, process, understand and judge the reliability of health information”, can contribute to people making informed choices about their health, improved health outcomes and reduced healthcare costs [18]. However, people may not be able to assess the reliability of information about treatment effects, and may tend to overestimate treatment benefits and underestimate treatment harms [18]. Poor health literacy is associated with sub-optimal use of healthcare services and poor health outcomes [18]. . Including educational materials about mode of birth during antenatal care and educational sessions may be an important way to improve women’s health literacy about mode of birth. Improving women’s health literacy about all modes of birth. | Health education for women is an essential component of antenatal care. The WHO Guideline concluded that childbirth education workshops may reduce the caesarean section rate compared to routine maternity care as well as increase spontaneous vaginal birth compared to routine maternity care (Low-certainty evidence) .  Qualitative evidence indicates that women think that learning new information about birth can be empowering. Women want educational tools (childbirth training workshops, booklets, decision-aids) and welcome multiple formats (although information on paper is ultimately needed for reflection with family, friends and health-care professionals).  Based on this evidence, prenatal education and support is recommended by WHO in the context of targeted monitoring and evaluation. |
| **Decision-aids for mode of birth**  **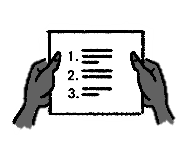** | Shared models of decision-making regarding the mode of birth between pregnant women and their healthcare providers are increasingly preferred, compared to obstetrician-, midwife-, or nurse-dominated decisions [20]. For pregnant women with a previous caesarean section, there may not be a single best choice about the mode of birth for their current pregnancy. Decision-aids are an intervention that can be used to present evidence about potential benefits and harms for different treatment options, and can be used to help pregnant women make deliberated and specific choices [21]. Decision-aids are designed to supplement (not replace) regular counselling and discussions with healthcare providers [21]. They provide detailed, specific and personal options and outcomes in order to prepare women to make the decision about the mode of birth [21]. Informed decision-making around the optimal mode of birth for women with previous caesarean section requires consideration of the benefits and risks of repeat caesarean section, trial of labour, vaginal birth after caesarean section and the woman’s perspectives and preferences for her childbirth experience [20]. In this case, decision-aids can help to provide comprehensive, balanced, and unbiased information, which may help to decrease anxiety about decision-making [21]. However, there are no published randomized trials on the effect of decision aids on women without a previous caesarean section. | Decision-aids can be used before, during or after a clinical contact, in order to help women to become more informed and active in their care [21]. If women are provided with the decision-aids before a clinical contact, they may have more time to absorb the information and prepared to discuss the options [21]. Shared decision-making about mode of birth refers to healthcare providers and women making decisions together about women’s childbirth experiences, and is considered to be at the core of woman-centred care [21]. Standardised decision-aids may also reduce the likelihood of clinician preferences dictating a woman’s care pathway [21]. Decision-aids may therefore assist women and providers to make decisions that are grounded in women’s values and preferences and account for risks and benefits [21]. | *Nulliparous women:* no studies (evidence gap).  *Women with a previous caesarean section:*  Computer-based decision aids (information programme, decision analysis) and decision-aid booklet showed no differences in caesarean section rates when compared with routine maternity care (Moderate-certainty evidence).  Qualitative evidence indicates that women think that learning new information about birth can be empowering. Women want educational tools (childbirth training workshops, booklets, decision-aids) and welcome multiple formats (although information on paper is ultimately needed for reflection with family, friends and health-care professionals).  Based on this evidence, decision-aids for mode of birth are recommended by WHO in the context of targeted monitoring and evaluation. |
| **Psychosocial support for women with fear of childbirth**  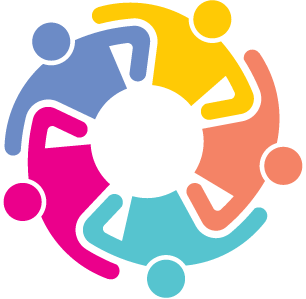 | Research has suggested that fear of labour and childbirth may affect between 5 to 20 percent of all women [22], and may be expressed as physical complaints, nightmares, and difficulty concentrating [23]. There are several factors associated with a fear of childbirth, including nulliparity, young maternal age, pre-existing psychological problems, lack of social support, and history of abuse or previous childbirth complications [22]. Fear of labour and childbirth has been shown to be a contributing factor to maternal request for caesarean section [24-26]. For example, a study conducted in Norway found that women who feared childbirth were more than four times as likely to have a preference for caesarean section, and more than twice as likely to receive a caesarean section, compared to women who did not fear childbirth [26]. Identifying and managing maternal fear and anxiety may therefore have an impact on reducing maternal request for caesarean section, in addition to improving maternal and infant morbidity [23]. | Research has shown that fear of childbirth is associated with women’s preferences for caesarean section [22]. Treating anxiety and fear of childbirth may reduce perceived fear, anxiety and stress, and help a woman have a more positive pregnancy experience [27]. A reduction in women’s fear of childbirth may therefore result in a reduction of maternal request for caesarean section, which may have an overall impact on elective caesarean section rates. | Evidence suggests that psychoeducation may increase spontaneous vaginal birth compared to routine maternity care.  Qualitative evidence indicate that women want emotional support alongside the communication of facts and figures about birth.  Based on this evidence, group therapy for women with fear of childbirth is recommended by WHO in the context of targeted monitoring and evaluation. |
| **Labour companionship**  **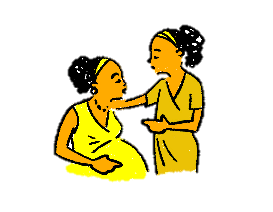** | Research shows that women value and benefit from the presence of a support person during labour and childbirth [28]. The person providing support may be someone from the woman’s social network (such as a partner, family member or friend), hospital staff (such as nurses or midwives), or women who were not hospital staff and had no personal relationship to the woman in labour (such as a doula) [28]. The type of support provided may include emotional support (continuous presence, reassurance and praise), and information about labour progress [28]. It may also include advice about coping techniques, comfort measures (such as a comforting touch, massage, encouraging mobility, and promoting adequate fluid intake and output), and speaking up when needed on behalf of the woman [28]. When women do not receive continuous support throughout labour and childbirth, there are concerns that women’s experiences of labour and birth may become dehumanised [28]. | Two complimentary theoretical explanations have been proposed to explain the effects of companionship [28]. Both theories hypothesise that support during labour and childbirth enhances the woman’s physiology and her feelings of control and competence, thus reducing reliance on medical interventions [28]. The first explanation hypothesises that modern obstetric care frequently subjects women to institutional routines, high rates of intervention, unfamiliar personnel, lack of privacy and other conditions that a women may experience as harsh [28]. The provision of support from a companion may help to buffer the woman from these stressors. The second explanation hypothesises that support during labour enhances fetopelvic relationships (such as by encouraging mobility and effective use of gravity), and decreases the woman’s stress responses [28]. Support from a companion throughout labour and birth may thus reduce a woman’s anxiety and fear. | Evidence from one updated Cochrane review indicate that continuous one-to-one intrapartum support (by nurse-midwives, lay companion and doulas**) may reduce caesarean birth and improve other birth outcomes [28].  **Intervention not specifically designed to reduce caesarean births. Further studies are required to confirm observed benefits in areas with high caesarean section rates.  Labour companionship has been recommended in three WHO guidelines related to intrapartum care [14, 29, 30]. |
| **Public dissemination of CS rates**  **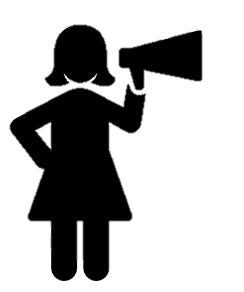** | It is increasingly common to publicly release information about health system and health facility performance [31]. This performance data may have an impact on changing the behaviours of healthcare providers, consumers, and professionals that can improve performance and quality of care [31]. Some evidence suggests that publicly releasing performance data may stimulate quality improvement activities at the hospital level [32]. In the context of caesarean section, public dissemination of caesarean section rates may influence the behaviours of both healthcare providers and women, and may promote quality improvement activities within hospitals and more appropriate decision-making for caesarean section. | The impact of the public release of performance data may operate through several different mechanisms. Public dissemination may influence providers to improve their performance, through either a selection or change pathway [31, 33]. In a selection pathway, consumers, patients, and other stakeholders can select hospitals based on the best potential outcomes [31]. While this would not change the quality of care provided at an individual hospital, it may stimulate quality improvement [31]. In a change pathway, the public dissemination may influence providers and hospitals to prioritise quality improvement by changing professional culture, clinical practice, and other structural changes [31]. | Evidence gap: No eligible study on this prespecified intervention identified; studies evaluating the effects of this intervention are needed. |
| ***Interventions targeted at healthcare providers*** | | |  |
| **Audit and feedback, including external review of records and use of Robson classification** 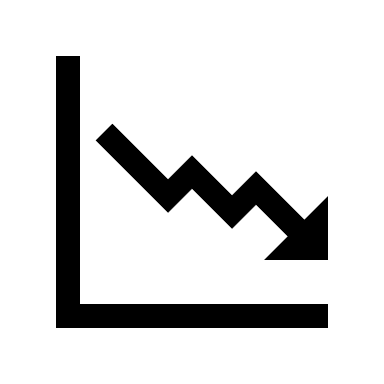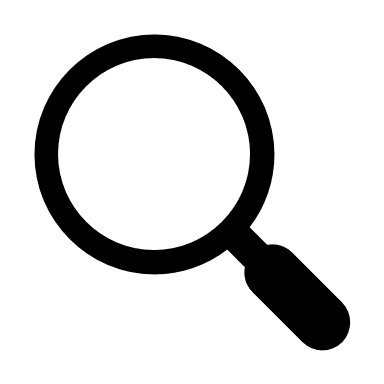 | The purpose of an audit and feedback process is to encourage individuals and teams to follow professional standards or targets. During an audit and feedback process in healthcare, an individual’s professional practice and/or performance is measured and compared to targets or professional standards [34]. The results of this comparison are fed back to the individual by either a colleague, supervisor or third party, in the form of verbal or written communication [34]. Typically, this also involves a discussion about areas for improvement, and is often done in conjunction with other interventions such as training, leadership, or quality improvement meetings [34]. Feedback may be given once, a fixed number of times, or in a recurring manner.  External review of labour and birth records can help to ensure that caesarean sections are performed for clinically valid reasons, and identify priority areas for coaching, training, and support for healthcare providers [35]. External review can be used as a teaching tool to help healthcare providers agree on the operationalisation of indications for caesarean section in their context.  Furthermore, to better understand contributing factors for the increasing trend and implement measures to reduce or increase caesarean section as needed, tools are needed to monitor and compare caesarean section rates over time in the same setting and between different settings [36]. A WHO-led systematic review [37] concluded that the Robson 10 group classification system [38] was the best classification system to apply internationally due to its simplicity, clinically-relevant data, accountability, replicability, verifiability, and woman-centeredness. The Robson classification system is a classification system for all women giving birth in a specific setting (not only for women giving birth by caesarean section), and prospectively identifies groups of women who are admitted for childbirth. Each of the ten categories are mutually exclusive and totally inclusive, and every woman admitted for childbirth can be immediately classified based on a few obstetrical characteristics that are routinely collected in healthcare facilities worldwide [36]. Classifying women using this model allows for comparison and analysis of caesarean section rates within and across the different groups of women, as well as comparisons to other facilities and countries globally in a standardised way [36, 39, 40]. | Many theories exist to explain how audit and feedback may lead to quality improvement, including [34]:   - Changing individuals’ beliefs and awareness about clinical practice and consequences; - Changing social norms; - Improving self-efficacy; - Directing attention to a specific set of tasks.   Healthcare providers may be motivated to improve their practices if it is identified that their clinical skills or practice are inconsistent with clinical guidelines or their peers [34]. Connecting the audit and feedback to clear targets and an action plan is likely to contribute to sustained quality improvement [34]. Furthermore, in order to design and implement effective measures to optimise caesarean section rates, tools are needed to monitor and compare caesarean section rates in a specific context over time, and between different contexts. Historically, caesarean section rates have been monitored by calculating the overall percentage of births by caesarean section or by indication for caesarean section [37]. However, variations in an “overall caesarean section rate” between different contexts and over time are difficult to interpret and compare. A standardized, reliable, consistent, and action-oriented classification system would help to monitor and compare caesarean section rates at the facility level [3]. The classification system would provide a clear understanding of where, when, why, how, and on whom caesarean sections are being performed, which is a critical step to design and implement effective strategies to reduce or increase the rate of caesarean sections in order to improve maternal and perinatal health [36, 37]. | High-certainty evidence shows that implementation of guidelines combined with audit and feedback slightly reduces caesarean section rates in women with low-risk pregnancies.  High-certainty evidence shows that implementation of guidelines combined with audit and feedback slightly reduces assisted vaginal birth.  Qualitative evidence indicates that lack of training, skills or experience is a barrier to change and thus it is important that interventions have a training component tailored to local needs.  Based on this evidence, implementation of clinical guidelines combined with audit and feedback is recommended by WHO [1]. The use of Robson classification is also recommended by WHO to monitor and compare caesarean section rates over time [39]. |
| **Mandatory second opinion**  **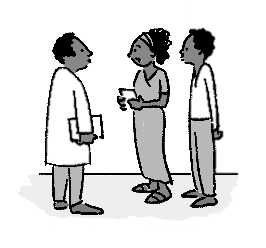** | Mandatory second opinion refers to the practice of having a physician providing a second opinion on the decision for a non-emergency caesarean section. The physician providing the second opinion should [1]:   1. Have clinical qualifications equal to or higher than the attending physician; 2. Be working in the same hospital; 3. Selected by the obstetrics department; and 4. Have agreed to follow the clinical guidelines, which provide the evidence base to inform the decision to conduct a caesarean section;   and the second opinion should be sought before conducting a non-emergency caesarean section. In this intervention, the physician providing the second opinion assesses each clinical case using pre-specified clinical guidelines and decision-flowcharts for the primary indications for caesarean section [41]. Both physicians discuss the clinical case using the decision-flowcharts to guide their discussion, and the physician providing the second opinion makes the final decision [41]. | The proposed theory of change for the impact of mandatory second opinion on caesarean section rates is that the consultation would impact physicians’ attitudes towards indications for caesarean section. The consultation discussion includes the confirmation, re-diagnosis, and options for managing of clinical indications for caesarean section, and thus may influence diagnoses and management plans. Furthermore, mandatory secondary opinion may reduce the likelihood of the use of caesarean section based on provider convenience or scheduling. | High-certainty evidence shows that implementation of evidence-based clinical practice guidelines combined with mandatory second opinion slightly reduces caesarean section rates.  The WHO Guideline Development Group noted that, although the effect size for this intervention is small, it might still translate into important impact on caesarean section rates, particularly in settings with adequate resources and high caesarean section rates.  Based on this evidence, audit and feedback combined with mandatory second opinion is recommended by WHO. |
| **In-service training and implementation of clinical practice guidelines**  **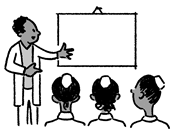** | Across the world, all groups of decision-makers (healthcare providers, patients, administrators, and policy-makers) are facing challenges in using research evidence to inform healthcare decisions. In the context of maternal health, most of the estimated 300,000 maternal deaths are preventable [42]. This highlights the importance of ensuring the availability of high quality of care, including the implementation of interventions known to be effective [43]. Adaptation and implementation of clinical and health systems guidelines is a critical aspect to improve quality of care and accountability. By guidelines, we are referring to recommendations for clinical or public health practice that provides options on what can be done in a specific situation in order to achieve the best outcomes [44]. Guideline recommendations can help end-users to select and prioritise across a range of potential interventions [44].  Typically, these guideline recommendations are adapted for use in a specific context, for example, in a specific health facility. The subsequent set of practical statements for implementing these best clinical practices may take the form of a clinician decision-making support tool, referred to as a clinical practice guideline or protocol. For example, this may include a clinical protocol for managing post-partum haemorrhage in health facility A, which would account for local constraints and opportunities including availability of drugs and health worker mix. Clinical practice guidelines can be useful tools to reduce patient harm by improving standardisation and communication. | Social influence theory can provide a framework for understanding how relationships and social processes can influence the successful implementation of clinical practice guidelines [45]. For example, the perceived values, preferences, attitudes, and opinions of peers and opinion leaders can influence attitudes of individual healthcare providers and their decisions to adopt guidelines into their practice [45]. Clinical practice implementation strategies that account for social influence theory may include components such as using opinion leaders, mass media education, and audit and feedback [45]. Furthermore, engagement with healthcare providers using consultative and consensus processes is more likely to result in behaviour change, and represents how social influence strategies can be used to build ownership [45]. | High-certainty evidence shows that implementation of guidelines combined with audit and feedback slightly reduces caesarean section rates in women with low-risk pregnancies.  High-certainty evidence shows that implementation of guidelines combined with audit and feedback slightly reduces assisted vaginal birth.  Qualitative evidence indicates that lack of training, skills or experience is a barrier to change and thus it is important that interventions have a training component tailored to local needs.  Based on this evidence, implementation of clinical guidelines combined with audit and feedback and/or mandatory second opinion is recommended by WHO. |
| **Equalising pay for CS and vaginal birth including VBAC**  **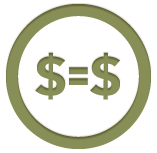** | In many settings, providers are paid a higher rate for conducting caesarean sections, compared to vaginal births, partially due to the perceived additional resources needed to conduct an operative birth. It has been hypothesised that this favourable reimbursement scheme has contributed to the rising caesarean section rates. In response, some governments and insurers have enacted policies to equalise physician payment for caesarean and vaginal births. However, these policy interventions have often been met with mixed results [46]. A key limitation in previous research is the inability to distinguish between elective and clinically indicated caesarean sections, which compromises the ability to detect the impact of equalised pay [47]. | Economic theories of provider behaviour posit that when healthcare providers choose the type of services provided to their patients, cost-based reimbursement systems may result in too many services being provided [48]. In many settings, physicians are the dominant decision-makers for the level and type of services provided, including caesarean section, thus suggesting that supply-side incentives (such as equalising physician pay for vaginal and caesarean birth) may be more effective than demand-side incentives (e.g. that target women and their families to reduce demand for caesarean section) [48]. Economic theories of provider behaviour also suggest that healthcare providers are not always acting as rational agents, due to the influence of the economic power of health facilities and perceived benefit at an individual-level [48]. Equalising pay for vaginal and caesarean birth may therefore reduce financial incentives to the healthcare provider, and may contribute to a reduction in unnecessary caesarean sections. | The WHO Guideline Development Group (GDG) noted that only two interrupted time series studies assessed this intervention, conducted in countries with different healthcare systems and resource capacities (Taiwan [China], the United States) – applicability in other settings is therefore uncertain. The certainty of evidence for caesarean section outcome was judged as very low in both studies (the effect on caesarean section rates is therefore uncertain). Despite these uncertainties, the GDG noted that financial incentive remains a major determinant of caesarean births in all settings.  Given the uncertainties in the impact of financial strategies and their importance in caesarean births, the GDG recommended the implementation of financial strategies equalizing physician fees for vaginal births and caesarean sections only in the context of rigorous research examining the impact on caesarean births and exploring their acceptability to key stakeholders and the feasibility of their implementation. |
| **Opinion leader education**  **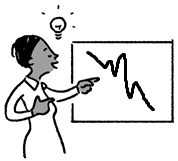** | Opinion leader education is an intervention that aims to change the culture and norms of healthcare provider peer groups using an influential individual to drive change. In the context of caesarean section, opinion leaders have been trained on emerging research and clinical recommendations related to optimising the use of caesarean section, as well as educational tools for behaviour change and quality improvement in a healthcare setting [1, 49]. For example, this might include how to implement trial of labour for women who have a previous caesarean section, in order to increase vaginal birth after caesarean section. Opinion leaders then lead the knowledge translation from published clinical recommendations to appropriate implementation in a specific healthcare setting. Opinion leaders are typically identified by their peers, based on individuals who best meet a set prespecified criteria [49], such as those who are knowledgeable, good communicators and humanistic [50]. | Evidence has demonstrated that the application of research findings and guideline implementation can be slow, and that traditional dissemination approaches (e.g.: publication of guidelines or journal articles) is unlikely to lead to changes in practice [50]. More recently, there has been greater awareness of other environmental factors that influence behaviours and practices, which need to be accounted for during implementation [50]. Research has demonstrated that healthcare providers share common beliefs and group norms with their peer groups, and that these norms can directly influence individual behaviours [50]. The concept of opinion leadership proposes that influential healthcare providers take advantage of the structure of these influential group networks to change norms and drive quality improvement in a healthcare setting. | High-certainty evidence shows that the use of local opinion leader education as a method to implement guidelines reduces rates of elective caesarean.  Based on this evidence, the use of opinion leaders for the facilitation of implementation of interventions such as on-site training in evidence-based clinical practice is recommended by WHO together with supportive supervision. |
| **Goal setting at a hospital level**  **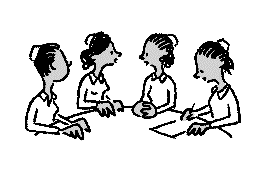** | Setting goals for caesarean section rates at a hospital level may help to reduce caesarean section rates for low-risk women. Hospital-specific goals may be a useful way to ensure the flexibility of an “ideal” caesarean section rate, as the target can be adjusted based on the level of hospital (e.g.: tertiary vs primary), and the characteristics of the population (e.g.: higher or lower risk women). Goals may be agreed upon by hospital leaders, in consultation with their staff and external stakeholders such as health managers or community groups. Goals may target specific Robson groups as opposed to overall caesarean section rate [36, 39]. Over time, goals and targets can progressively be reassessed and modified to reflect the dynamics of quality improvement initiatives and progress. | The process by which goals are identified and set inherently implies an expression of dissatisfaction with current conditions and desire to change or improve for the future [51]. Prior to setting goals, it is important to conduct a problem analysis to understand the issue in a given context, and select an intervention or interventions that will address the issue; this may be referred to as a problem-based goal-setting approach [52]. A key underpinning of goal-setting theories is the power of goals to motivate provider behaviour change in order to achieve the desired change or outcome [51]. Goals may motivate better individual task performance by inspiring behaviour change around the goal-relevant behaviours [51]. In the context of reducing caesarean section rates, goal setting at the hospital level may inspire, motivate, and stimulate progress [53] of individual providers and teams to focus on improving their performance and capacity to achieve the desired outcome. | Evidence gap: No eligible study on this prespecified intervention identified; studies evaluating the effects of this intervention are needed. |
| **Policies that limit legal liability and malpractice lawsuits**  **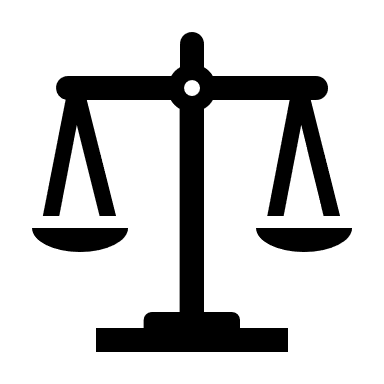** | Fears of legal liability and medical malpractice litigation may be a factor influencing rising caesarean section rates. These fears may influence providers to favour caesarean section over vaginal birth, as well as discourage vaginal birth after caesarean section (VBAC) [54]. In some settings, obstetricians pay more for liability insurance coverage and are sued more frequently, compared to physicians in other specialties [54]. Caesarean section may be seen as a protective mechanism to avoid litigation, rather than an alternative mode of birth when vaginal birth is not possible [54]. Evidence of the association between legal liability and caesarean section rates is mixed, as some studies have demonstrated a positive association [55-58], and others have demonstrated no relationship [59-61]. Mixed evidence of the association between legal liability and caesarean section may not be surprising given the variance in legal structures and practices across contexts. | Some research has demonstrated that obstetricians may view caesarean sections as a way to limit their exposure to malpractice lawsuits [54]. If policies were enacted to limit an obstetrician’s legal liability in the case of obstetric complications, then obstetricians may be less fearful of legal liability, and consequently reduce their use of caesarean section as a defensive measure against litigation. | Evidence gap: No eligible study on this prespecified intervention identified; studies evaluating the effects of this intervention are needed. |
