# Supplementary material for: Optimising the use of caesarean section: a generic formative research protocol for implementation preparation
Source: Reprod Health. 2019 Nov 19;16:170. doi: 10.1186/s12978-019-0827-1 (PMC6862737; doi:10.1186/s12978-019-0827-1)
Supplement: Supplementary file 2 — Additional file 2. Data collection form: document review. [file 12978_2019_827_MOESM2_ESM.docx]

## **Additional file 2. Document review**

**Instructions.** *This document review should be completed by a member of the research team, preferably with a clinical background or background in maternal health. Documents to be included in this review should be identified and prioritised within the research team and key stakeholders. Depending on the level of facility that will be targeted for change, the review may include documents such as national or state-level policies, reports, or strategic plans for maternal or reproductive health, or local policies or guidelines. The document review will help to identify contextually-specific areas for improvement at a policy and practice-level.*

**Part 1.** Content of any national, regional or state policies on clinical care during labour and childbirth.

*Please identify any relevant national, regional, or state policy documents or strategic plans related to maternal health, obstetrics and gynaecology, universal health coverage, or women’s health. Review each of these documents and complete the table below to describe (please add more rows as necessary):*

| **Document name** | **Report author, commissioner or administering body** | **Any mention of care during labour and childbirth (yes or no)** | **Supporting text related to care during labour and childbirth** |
| --- | --- | --- | --- |
|  |  |  |  |
|  |  |  |  |
|  |  |  |  |

**Part 2.** Content of any national/subnational clinical protocols or guidelines for managing labour and childbirth, including protocols for managing complications arising during labour and childbirth.

*Please identify any relevant national, regional, or state clinical protocols or guidelines for managing labour and childbirth, including complications. Review each of these documents and complete the table below to describe (please add more rows as necessary):*

| **Document name** | **Report author, commissioner or administering body** | **Any mention of clinical care during labour and childbirth (yes or no)** | **Supporting text related to clinical care during labour and childbirth** |
| --- | --- | --- | --- |
|  |  |  |  |
|  |  |  |  |
|  |  |  |  |

**Part 3.** Are there national level regulations that legislate the availability and implementation of clinical protocols or guidelines for managing labour and childbirth, including complications?

*Please identify any regulations below, or specify if no such regulations exist.*

|  |
| --- |

**Part 4.** Content of any national, regional or state frameworks or strategies to improve maternal and newborn health or quality of maternity services, related to caesarean section.

*Please identify any relevant national, regional, or state frameworks or strategies to improve maternal and newborn health or quality of maternity services and identify where and how caesarean section is included. Review each of these documents and complete the table below to describe (please add more rows as necessary):*

| **Document name** | **Report author, commissioner or administering body** | **Any mention of caesarean section (yes or no)** | **Supporting text related to caesarean section** |
| --- | --- | --- | --- |
|  |  |  |  |
|  |  |  |  |
|  |  |  |  |

**Part 5.** Content of any legal or regulatory framework or guidance regarding the use of caesarean section or other modes of birth at a national or subnational level.

*Please identify any relevant national, regional, or state legal or regulatory frameworks laws related to the use of caesarean section. Please state the law or legal frameworks below, or specify if no such law exists.*

|  |
| --- |

**Part 6.** Coverage and availability of maternal health care provision at the national/subnational level.

*Please identify any indicators related to the coverage and availability of the following: caesarean section, instrumental vaginal birth, epidural or other pharmacological pain management, non-pharmacological pain management, labour companionship. Please specify if no such indicators exist.*

|  |
| --- |

**Part 7. Data on caesarean section.**

*Please describe how data related to caesarean section is currently collected, analysed and reported at the national/subnational levels. Please specify if no such data exists, or is not regularly reported. Please add more rows as needed.*

| **National, subnational, and local level** | |  |
| --- | --- | --- |
| **Location (city, state)** | **Narrative summary of caesarean section data** | |
|  |  | |
|  |  | |
|  |  | |

**Part 8. Audit and feedback of caesarean section data.**

*Please describe what mechanisms are in place to review and discuss data related to caesarean section at the national/subnational levels. Please specify if no such mechanisms exist, or are not regularly conducted.*

| **District/regional level** |  |  |
| --- | --- | --- |
| **Location (city, state)** | **Narrative summary of audit and feedback for caesarean section data** | |
|  |  | |
|  |  | |
|  |  | |

| **National level** |  |
| --- | --- |
| **Narrative summary of audit and feedback for caesarean section data** | |
|  | |

**Part 9.** Caesarean section on maternal request

*Please provide a description of what guidance is in place regarding caesarean section on maternal request at the national/subnational levels. Please specify if no such guidance exists.*

|  |
| --- |

**Part 10.** Trial of labour and vaginal birth after caesarean section

*Please provide a description of what guidance is in place regarding trial of labour and/or vaginal birth after caesarean section at the national/subnational levels. Please specify if no such guidance exists.*

|  |
| --- |

**Part 11.** Cost of birth

*Please provide a description of the financial costs to the woman and her family for a vaginal birth and for a caesarean section, or to the health care system if publicly provided, or the insurance system.*

|  |
| --- |

**Part 12.** Healthcare provider remuneration

*Please provide a description of how much money the hospital/doctor/nurse/midwife will receive for a vaginal birth and for a caesarean section.*

|  |
| --- |

**Part 13.** Health system cost

*Please provide a description of how much it costs a hospital to perform a vaginal birth and a caesarean section.*

|  |
| --- |

**Part 14.** Health facility remuneration

*Please provide a description of how much money the health facility will receive for a vaginal birth and for a caesarean section.*

|  |
| --- |
