# Supplementary material for: Optimising the use of caesarean section: a generic formative research protocol for implementation preparation
Source: Reprod Health. 2019 Nov 19;16:170. doi: 10.1186/s12978-019-0827-1 (PMC6862737; doi:10.1186/s12978-019-0827-1)
Supplement: Supplementary file 3 — Additional file 3. Data collection form: readiness form assessment. [file 12978_2019_827_MOESM3_ESM.docx]

**Additional file 3. Readiness assessment**

*This activity is part of the readiness assessment, to explore factors to be assessed, considered and integrated into implementation plans. There are five components of this readiness assessment:*

1. *Inventory of physical space and readiness;*
2. *Health workforce and model of care;*
3. *Protocols and guidelines for managing clinical care during labour and childbirth;*
4. *Assessment of data availability and access for audit and feedback; and*
5. *Understanding of labour companionship in practice.*

*For each of the study health facilities, please have a member of the research team visit to conduct an observation of the labour ward and medical records. It may be most appropriate for this person to have some clinical knowledge. We expect that this activity will take approximately four to six hours to complete (depending on how busy the facility is). Prior to conducting the readiness assessment, please ensure that all members of the maternity care unit at each health facility are briefed on the purpose of the activity, what the readiness assessment will entail, and how they may be of assistance. This will help to ensure that the readiness assessment (and other research activities) will be welcomed by the unit.*

**Part 1. Inventory of physical space and resources**

*Please observe the physical space of the labour, delivery and postnatal wards. If these are in separate areas (e.g.: women in latent labour in a labour ward, women in active labour in a separate room/delivery ward, separate postnatal ward), please assess both areas according to all points below. Please provide a narrative description of the wards, as well as a visual depiction.*

Description of the physical space

|  |
| --- |

Description of the operating theatres (how many, general theatre/reserved for obstetrics, handwashing facilities)

|  |
| --- |

Description of any curtains, dividers or other means of protecting a woman’s privacy

|  |
| --- |

Description of the potential for crowding. For example, how many beds are present? Are they currently or usually full? What happens if there is overcrowding?

|  |
| --- |

Description of the visiting hours and allowable visitors (check if visually displayed and ask an administrator)

|  |
| --- |

Description of the accessibility to toilets or washrooms

|  |
| --- |

Description of overnight accommodation for family members/friends of women (check if visually displayed and ask an administrator)

|  |
| --- |

Description of infection control measures (e.g.: handwashing facilities, availability of soap/antibacterial wash, gloves, etc)

|  |
| --- |

Please sketch the physical space

|  |
| --- |

**Part 2.** Health workforce and model of care.

*Completing this section may require both observation of the labour ward and a discussion with staff, e.g.: a matron-in-charge or head of obstetrics.*

Description of the health workforce (number of personnel by cadre of provider)

|  |
| --- |

Description of the model of maternity care (e.g.: midwife-led, physician-led)

|  |
| --- |

Description of structure of shifts for each cadre of maternity staff (obstetricians, junior doctors, midwives, nurses, anaesthetists, etc)

|  |
| --- |

**Part 3.** Protocols and guidelines for managing clinical care during labour and childbirth.

*Completing this section may require both observation of the labour ward (e.g.: posters or signs) and a discussion with staff, e.g.: a matron-in-charge or head of obstetrics.*

Description of any clinical protocols or guidelines for managing *routine labour and childbirth care*

|  |
| --- |

Description of how any clinical protocols or guidelines for managing *routine labour and childbirth* care were developed or adapted, and updated? How are these clinical protocols/guidelines used?

|  |
| --- |

Description of any protocols or guidelines for managing *complications during labour or childbirth care* (e.g. postpartum haemorrhage, caesarean section, sepsis, obstructed labour)

|  |
| --- |

Description of how any protocols or guidelines for managing *complications during labour or childbirth care were* developed or adapted, and updated? How are these clinical protocols/guidelines used?

|  |
| --- |

**Part 4.** Assessment of facility medical records and data management systems

*Please review individual medical records to assess the information currently collected related to key obstetric variables at an individual level. Please also review any facility-level register, log book or other records to assess information currently collected related to key obstetric variables at a facility level. It may be helpful to discuss the medical and facility records with the staff, e.g.: a matron-in-charge or head of obstetrics. Collecting this information will help to inform the implementation of the Robson classification system, e.g.: to identify what data is already routinely collected, and what data may need to be added to routine data collection.*

Description of the annual or monthly number of births (vaginal/caesarean/instrumental), disaggregated where possible

|  |
| --- |

Description of the proportion of elective and emergency caesarean sections.

|  |
| --- |

Description of other information routinely collected about caesarean section (e.g. provider, morbidity)

|  |
| --- |

Description of any facility-level register, log book, or other records collating key obstetric variables at the facility-level. Please include whether this register is paper-based or electronic, when it is updated, and how often it is reported.

|  |
| --- |

Who is responsible for completing the facility-level register?

|  |
| --- |

How often is the facility-level register updated?

|  |
| --- |

How is the information about facility-level key obstetric variables and outcomes currently integrated into audit and feedback?

|  |
| --- |

Who is present during audit and feedback sessions, and who leads the sessions?

|  |
| --- |

Description of the health facility’s “decision-to-incision” time to perform a caesarean section

|  |
| --- |

Description of the medical record structure (e.g. electronic or paper), and who keeps the records (e.g. woman or provider)?

|  |
| --- |

Are any summary hospital-level reports produced regularly from the medical records? If so, how often and what format?

|  |
| --- |

Is there any regular feedback of these reports to the providers? If so, how often and in what format?

|  |
| --- |

Description of key obstetric variables that are currently included on an individual’s medical record (parity, previous caesarean section, onset of labour, gestational age, fetal presentation or lie, number of foetuses).

|  |
| --- |

Description of the consistency of reporting for these indicators (e.g.: consistently reported across all records reviewed, some data missing – be specific).

|  |
| --- |

*Review of medical records to assess if key obstetric variables needed for Robson classification are correctly and consistently reported at an individual level. For each variable, please (1) ask the administrator how it is reported, and (2) observe a subset of records to assess how variable is actually reported (e.g. 5-10 medical records).*

| **Parity** | |
| --- | --- |
| **Administrator response** | **Observation of records** |
|  |  |

| **Previous caesarean section** | |
| --- | --- |
| **Administrator response** | **Observation of records** |
|  |  |

| **Onset of labour (spontaneous, induced, no labour/pre-labour caesarean section)** | |
| --- | --- |
| **Administrator response** | **Observation of records** |
|  |  |

| **Gestational age (preterm <37 weeks, term > 37 weeks)** | |
| --- | --- |
| **Administrator response** | **Observation of records** |
|  |  |

| **Fetal presentation or lie (cephalic, breech, transverse)** | |
| --- | --- |
| **Administrator response** | **Observation of records** |
|  |  |

| **Number of fetuses (singleton, multiple)** | |
| --- | --- |
| **Administrator response** | **Observation of records** |
|  |  |

Who is responsible for completing the individual-level medical records? Does anyone else check for consistent and correct reporting?

|  |
| --- |

Based on the observations and record assessment, what would you consider to be the most appropriate method of implementation of the Robson classification system (manually, using a spreadsheet or automatic calculator, or via electronic records)? Please explain.

|  |
| --- |

Based on the observations and record assessment, what would you consider to be the most appropriate reporting schedule (weekly, bi-weekly, monthly), and why?

|  |
| --- |

[*Ask the administrator*] What type of person would be the most appropriate facility-level champion to implement the Robson classification system, and why? [e.g.: type of provider, what skills this person would have]

|  |
| --- |

[*Ask the administrator*] Who is the best person to *record* data for Robson classification, and why?

|  |
| --- |

[*Ask the administrator*] Who is the best person to *analyse* data for Robson classification, and why?

|  |
| --- |

[*Ask the administrator*] Who is the best person to *report and present* data for Robson classification, and why?

|  |
| --- |

**Part 5.** Understanding of labour companionship in practice

*Completing this section may require both observation of the labour ward and a discussion with staff, e.g.: a matron-in-charge or head of obstetrics. If companionship is not currently allowed at the facility, please specify below.*

Description of who is currently allowed to act as a companion for the woman

|  |
| --- |

Description of for what periods of time companionship is offered (e.g.: from admission to discharge, during labour but not childbirth, only at childbirth)

|  |
| --- |

Description of the roles that companions usually undertake (e.g.: emotional support, providing food/water/tea to the woman, supporting staff)

|  |
| --- |

Description of how staff currently interact with companions

|  |
| --- |

Existence and content of any orientation materials, protocols, or guidelines related to how staff should work with companions, or on the role of companions. If no materials exist, please state this.

|  |
| --- |

Any other feedback, observations or reflections

|  |
| --- |
