# Supplementary material for: Optimising the use of caesarean section: a generic formative research protocol for implementation preparation
Source: Reprod Health. 2019 Nov 19;16:170. doi: 10.1186/s12978-019-0827-1 (PMC6862737; doi:10.1186/s12978-019-0827-1)
Supplement: Supplementary file 4 — Additional file 4. Qualitative module 0: Background and understanding context of preferences for mode of delivery. [file 12978_2019_827_MOESM4_ESM.docx]

# **Qualitative module 0: Background and understanding context of preferences for mode of delivery**

## **Overview**

### *Background*

Caesarean section is a surgical procedure that can effectively prevent maternal and newborn mortality when used for medically indicated reasons [1]. Rising caesarean section rates are a global problem that affect high-, middle-, and low-income countries across all geographical regions [1, 2]. The causes of increased rates vary across and within contexts, and may include differences in professional practices, fear of medical litigation, changes to the characteristics of the population (e.g.: increasing prevalence of obesity, or increasing proportion of older women or multiple births), as well as economic, organisational, and sociocultural factors such as generational shifts in work and family responsibilities, women’s increasing desire to determine how and when their baby is born, and physician preferences [1, 3-5]. Sustained increases in caesarean section rates are a major public health concern and there is an urgent need of evidence-based guidance to address this trend [1].

Beyond clinical indications for caesarean section, decisions about mode of delivery are influenced by many factors including women’s preferences, healthcare provider preferences, organisation of care within health facilities, and societal influences. Understanding how these multiple dimensions influence a woman’s mode of delivery is an important first step towards optimising caesarean section rates within a population.

In many settings, qualitative research has already been conducted to understand preferences for mode of delivery, and the individual, family, provider, and societal influences that shape these preferences. This previous research may have already identified the major drivers of the increase upon which you may want to act. If this type of research has already been conducted in your setting, ***then this module may not be necessary***. However, if no such research has been conducted in your setting, then this module will be an important starting point to understand contextual factors influencing rising caesarean section rates. Results from this module will also provide important information about the most critical areas to focus interventions to reduce unnecessary caesarean section.

## **Participants for qualitative research**

| **Data collection methods and participants** | | |
| --- | --- | --- |
| Population | In-depth interview (IDI) | Focus group discussion (FGD) |
| Women |  | 🗸 |
| Healthcare providers  (midwives/nurses, doctors) | 🗸 |  |
| Healthcare administrators  (matron-in-charge, medical director) | 🗸 |  |

| **Population of women** | | |
| --- | --- | --- |
| Nulliparous | Multiparous with previous CS | Multiparous without previous CS |
| 🗸 | 🗸 | 🗸 |

## **Resources and estimated time required to complete this module**

- Trained research assistants
- Audio recorders and notebooks for field notes
- Informed consent forms
- Private room for interview
- Focus group discussions with women: 30-45 minutes
- Interviews with healthcare providers and administrators: 20-30 minutes

**References**

1. World Health Organization. WHO recommendations on non-clinical interventions to reduce unnecessary caesarean sections. Geneva, Switzerland: World Health Organization; 2018.

2. Betran AP, Ye J, Moller AB, Zhang J, Gulmezoglu AM, Torloni MR. The Increasing Trend in Caesarean Section Rates: Global, Regional and National Estimates: 1990-2014. PLoS One. 2016;11(2):e0148343.

3. Mi J, Liu F. Rate of caesarean section is alarming in China. Lancet. 2014;383(9927):1463-4.

4. Zwecker P, Azoulay L, Abenhaim HA. Effect of fear of litigation on obstetric care: a nationwide analysis on obstetric practice. Am J Perinatol. 2011;28(4):277-84.

5. Lin HC, Xirasagar S. Institutional factors in cesarean delivery rates: policy and research implications. Obstetrics and gynecology. 2004;103(1):128-36.

## **Focus group discussion guide for women**

*Interviewer: This section of the discussion is about how women give birth in your community, for example through vaginal birth or caesarean section. I would like to ask you some questions about what women in your community think about the different ways to give birth.*

1. Could you tell me about your childbirth experience? How many times have you given birth, and were they vaginal births or caesarean sections?
2. Where do women in your community get information about childbirth?
   1. How do women make decisions on whether to have a caesarean birth or vaginal birth?
   2. What type of people might influence women’s decisions about whether to have a caesarean birth or vaginal birth?
      1. *Probe:* How does her husband or partner influence her decision?
      2. *Probe:* How does her family influence her decision?
      3. *Probe:* How do her friends influence her decision?
      4. *Probe:* How does her doctor influence her decision?
3. In your community, do women prefer to have caesarean section or vaginal birth? Please explain.
   1. Why do you think some women in your community prefer vaginal birth?
   2. Why do you think some women in your community prefer caesarean section?
   3. What do women in your community think about caesarean section as a mode of childbirth?
   4. What are some of the positive aspects of caesarean section?
   5. What are some of the negative aspects of caesarean section?
   6. What are some of the positive aspects of vaginal births?
   7. What are some of the negative aspects of vaginal births?
4. Do you think that there are high numbers of caesarean sections in your community?
   1. *Probe: if yes –* is this a problem? Why or why not?
5. Do you have any other comments or feedback about women’s preferences for vaginal birth or caesarean section?

## **Interview guide for providers and administrators**

*Interviewer: This section of the interview is about preferences for mode of delivery and different influences on how women give birth in your health facility.*

1. As a clinician, do you prefer for women to give birth vaginally or by caesarean section? Why?
   1. What are some of the benefits of caesarean section?
   2. What are some of the benefits of vaginal birth?
   3. What are some of the challenges with caesarean section?
   4. What are some of the challenges with vaginal birth?
   5. Which do you think is safer: vaginal birth or caesarean section? Why?
2. In your facility, do you think that women prefer to give birth by caesarean section or vaginally? Please explain.
3. In your health facility, how are decisions made about whether a woman will give birth vaginally or by caesarean section?
   1. Who is involved in making the decision, and what roles do they play?
   2. In your health facility, what are some of the clinical indications for caesarean section?
   3. Other than clinical indications for caesarean section, what factors might influence if a woman has a caesarean section?
4. In your health facility, how do you manage women who request to have an elective caesarean section?
   1. Why do you think women may request to have an elective caesarean section?
   2. Who do you think influences women’s decisions to have an elective caesarean section?
5. What is your perception of women giving birth vaginally after a previous caesarean section? Please explain.
   1. In your health facility, how do you manage pregnant women who have had a previous caesarean section?
   2. How do you feel about allowing women a trial of labour (TOL) after previous caesarean section?
   3. What factors might make you more or less likely to allow a woman a trial of labour after previous caesarean section?
6. In your opinion, are high rates of caesarean section a problem in your health facility? Why or why not?
   1. *Probe if yes:*
      1. Why do you think there are high rates of caesarean section in your facility?
      2. Do you think that the caesarean section rate in your facility can be reduced? Why or why not?
      3. Do you think that the caesarean section rate in your facility should be reduced? Why or why not?
      4. What are the barriers to reducing high rates of caesarean section in your facility?
      5. What could be done to reduce high rates of caesarean section in your facility?
   2. *Probe if no:*
      1. Do you think that the caesarean section rate in your facility could be reduced? Why or why not?
      2. Do you think that the caesarean section rate in your facility should be reduced? Why or why not?
      3. What are the barriers to reducing high rates of caesarean section in your facility?
7. Do you have any other comments or feedback about preferences for and decision-making around vaginal birth or caesarean section?
