# Supplementary material for: Optimising the use of caesarean section: a generic formative research protocol for implementation preparation
Source: Reprod Health. 2019 Nov 19;16:170. doi: 10.1186/s12978-019-0827-1 (PMC6862737; doi:10.1186/s12978-019-0827-1)
Supplement: Supplementary file 5 — Additional file 5. Qualitative module 1: Prenatal education and support. [file 12978_2019_827_MOESM5_ESM.docx]

# **
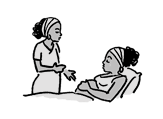
Qualitative module 1: Prenatal education and support**

## **Overview of intervention**

### *Background*

Health education for women about pregnancy and childbirth is an essential component of antenatal care (1, 2). Health topics that may be covered during antenatal care include nutrition, physical activity, breastfeeding, what to expect during labour and childbirth, comfort techniques during labour, and postpartum care (1). Research has suggested that women’s preference for mode of delivery evolves during pregnancy (3). Women form opinions about their preferred mode of delivery early in their pregnancy, and preferences for caesarean section may increase from early pregnancy, through late pregnancy and the early postpartum periods (3). This suggests that health education about mode of delivery during antenatal care may be appropriate to help inform women’s decisions about how they will give birth.

During antenatal care, women can also receive training about breathing and relaxation techniques to use during labour and birth (4, 5). This training may include sessions of guided relaxation or meditation to visualise the process of labour with a calm mindset, and may target all women, nulliparous women, or women with anxiety.

### *Supporting evidence*

A Cochrane intervention review synthesised evidence from 29 randomised trials on non-clinical educational interventions to reduce unnecessary caesarean sections, including interventions targeted at women (6). Twelve trials compared specific educational interventions to usual practice, and three trials compared different formats of educational interventions (6). The studies were conducted in high-income countries (nine studies) and middle-income countries (six studies) (6). Heterogeneity of the data on educational interventions precluded meta-analysis (6). Educational topics included in the interventions were psychoeducation, prenatal education for male partners, natural childbirth preparation, relaxation training, pelvic floor muscle training, intensive group therapy, and decision-aids (1, 6). Interventions were delivered in different formats including role play, interactive decision-aids, and individualised prenatal education (6).

Qualitative evidence synthesis suggests that women value information and educational interventions on mode of birth, as it can be empowering when the content is not anxiety-provoking (7). These interventions are particularly acceptable when they are provided alongside emotional support, and are used to inform more meaningful dialogue with health professionals (7).

Based on this evidence, prenatal educational interventions and support programmes are recommended to reduce caesarean births only with targeted monitoring and evaluation (1).

## **Theory of change**

Good health literacy, for example “people’s ability to obtain, process, understand and judge the reliability of health information”, can contribute to people making informed choices about their health (8). However, people may not be capable of assessing the reliability of information about treatment effects, and may tend to overestimate treatment benefits and underestimate treatment harms (8). Poor health literacy is associated with sub-optimal use of healthcare services and poor health outcomes (8). Improved health literacy, including the ability to make informed judgements about treatment effects, risks and benefits, can contribute to improved health outcomes and reduce healthcare costs (8, 9). Including educational materials about mode of delivery during antenatal care may be an important way to improve women’s health literacy about mode of delivery. Improving women’s health literacy about mode of delivery, including the benefits and risks of both caesarean section and vaginal birth, may be an important step towards improving preparation for labour and understanding of physiological vaginal birth, as well as reducing unnecessary caesarean section.

## **Participants for qualitative research**

| **Data collection methods and participants** | | |
| --- | --- | --- |
| Population | In-depth interview (IDI) | Focus group discussion (FGD) |
| Women |  | 🗸 |
| Healthcare providers  (midwives/nurses, doctors) | 🗸 |  |
| Healthcare administrators  (matron-in-charge, medical director) | 🗸 |  |

| **Population of women** | | |
| --- | --- | --- |
| Nulliparous | Multiparous with previous CS | Multiparous without previous CS |
| 🗸 | 🗸 | 🗸 |

## **Resources and estimated time required to complete this module**

- Trained research assistants
- Audio recorders and notebooks for field notes
- Informed consent forms
- Private room for focus group
- Focus group discussions with women: 30-40 minutes
- Interviews with healthcare providers and administrators: 15 minutes

| **Guiding principles** Interventions to include education on mode of delivery in antenatal care should be based on the following guiding principles:   1. **Ensuring autonomy, agency and choice**: All women should be provided with the information, education and means to make informed choices about their mode of delivery. They should be provided with resources to inform these choices that include information about different modes of delivery, and the benefits and risks of different modes. 2. **Community participation**: Participatory approaches should be used to assess the needs of women and girls, to ensure community ownership and engagement in developing and implementing educational materials that are culturally appropriate. 3. **Responsiveness of health systems**: Health systems need to be organized and managed so that they facilitate respect, protection and fulfilment of women’s sexual and reproductive health and rights. Provisions should be made to ensure privacy and confidentiality, and respect for women’s decision-making on mode of delivery. All involved in the care-giving process also need to understand their corresponding obligations and relevant standards of conduct. |
| --- |

**References**

1. World Health Organization. WHO recommendations on non-clinical interventions to reduce unnecessary caesarean sections. Geneva, Switzerland: World Health Organization; 2018.

2. World Health Organization. WHO recommendations on antenatal care for a positive pregnancy experience. Geneva, Switzerland: World Health Organization; 2016.

3. Long Q, Kingdon C, Yang F, Renecle MD, Jahanfar S, Bohren MA, et al. Prevalence of and reasons for women's, family members', and health professionals' preferences for cesarean section in China: A mixed-methods systematic review. PLoS Med. 2018;15(10):e1002672.

4. Bergstrom M, Kieler H, Waldenstrom U. Effects of natural childbirth preparation versus standard antenatal education on epidural rates, experience of childbirth and parental stress in mothers and fathers: a randomised controlled multicentre trial. Bjog. 2009;116(9):1167-76.

5. Bastani F, Hidarnia A, Montgomery KS, Aguilar-Vafaei ME, Kazemnejad A. Does relaxation education in anxious primigravid Iranian women influence adverse pregnancy outcomes?: a randomized controlled trial. J Perinat Neonatal Nurs. 2006;20(2):138-46.

6. Chen I, Opiyo N, Tavender E, Mortazhejri S, Rader T, Petkovic J, et al. Non-clinical interventions for reducing unnecessary caesarean section. The Cochrane database of systematic reviews. 2018;9:CD005528.

7. Kingdon C, Downe S, Betran AP. Women's and communities' views of targeted educational interventions to reduce unnecessary caesarean section: a qualitative evidence synthesis. Reprod Health. 2018;15(1):130.

8. Nsangi A, Semakula D, Oxman AD, Austvoll-Dahlgren A, Oxman M, Rosenbaum S, et al. Effects of the Informed Health Choices primary school intervention on the ability of children in Uganda to assess the reliability of claims about treatment effects: a cluster-randomised controlled trial. The Lancet. 2017;390(10092):374-88.

9. Brownlee S, Chalkidou K, Doust J, Elshaug AG, Glasziou P, Heath I, et al. Evidence for overuse of medical services around the world. The Lancet. 2017;390(10090):156-68.

## **Focus group discussion guide for women**

*Interviewer: The next section of this focus group is about the type of health education that you would like to receive during antenatal care. I would like to ask you some questions about what you think about different topics of health education to be discussed during antenatal care.*

1. For your current pregnancy, do you have a preferred method to give birth? For example, to have a vaginal birth or caesarean section?
   1. *Probe:* How did you form this preference?
2. During antenatal care visits, do you think that women discuss whether they have a preference for vaginal birth or caesarean section with their healthcare provider?
   1. If women do not discuss their preferences about how they prefer to give birth with their provider, do you think that they would have liked to discuss this? Why or why not?
3. During antenatal care visits, what type of information do you think women would want to learn vaginal birth and caesarean section?
   1. Would they want to have information about:
      1. What to expect during a vaginal birth? Why or why not?
      2. Potential harms of vaginal birth? Why or why not?
      3. Benefits of vaginal birth? Why or why not?
      4. Relaxation techniques to cope with labour pain during vaginal birth? Why or why not?
      5. Knowing what to expect after you give birth? Why or why not?
   2. Would they want to have information about:
      1. What to expect during a caesarean section? Why or why not?
      2. Potential harms of caesarean section? Why or why not?
      3. Benefits of caesarean section? Why or why not?
      4. Techniques to cope with recovery after caesarean birth? Why or why not?
   3. Is there any other information about vaginal birth or caesarean section that you think women would like to learn about?
   4. Would women want the help of healthcare professionals to review and discuss this information? Why or why not?
   5. Would women want to discuss this information with other women, or their families? Why or why not?
   6. *When this information is provided to women, it can be presented in two ways. The first way is that it is an only educational tool, designed to improve women’s knowledge. The second way is as a decision tool (e.g. pamphlets, brochure, videos, mobile or online materials), designed to help women to make decisions about whether they prefer vaginal or caesarean birth.* Do you think that women would want this information presented as a decision tool to help them to decide if they prefer vaginal or caesarean birth? Why or why not?
   7. Would women want this information structured in a way just to provide information, for example, NOT to help them make a decision? Why or why not?
4. At what point during pregnancy do you think women would want to receive this information about vaginal birth and caesarean section? Why?
5. How often do you think women would want to receive information about vaginal birth and caesarean section?
   1. *Probe:* Once? More than once?
6. How do you think women would want to receive this information?
   1. *Probe:* Would they want to receive this information verbally, from a healthcare provider? Why or why not?
   2. *Probe:* Would they want to receive this information in a pamphlet or brochure? Why or why not?
   3. *Probe:* Would they want to receive this information using a computer or a mobile phone application? Why or why not?
7. Do you have any other comments or feedback on information that women may like to receive about vaginal birth and caesarean section?

*Interviewer: The next section of this focus group is about breathing and relaxation techniques, which could be taught during antenatal care sessions. I would like to ask you some questions about what you think about breathing and relaxation sessions.*

1. During antenatal care visits, do you think that women discuss with their healthcare providers breathing and relaxation techniques to help cope with labour pain?
   1. How do you think that women discuss this with their healthcare provider?
   2. If women do not discuss breathing and relaxation techniques with their provider, do you think that they would have liked to discuss this? Why or why not?
2. How do you think that training women on breathing and relaxation techniques during antenatal care may help during their labour and childbirth?
3. *Training sessions on breathing and relaxation techniques could be included during antenatal care visits and could be led by a trained nurse or midwife.*
   1. Do you think that women would be interested in training on breathing and relaxation techniques during antenatal care visits? Why or why not?
   2. At what point during pregnancy do you think women would like training on breathing and relaxation techniques?
      1. Why do you think this is the most appropriate timing?
   3. Let us assume that each training session lasts 1 to 2 hours. How many training sessions do you think would be appropriate?
      1. Why do you think this is the most appropriate number of sessions?
   4. Would women want to have their husbands, boyfriends, or another person with them during the training sessions? Why or why not?
4. How do you think that women would like to receive information about breathing and relaxation techniques?
   1. *Probe:* Would they want to receive this information through an interactive training session? Why or why not?
   2. *Probe:* Would they want to receive this information in a pamphlet or brochure? Why or why not?
   3. *Probe:* Would they want to receive this information through an audio or video recording, such as a short video series or podcast? Why or why not?
5. What do you think are some of the challenges to implementing breathing and relaxation techniques for women during antenatal care?
6. Do you have any other comments or feedback on breathing and relaxation techniques for women?

## **Interview guide for providers and administrators**

*Interviewer: The next section of this interview is about the type of health education about mode of delivery that you think women would like to receive during antenatal care. I would like to ask you some questions about what you think about different topics of health education to be discussed during antenatal care.*

1. During antenatal care, do you discuss with women whether they have a preference for vaginal birth or caesarean section?
   1. *IF YES, probe:* What do you discuss with the women?
   2. *IF NO, probe:* Do you think that discussing their preferences for vaginal birth or caesarean section could be helpful? Why or why not?
2. What information do you think could be included in prenatal education about vaginal birth and caesarean section?
   1. Do you think women would want to have information about:
      1. What to expect during a vaginal birth? Why or why not?
      2. Risks of vaginal birth? Why or why not?
      3. Benefits of vaginal birth? Why or why not?
      4. Relaxation techniques to cope with labour pain during vaginal birth? Why or why not?
   2. Do you think women would want to have information about:
      1. What to expect during a caesarean section? Why or why not?
      2. Risks of caesarean section? Why or why not?
      3. Benefits of caesarean section? Why or why not?
      4. Techniques to cope with recovery after caesarean birth? Why or why not?
   3. Is there any other information about vaginal birth or caesarean section that you think women would like to learn about?
   4. How much detail do you think should be provided about each of these topics?
   5. Do you think it would be helpful to have this information structured in a way that helps women to make a decision about whether they prefer a vaginal birth or caesarean section? Why or why not?
   6. Do you think it would be helpful to have this information structured in a way just to provide information, for example, not to help her make a decision? Why or why not?
3. At what point during a woman’s pregnancy do you think they should receive this information about mode of delivery? Why?
4. How often do you think women should receive this information about vaginal birth and caesarean section?
   1. *Probe:* Once? More than once?
5. How would do you think women should receive this information?
   1. *Probe:* Should she receive this information verbally, from her healthcare provider? Why or why not?
   2. *Probe:* Should she receive this information in a pamphlet or brochure? Why or why not?
   3. *Probe:* Should she receive this information using a computer or a mobile phone application? Why or why not?
6. Do you have any other comments or feedback on information that women should receive about mode of delivery?

*Interviewer: The next section of this interview is about breathing and relaxation techniques for women, which could be taught during antenatal care sessions. I would like to ask you some questions about what you think about breathing and relaxation sessions.*

1. During antenatal care visits, do you think that healthcare providers discuss with women breathing and relaxation techniques to help cope with labour pain?
   1. How do you think that healthcare providers discuss this with women?
   2. If healthcare providers do not discuss breathing and relaxation techniques with women, do you think that women would have liked to discuss this? Why or why not?
2. How do you think that training women on breathing and relaxation techniques during antenatal care may help during their labour and childbirth?
   1. As a healthcare provider, how do you think that training women on breathing and relaxation techniques might help you to provide better care for women during childbirth?
3. *Training sessions on breathing and relaxation techniques could be included during antenatal care visits, and could be led by a trained nurse or midwife.*
   1. Do you think that women would be interested in training on breathing and relaxation techniques during antenatal care visits? Why or why not?
   2. At what point during pregnancy do you think women would like training on breathing and relaxation techniques?
      1. Why do you think this is the most appropriate timing?
   3. Let us assume that each training session lasts 1 to 2 hours. How many training sessions do you think would be appropriate?
      1. Why do you think this is the most appropriate number of sessions?
   4. Would women want to have their husbands, boyfriends, or another person with them during the training sessions? Why or why not?
4. How do you think that women would like to receive information about breathing and relaxation techniques?
   1. *Probe:* Would they want to receive this information through an interactive training session? Why or why not?
   2. *Probe:* Would they want to receive this information in a pamphlet or brochure? Why or why not?
   3. *Probe:* Would they want to receive this information through an audio or video recording, such as a short video series or podcast? Why or why not?
5. What do you think are some of the challenges to implementing breathing and relaxation techniques for women during antenatal care?
6. Do you have any other comments or feedback on breathing and relaxation techniques for women?
