# Supplementary material for: Optimising the use of caesarean section: a generic formative research protocol for implementation preparation
Source: Reprod Health. 2019 Nov 19;16:170. doi: 10.1186/s12978-019-0827-1 (PMC6862737; doi:10.1186/s12978-019-0827-1)
Supplement: Supplementary file 6 — Additional file 6. Qualitative module 2: Decision-aids for mode of birth. [file 12978_2019_827_MOESM6_ESM.docx]

# **
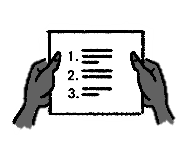
Qualitative module 2: Decision-aids for mode of birth**

## **Overview of intervention**

### *Background*

Shared models of decision-making regarding the mode of delivery between pregnant women and their healthcare providers are increasingly preferred, compared to obstetrician-dominated decisions (1). For pregnant women with a previous caesarean section, there may not be a single best choice about the mode of delivery for their current pregnancy. Decision-aids are an intervention that can be used to present evidence about potential benefits and harms for different treatment options, and can be used to help pregnant women make deliberated and specific choices (2). Decision-aids are designed to supplement (not replace) regular counselling and discussions with healthcare providers (2). They provide detailed, specific and personal options and outcomes in order to prepare women to make the decision about the mode of delivery (2). Informed decision-making around the optimal mode of delivery for women with previous caesarean section requires consideration of the benefits and risks of repeat caesarean section, trial of labour, vaginal birth after caesarean section and the woman’s perspectives and preferences for her childbirth experience (1). In this case, decision-aids can help to provide comprehensive, balanced, and unbiased information, which may help to decrease anxiety about decision-making (2). However, there are no published randomized trials on the effect of decision aids on women without a previous caesarean section.

### *Supporting evidence*

Three randomised trials conducted in the United Kingdom (1), Australia (3), and United States of America (4) found that decision-support tools improved women’s knowledge and reduced decisional conflict about mode of delivery options, but had variable effects on their uptake of trial of labour or vaginal birth after caesarean section. This evidence is aligned with the findings from a Cochrane review that explored the effects of decision aids on people facing health treatment or screening decisions (2). A qualitative evidence synthesis suggests that women welcome new information and learning about childbirth which can mediate pregnant women’s concerns about risks. Women perceive educational interventions and decision-aids tools as a “starting point”, a springboard for seeking more information and for a more meaningful dialogue with health professionals (5).

## **Theory of change**

Decision-aids can be used before, during or after a clinical contact, in order to help women to become more informed and active in their care (2). If women are provided with the decision-aids before a clinical contact, they may have more time to absorb the information and be more prepared to discuss the options (2). Shared decision-making about mode of delivery refers to healthcare providers and women making decisions together about women’s childbirth experiences, and is considered to be at the core of woman-centred care (2). Standardised decision-aids may also reduce the likelihood of clinician preferences dictating a woman’s care pathway (2). Decision-aids may therefore assist women and providers to make decisions that are grounded in women’s values and preferences and account for risks and benefits of different modes of birth (2).

* * *

## **Participants for qualitative research**

| **Data collection methods and participants** | | |
| --- | --- | --- |
| Population | In-depth interview (IDI) | Focus group discussion (FGD) |
| Women |  | 🗸 |
| Healthcare providers  (midwives/nurses, doctors) | 🗸 |  |
| Healthcare administrators  (matron-in-charge, medical director) | 🗸 |  |

| **Population of women** | | |
| --- | --- | --- |
| Nulliparous | Multiparous with previous CS | Multiparous without previous CS |
| 🗸 | 🗸 | 🗸 |

## **Resources and estimated time required to complete this module**

- Trained research assistants
- Audio recorders and notebooks for field notes
- Informed consent forms
- Private room for interviews and focus group
- Focus group discussions with women: 30-45 minutes
- Interviews with healthcare providers and administrators: 10-15 minutes

| *Guiding principles* Interventions to promote group therapy for women with a fear of childbirth should be based on the following guiding principles:   1. **Ensuring autonomy, agency and choice**: All women have the basic right to information to make informed choices about their mode of delivery. They should be provided with the information, education and means to make and implement these choices. 2. **Community participation**: Participatory approaches should be used to assess the needs of needs of women and girls, to ensure community ownership and engagement in developing and implementing sustainable solutions. 3. **Human rights**: Human rights, including those of women, girls and children, must be respected, protected and fulfilled in line with international human rights norms and standards, including the right to the highest attainable standard of health. 4. **Responsiveness of health systems**: Health systems need to be organized and managed so that they facilitate respect, protection and fulfilment of women’s sexual and reproductive health and rights. Provisions should be made to ensure privacy and confidentiality, and respect for women’s decision-making on mode of delivery. All involved in the care-giving process also need to understand their corresponding obligations and relevant standards of conduct. 5. The degree of involvement in the decision-making process about mode of birth may vary among women. Some women may be highly motivated to be involved, while others may be uncertain of their role or want a healthcare professional to make the decision for them. These differences need to be considered, identified and respected. |
| --- |

**References**

1. Montgomery AA, Emmett CL, Fahey T, Jones C, Ricketts I, Patel RR, et al. Two decision aids for mode of delivery among women with previous caesarean section: randomised controlled trial. BMJ. 2007;334(7607):1305.

2. Stacey D, Légaré F, Lewis K, Barry MJ, Bennett CL, Eden KB, et al. Decision aids for people facing health treatment or screening decisions. Cochrane Database of Systematic Reviews. 2017(4).

3. Shorten A, Shorten B, Keogh J, West S, Morris J. Making Choices for Childbirth: A Randomized Controlled Trial of a Decision‐aid for Informed Birth after Cesarean. Birth. 2005;32(4):252-61.

4. Eden KB, Perrin NA, Vesco KK, Guise J-M. A Randomized Comparative Trial of Two Decision Tools for Pregnant Women with Prior Cesarean. Journal of Obstetric, Gynecologic & Neonatal Nursing. 2014;43(5):568-79.

5. Kingdon C, Downe S, Betran AP. Women’s and communities’ views of targeted educational interventions to reduce unnecessary caesarean section: a qualitative evidence synthesis. Reproductive Health. 2018;15(1):130.

## **Focus group discussion guide for women**

*Interviewer: The next section of this discussion is about using decision-tools to help you to understand your choices about how you will give birth to your baby. By this, we mean whether you will try to have a vaginal birth or a caesarean section. These decision-tools would provide you with information about potential benefits and harms of the different options which you could assess according to your own preferences. They would be in addition to any regular counselling or discussions with your healthcare providers. I would like to ask you some questions about what you think about these decision-tools.*

1. Could you tell me what you know about the different ways that women can give birth?
   1. How do women in your community learn about these options?
   2. What type of information do you think women in your community would like to know about their options for how to give birth to their baby?
      1. *Probe:* Potential harms of different methods, benefits of different methods, personal preferences
2. *A decision-tool (such as a computer, tablet or a smart phone) could provide descriptions of the health outcomes associated with planned vaginal birth, planned caesarean section, and emergency caesarean section. They can also ask you questions about your own values and preferences for possible outcomes. Once you provide this information, the decision-tool can produce a recommended “preferred option”, based on your preferences and priorities. You could then bring this to your healthcare provider to discuss in more detail.* What do you think about this type of decision-tool?
   1. Does this description of a decision-tool sound like something that might be useful to women in your community? Why or why not?
   2. How might this decision-tool help women to make a decision about how they will give birth?
   3. What do you think are some of the benefits of women using a decision-tool to decide about how they will give birth?
   4. What do you think are some of the challenges of women using a decision-tool to decide about how they will give birth?
      1. Do you think that using decision-tools may cause anxiety or stress for some women? Why or why not?
   5. At what point during a woman’s pregnancy would it be most helpful to have access to this type of decision-tool? Why?
   6. How do you think women would use the results of the decision tool, or the “preferred option”, to discuss their options with different people?
      1. *Probe:* To discuss with their healthcare provider?
      2. *Probe:* To discuss with their partner or family?
   7. Do you think that you would use this type of decision-tool? Why or why not?
3. *These types of decision-tools can come in different formats. For example, on a computer, a tablet, or a smart phone.* What format do you think would be most helpful to women in your community? Why?
4. Do you have any other comments or feedback about decision-tools to help women choose how to give birth?

## **Interview guide for providers and administrators**

*The next section of this interview is about using decision-tools (such as a computer, tablet or a smart phone) to help pregnant women with previous caesarean sections to understand their choices about mode of delivery. By this, we mean whether the woman will have planned vaginal birth, trial of labour, or a caesarean section. These decision-tools would provide them with information about potential benefits and harms of the different options. They would be in addition to any regular counselling or discussions with healthcare providers. I would like to ask you some questions about what you think about these decision-tools.*

1. In your practice, how do you communicate to women with a previous caesarean section about the different options for mode of delivery?
   1. What type of resources do you have to help you to have these discussions?
2. In your practice, what have pregnant women told you about how they access information about their options for mode of delivery?
   1. What do you think about these information resources?
3. In your opinion, do you think that women have sufficient knowledge about their options for mode of delivery? Why or why not?
4. What type of information do you think that women need to inform their preferences and decisions about their mode of delivery?
   1. *Probe:* Risks of different methods, benefits of different methods, personal preferences
5. Do you think that groups of women may have different needs for information about mode of delivery, for example, women with a previous caesarean section?
   1. *Probe*: What type of information do you think that these women might need?
6. *A decision-tool could provide descriptions of the health outcomes associated with planned vaginal birth, planned caesarean section, and emergency caesarean section. They can also ask questions about a woman’s values and preferences for possible outcomes. Once this information is provided, the decision-tool can produce a recommended “preferred option”, based on a woman’s preferences. The woman could then bring this to her healthcare provider to discuss in more detail.* What do you think about this type of decision-tool?
   1. Does this description of a decision-tool sound like something that might be useful to you? Why or why not?
   2. How might this decision-tool help you to discuss options about how a woman will give birth?
   3. What do you think are some of the benefits of using a decision-tool to help decide about how a woman will give birth?
      1. *Probe:* to you as a provider?
      2. *Probe:* to the woman
   4. What do you think are some of the challenges of using a decision-tool to help decide about how a woman will give birth?
      1. *Probe:* to you as a provider?
      2. *Probe:* to the woman
   5. At what point during a woman’s pregnancy would it be most helpful for her to have access to this type of decision-tool? Why?
   6. How might you use the results of the decision tool, or the woman’s “preferred option”, to discuss her options for mode of delivery?
   7. Do you think that you would recommend that women use this type of decision-tool? Why or why not?
7. *These types of decision-tools can come in different formats. For example, on a computer, a tablet, or a smart phone.* What format do you think would be most helpful? Why?
8. Do you have any other comments or feedback about decision-tools to help you discuss options about how a woman will give birth?
