# Supplementary material for: Optimising the use of caesarean section: a generic formative research protocol for implementation preparation
Source: Reprod Health. 2019 Nov 19;16:170. doi: 10.1186/s12978-019-0827-1 (PMC6862737; doi:10.1186/s12978-019-0827-1)
Supplement: Supplementary file 7 — Additional file 7. Qualitative module 3: Psychosocial support for women with fear of childbirth. [file 12978_2019_827_MOESM7_ESM.docx]

#
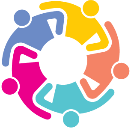
**Qualitative module 3: Psychosocial support for women with fear of childbirth**

## **Overview of intervention**

### *Background*

Research has suggested that fear of labour and childbirth may affect between 5 to 20 percent of all women [1], and may be expressed as physical complaints, nightmares, and difficulty concentrating [2]. There are several factors associated with a fear of childbirth, including nulliparity, young maternal age, pre-existing psychological problems, lack of social support, and history of abuse or previous childbirth complications [1]. Fear of labour and childbirth has been shown to be a contributing factor to maternal request for caesarean section [3-5]. For example, a study conducted in Norway found that women who feared childbirth were more than four times as likely to have a preference for caesarean section, and more than twice as likely to receive a caesarean section, compared to women who did not fear childbirth [5]. Identifying and managing maternal fear and anxiety may therefore have an impact on reducing maternal request for caesarean section, in addition to improving maternal and infant morbidity [2].

### *Supporting evidence*

Psychoeducation for women with a fear of childbirth may play a role in reducing maternal request for caesarean section, however limited research has been conducted on the most effective treatment. The Cochrane review on non-clinical interventions to reduce unnecessary caesarean sections identified two randomised controlled trials that demonstrated little or no difference in caesarean section rates between standard maternity care and providing psychoeducation or intensive group therapy (cognitive behavioural therapy and childbirth psychotherapy) for women with fear of childbirth [2, 6, 7]. Psychoeducation was found to increase rates of vaginal births. The certainty of evidence was low [8].

Based on this evidence, psychoeducation group therapy for women with fear of childbirth is recommended by WHO *in the context of targeted monitoring and evaluation*.

## **Theory of change**

Research has shown that fear of childbirth is associated with women’s preferences for caesarean section [1]. Treating anxiety and fear of childbirth may reduce perceived fear, anxiety and stress, and help a woman have a more positive pregnancy experience [9]. A reduction in women’s fear of childbirth may therefore result in a reduction of maternal request for caesarean section, which may have an overall impact on elective caesarean section rates.

## **Participants for qualitative research**

| **Data collection methods and participants** | | |
| --- | --- | --- |
| Population | In-depth interview (IDI) | Focus group discussion (FGD) |
| Women |  | 🗸 |
| Healthcare providers  (midwives, nurses, doctors) | 🗸 |  |
| Healthcare administrators  (matron-in-charge, medical director) | 🗸 |  |

| **Population of women** | | |
| --- | --- | --- |
| Nulliparous | Multiparous with previous CS | Multiparous without previous CS |
| 🗸 | 🗸 | 🗸 |

## **Resources and estimated time required to complete this module**

- Trained research assistants
- Audio recorders and notebooks for field notes
- Informed consent forms
- Private room for focus group
- Focus group discussions with women: 30 minutes
- Interviews with healthcare providers and administrators: 10-15 minutes

| *Guiding principles* Group therapy interventions for women with a fear of childbirth should be based on the following guiding principles:   1. **Ensuring autonomy, agency and choice**: All women have the basic right to decide freely whether to participate in group therapy or to request a caesarean section. They should be provided with the information, education and means to make and implement these choices. 2. **Community participation**: Participatory approaches should be used to assess the needs of women and girls, to ensure community ownership and engagement in developing and implementing sustainable solutions. 3. **Human rights**: Human rights, including those of women, girls and children, must be respected, protected and fulfilled in line with international human rights norms and standards, including the right to the highest attainable standard of health. 4. **Responsiveness of health systems**: Health systems need to be organized and managed so that they facilitate respect, protection and fulfilment of women’s sexual and reproductive health and rights. Provisions should be made to ensure privacy and confidentiality, and respect for women’s decision-making on whether to attend group therapy or to request a caesarean section. All involved in the care-giving process also need to understand their corresponding obligations and relevant standards of conduct. |
| --- |

**References**

1. Adams SS, Eberhard-Gran M, Eskild A. Fear of childbirth and duration of labour: a study of 2206 women with intended vaginal delivery. BJOG. 2012;119(10):1238-46.

2. Saisto T, Salmela-Aro K, Nurmi J-E, et al. A randomized controlled trial of intervention in fear of childbirth. Obstetrics & Gynecology. 2001;98(5, Part 1):820-6.

3. Nieminen K, Stephansson O, Ryding EL. Women's fear of childbirth and preference for cesarean section--a cross-sectional study at various stages of pregnancy in Sweden. Acta Obstet Gynecol Scand. 2009;88(7):807-13.

4. Saisto T, Halmesmaki E. Fear of childbirth: a neglected dilemma. Acta Obstet Gynecol Scand. 2003;82(3):201-8.

5. Størksen HT, Garthus-Niegel S, Adams SS, et al. Fear of childbirth and elective caesarean section: a population-based study. BMC Pregnancy and Childbirth. 2015;15:221.

6. Chen I, Opiyo N, Tavender E, et al. Non-clinical interventions for reducing unnecessary caesarean section. The Cochrane database of systematic reviews. 2018;9:CD005528.

7. Rouhe H, Salmela-Aro K, Toivanen R, et al. Obstetric outcome after intervention for severe fear of childbirth in nulliparous women - randomised trial. BJOG. 2013;120(1):75-84.

8. World Health Organization. WHO recommendations on non-clinical interventions to reduce unnecessary caesarean sections. Geneva, Switzerland: World Health Organization; 2018.

9. Saisto T, Toivanen R, Salmela-Aro K, et al. Therapeutic group psychoeducation and relaxation in treating fear of childbirth. Acta Obstet Gynecol Scand. 2006;85(11):1315-9.

## **Focus group discussion guide for women**

*Interviewer: The next part of the discussion is about interventions to help women who have a fear of labour and childbirth. Women who have a fear of childbirth may be more likely to request a caesarean section even if it would not be really necessary from the medical perspective. Some research has shown that group therapy during pregnancy may help to reduce women’s fear of labour and childbirth. I would like to ask you some questions about what you think about group therapy to help women who have a fear of childbirth.*

1. Have you heard about women in your community having a fear of childbirth?
   1. What have you heard about this fear?
   2. Have you heard about women who have a fear of childbirth requesting to have a caesarean section? Please explain.
2. If women in your community have a fear of childbirth, what type of support do they have access to?
   1. Do you think this support is adequate to help them cope? Why or why not?
3. *Interviewer: Group therapy for women with a fear of childbirth may help to reduce fear and provide women with coping strategies during pregnancy and labour. Group therapy would typically include developing group therapeutic skills, such as relaxation skills, normalisation of fears, and information about what to expect during labour and birth.*
   1. How do you think group therapy might help women in your community who have a fear of childbirth?
   2. Can you think of any reasons why women in your community may not benefit from group therapy?
   3. Do you think women in your community would be accepting of group therapy for fear of childbirth? Why or why not?
   4. Do you think that group therapy for women with a fear of childbirth would help doctors and nurses to provide better care? Why or why not?
   5. What do you think are some of the challenges to implementing group therapy in your community?
   6. What are some of the things that may help to implement group therapy in your community?

## **Interview guide for providers and administrators**

*Interviewer: The next part of the study is about interventions to help women who have a fear of labour and childbirth. Fear of childbirth has been shown to be positively associated with maternal request for caesarean section. Some research has shown that group therapy during pregnancy may help to reduce women’s fear of labour and childbirth. I would like to ask you some questions about what you think about group therapy to help women who have a fear of childbirth.*

1. In your health facility, have you noticed that some women request a caesarean section, because they are afraid of labour or childbirth?
   1. *If yes, probe*: Can you tell me about a time when this happened?
      1. *Probe:* Why was the woman afraid?
      2. *Probe:* What did the healthcare providers do to support the woman?
      3. *Probe:* What else do you think could have been done to support the woman?
   2. *If no, probe:* Can you tell me about a time when you cared for a woman who was afraid of labour and childbirth?
      1. *Probe:* Why was the woman afraid?
      2. *Probe:* What did the healthcare providers do to support the woman?
      3. *Probe:* What else do you think could have been done to support the woman?
2. *Group therapy has been suggested as an intervention that may help to reduce women’s fears of childbirth. An intervention of group therapy may involve several group sessions throughout pregnancy that discuss a focused topic and relaxation exercises. For example, the relaxation exercise may include guiding the participants through stages of imaginary birth in a relaxed state of mind with positive, calming and supportive suggestions. The topics covered may include information about fear and anxiety, fear of childbirth, normalization of individual reactions, stages of labour, hospital routines, the birth process, and pain relief. These sessions may be led by psychologists or other social workers.* What do you think about the idea of group therapy to help women who have a fear of childbirth?
   1. How do you think that group therapy may help women who have a fear of childbirth?
   2. How do you think that group therapy may help healthcare providers to provide care to women who have a fear of childbirth?
   3. Can you think of any reasons that group therapy may not be helpful to women who have a fear of childbirth?
3. *Now I would like to ask you to imagine a hypothetical situation where the health facility management has decided to implement group therapy for women who are afraid of childbirth.* How do you think that you could integrate group therapy into antenatal care in your current health facility?
   1. Do you think that this would be acceptable to the other healthcare providers working in your facility? Why or why not?
   2. Do you think that this would be acceptable to pregnant women who attend your health facility? Why or why not?
   3. What are some of the challenges to implementing group therapy in your health facility?
   4. What are some of the things that may help to implement group therapy in your health facility?
   5. What additional resources do you think your facility would need in order to implement group therapy?
   6. Who do you think would be the best people to provide a group therapy intervention?
   7. How do you think healthcare providers could best identify women who have a fear of childbirth?
