# Supplementary material for: Optimising the use of caesarean section: a generic formative research protocol for implementation preparation
Source: Reprod Health. 2019 Nov 19;16:170. doi: 10.1186/s12978-019-0827-1 (PMC6862737; doi:10.1186/s12978-019-0827-1)
Supplement: Supplementary file 8 — Additional file 8. Qualitative module 4: Labour companionship. [file 12978_2019_827_MOESM8_ESM.docx]

# **
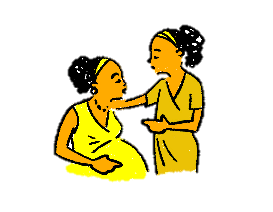
Qualitative module 4: Labour companionship**

## **Overview of intervention**

### *Background*

Research shows that women value and benefit from the presence of a support person during labour and childbirth [1]. The person providing support may be someone from the woman’s social network (such as a partner, family member or friend), hospital staff (such as nurses or midwives), or women who were not hospital staff and had no personal relationship to the woman in labour (such as a doula) [1]. The type of support provided may include emotional support (continuous presence, reassurance and praise), and information about labour progress [1]. It may also include advice about coping techniques, comfort measures (such as a comforting touch, massage, encouraging mobility, and promoting adequate fluid intake and output), and speaking up when needed on behalf of the woman [1]. When women do not receive continuous support throughout labour and childbirth, there are concerns that women’s experiences of labour and birth may become dehumanised [1].

### *Supporting evidence*

A Cochrane intervention review synthesized evidence on continuous support from 26 studies conducted across 17 countries [1]. Continuous support was provided by either a companion of the woman’s choice from her social network (such as a partner, family member or friend), hospital staff (such as nurses or midwives), or women who were not hospital staff and had no personal relationship to the woman in labour (such as a doula). Women who received continuous support may be more likely to give birth “spontaneously” (vaginally, without the use of forceps, vacuum, or caesarean), and to be satisfied with their birth [1]. They were also less likely to use pain medication or have caesarean birth [1].

Based on this evidence, labour companionship is recommended in three WHO guidelines [2-4].

## **Theory of change**

Two complimentary theoretical explanations have been proposed to explain the effects of companionship [1]. Both theories hypothesise that support during labour and childbirth enhances the woman’s physiology and her feelings of control and competence, thus reducing reliance on medical interventions [1]. The first explanation hypothesises that modern obstetric care frequently subjects women to institutional routines, high rates of intervention, unfamiliar personnel, lack of privacy and other conditions that a women may experience as harsh [1]. The provision of support from a companion may help to buffer the woman from these stressors. The second explanation hypothesises that support during labour enhances fetopelvic relationships (such as by encouraging mobility and effective use of gravity), and decreases the woman’s stress responses [1]. Support from a companion throughout labour and birth may thus reduce a woman’s anxiety and fear.

## **Participants for qualitative research**

| **Data collection methods and participants** | | |
| --- | --- | --- |
| Population | In-depth interview (IDI) | Focus group discussion (FGD) |
| Women |  | 🗸  Mixed FGDs with multiparous and nulliparous women during antenatal care |
| Healthcare providers  (midwives, nurses, doctors) | 🗸 |  |
| Healthcare administrators  (matron-in-charge, medical director) | 🗸 |  |

## **Resources and estimated time required to complete this module**

- Trained research assistants
- Audio recorders and notebooks for field notes
- Informed consent forms
- Private room for interview/focus group
- Interviews with women: 15 minutes

Interviews with healthcare providers and administrators: 20 minutes

| *Guiding principles* Intervention to promote and accommodate companions of choice for women during labour and childbirth should be based on the following guiding principles [5]:   1. **Ensuring autonomy, agency and choice**: All women have the basic right to decide freely whether to have a childbirth companion, and whom to choose. They should be provided with the information, education and means to make and implement these choices. 2. **Community participation**: Participatory approaches should be used to assess the needs of women and girls, to ensure community ownership and engagement in developing and implementing sustainable solutions. 3. **Human rights**: Human rights, including those of women, girls and children, must be respected, protected and fulfilled in line with international human rights norms and standards, including the right to the highest attainable standard of health. 4. **Responsiveness of health systems**: Health systems need to be organized and managed so that they facilitate respect, protection and fulfilment of women’s sexual and reproductive health and rights. Provisions should be made to ensure privacy and confidentiality, and respect for women’s decision-making on whether to have a childbirth companion, and whom to choose. All involved in the care-giving process also need to understand their corresponding obligations and relevant standards of conduct. |
| --- |

**References**

1. Bohren MA, Hofmeyr GJ, Sakala C, Fukuzawa RK, Cuthbert A. Continuous support for women during childbirth. The Cochrane database of systematic reviews. 2017;7:Cd003766.

2. World Health Organization. WHO recommendations on health promotion interventions for maternal and newborn health. Geneva, Switzerland; 2015.

3. World Health Organization. WHO Recommendations: Intrapartum care for a postive childbirth experience. Geneva, Switzerland: World Health Organization; 2018.

4. World Health Organization. WHO recommendations for augmentation of labour. Geneva, Switzerland: World Health Organization; 2014.

5. World Health Organization. Companion of choice during labour and childbirth for improved quality of care. Geneva, Switzerland; 2016.

## **Focus group discussion guide for women**

*Interviewer: The next section of this interview is about the type of support that you would like to receive during your childbirth in a health facility. In some settings, a “labour companion” can provide this type of support. A labour companion is a person of the woman’s choice, such as her husband, her mother, her sister, a friend, or a doula, who stays with the woman throughout the duration of her labour and childbirth. This person provides support to the woman, such as emotional support, which means that the person praises and reassures the woman. This person may also provide information about how the woman is progressing throughout labour, techniques to cope with pain, and comfort measures such as holding her hand, massaging her back, and helping her walk. I would like to ask you some questions about what you think about support from a companion during childbirth.*

1. What type of support do you think women need during labour and childbirth?
2. Have you heard of any women who receive support from a labour companion? Please describe.

*As I mentioned, a labour companion is a person of the woman’s choice who can help to provide emotional support to the woman throughout the duration of her labour and childbirth.*

1. Do you think that women in your community would want to have a labour companion with them? Why or why not?
2. If women in your community chose to have a labour companion, who do you think they would prefer as a labour companion? Why?
3. What do you think that women would expect from a labour companion?
4. What types of tasks or responsibilities do you think labour companions could help women with?
5. When would / do you think women would want to have a labour companion with them in the hospital? *(Prompt for different time periods if the woman is unsure, e.g.: on admission to the health facility, throughout labour, during childbirth, after childbirth)*
6. When do you think women should start talking to their labour companion about their role during labour and childbirth? *(Prompt if the woman is unsure: during pregnancy/antenatal care visits, on arrival to the health facility for childbirth)*
7. Do you know if labour companionship is allowed in this hospital?
   1. *If labour companionship is not allowed:* What do you think are the reasons for not allowing a labour companion in this hospital?
8. What changes do you think the hospital could make to make it more comfortable for women to have a labour companion?
9. Do you have any other comments or feedback about labour companionship?

**Interview guide for providers and administrators**

*Interviewer: The next part of the study is about the type of support that women could receive during childbirth in a health facility. In some settings, a “labour companion” can provide this type of support. A labour companion is a person of the woman’s choice, for example her husband, her sister, her mother, her friend, or a doula, who stays with the woman throughout the duration of labour and childbirth. I would like to ask you some questions about what you think about support from a companion during childbirth.*

1. What type of support do you think women need during labour and childbirth?
   1. Do you think that women in your hospital receive this kind of support you have described? Why or why not?
2. What do you know about labour companionship?
   1. What are the benefits of labour companionship?
      1. *Probe*: What are benefits for the woman?
      2. *Probe*: What are benefits for the providers?
   2. Are there any harms of labour companionship?
      1. *Probe*: What are harms for the woman?
      2. *Probe*: What are harms for the providers?
3. Do you know if labour companionship is allowed in this hospital?
   1. *If labour companionship is not allowed:* What do you think are the reasons for not allowing a labour companion in this hospital?
   2. Do you have any previous experience with working in a hospital that offered labour support?
      1. If yes, what was this experience like for you as a provider?
4. How could labour companionship be implemented in your hospital or other hospitals like this?
   1. What would be the main challenges to implementing labour companionship?
   2. Who do you think women would prefer as a labour companion? Why?
   3. As a provider, what are your expectations from a woman’s labour companion?
   4. When would a labour companion be able to be with the woman in the hospital?
   5. What would the role of the labour companion be?
      1. How could the labour companion’s roles be communicated to them?
   6. At what point during the care process should women and providers start talking about labour companionship and the role of the companion?
   7. How could we ensure that the companion is a person of the woman’s choice, and not someone selected for her by someone else?
   8. What changes do you think the hospital could make to make it more comfortable for women to have a labour companion?
   9. If labour companionship is to be implemented in this hospital, what would ensure successful implementation?
      1. What could be done to ensure that labour companionship was sustainable in the long-term?
5. Do you have any other comments or feedback about labour companionship in your health facility?
