# Supplementary material for: Optimising the use of caesarean section: a generic formative research protocol for implementation preparation
Source: Reprod Health. 2019 Nov 19;16:170. doi: 10.1186/s12978-019-0827-1 (PMC6862737; doi:10.1186/s12978-019-0827-1)
Supplement: Supplementary file 9 — Additional file 9. Qualitative module 5: Public dissemination of caesarean section rates at a facility-level. [file 12978_2019_827_MOESM9_ESM.docx]

# **
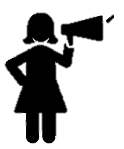
Qualitative module 5: Public dissemination of caesarean section rates at a facility-level**

## **Overview of intervention**

### *Background*

It is increasingly common to publicly release information about health system and health facility performance [1]. This performance data may have an impact on changing the behaviours of healthcare providers, consumers, and professionals that can improve performance and quality of care [1]. Some evidence suggests that publicly releasing performance data may stimulate quality improvement activities at the hospital level [2]. In the context of caesarean section, public dissemination of caesarean section rates may influence the behaviours of both healthcare providers and women, and may promote quality improvement activities within hospitals and more appropriate decision-making for caesarean section.

Note: This intervention specifically relates to public dissemination of caesarean section rates at a *facility-level*, and may need to be adapted if disseminating doctor-specific caesarean section rates would be more appropriate.

### *Supporting evidence*

Across healthcare disciplines, there is inconsistent evidence that the public dissemination of performance data changes behaviours or improves care [1]. There is limited evidence to support or refute the hypothesis that public dissemination of caesarean section rates can reduce caesarean section rates, thus studies evaluating the effects of this intervention are needed [3].

Based on the limited evidence, WHO has called for more research to explore the potential impacts of public dissemination of caesarean section rates.

## **Theory of change**

The impact of the public release of performance data may operate through several different mechanisms. Public dissemination may influence providers to improve their performance, through either a selection or change pathway [1, 4]. In a selection pathway, consumers, patients, and other stakeholders can select hospitals based on the best potential outcomes [1]. While this would not change the quality of care provided at an individual hospital, it may stimulate quality improvement [1]. In a change pathway, the public dissemination may influence providers and hospitals to prioritise quality improvement by changing professional culture, clinical practice, and other structural changes [1].

## **Participants for qualitative research**

| **Data collection methods and participants** | | |
| --- | --- | --- |
| Population | In-depth interview (IDI) | Focus group discussion (FGD) |
| Women |  | 🗸 |
| Healthcare providers  (midwives, nurses, doctors) | 🗸 |  |
| Healthcare administrators  (matron-in-charge, medical director) | 🗸 |  |

| **Population of women** | | |
| --- | --- | --- |
| Nulliparous | Multiparous with previous CS | Multiparous without previous CS |
| 🗸 | 🗸 | 🗸 |

## **Resources and estimated time required to complete this module**

- Trained research assistants
- Audio recorders and notebooks for field notes
- Informed consent forms
- Private room for interview/focus group
- Focus group discussions with women: 20-30 minutes
- Interviews with healthcare providers and administrators: 10-15 minutes

| *Guiding principles*  - Sufficient information should be provided to the general public to improve their understanding of how to interpret the caesarean section rates. - Public dissemination of caesarean section rates should be considered a learning opportunity rather than punitive action. |
| --- |

**References**

1. Ketelaar NA, Faber MJ, Flottorp S, Rygh LH, Deane KH, Eccles MP. Public release of performance data in changing the behaviour of healthcare consumers, professionals or organisations. The Cochrane database of systematic reviews. 2011(11):CD004538.

2. Fung CH, Lim YW, Mattke S, Damberg C, Shekelle PG. Systematic review: the evidence that publishing patient care performance data improves quality of care. Annals of internal medicine. 2008;148(2):111-23.

3. World Health Organization. WHO recommendations on non-clinical interventions to reduce unnecessary caesarean sections. Geneva, Switzerland: World Health Organization; 2018.

4. Berwick DM, James B, Coye MJ. Connections between quality measurement and improvement. Med Care. 2003;41(1 Suppl):I30-8.

**Focus group discussion guide for women**

*Interviewer: The next part of the study is about public dissemination of caesarean section rates. This means that hospital-specific caesarean section rates would be openly available to the public. I would like to ask you some questions about what you think about public dissemination of caesarean section rates. The caesarean section rate is the percentage of women in a specific hospital who give birth by caesarean section. For example, a 50% caesarean section rate means that half of the women giving birth in that hospital have a caesarean section.*

1. Do you think that women in your community would like to know the rate of caesarean sections in a hospital, before deciding to give birth there? Why or why not?
2. Would knowing a hospital’s caesarean section rate influence your decision on where to give birth?
3. Do you think that people will understand how to interpret caesarean section rates? Why or why not?
   1. If you find out that a hospital’s caesarean section rate is 30%, how would you interpret it?
4. Is there any other information or data about the hospital that you think women would like to see in order to better understand a hospital’s caesarean section rate?
5. Do you think that publicly sharing a hospital’s caesarean section rate would be acceptable in your community? Why or why not?
6. What are some of the benefits of publicly sharing a hospital’s caesarean section rate?
7. What are some of the challenges of publicly sharing a hospital’s caesarean section rate?
8. In your opinion, do you think that publicly sharing a hospital’s caesarean section rate would influence the behavior of doctors?
9. In your opinion, do you think that it is a good or bad idea to publicly share a hospital’s caesarean section rate?
10. If the caesarean section rates for all hospitals were to be openly available to the public, what would be the most appropriate format for people to view them?
    1. *Probe:* Website? Press release? Smartphone app? Something else?
11. Do you have any other comments or feedback on the public dissemination of caesarean section rates?

## **Interview guide for providers and administrators**

*Interviewer: The next part of the study is about public dissemination of caesarean section rates. This means that hospital-specific caesarean section rates would be openly available to the public. I would like to ask you some questions about what you think about public dissemination of caesarean section rates.*

1. How would you feel if *hospital-specific* caesarean section rates were disseminated to the public?
2. What are some of the benefits of disseminating *hospital-specific* caesarean section rates to the public?
3. What are some of the challenges of disseminating *hospital-specific* caesarean section rates to the public?
4. Would you be supportive of disseminating *hospital-specific* caesarean section rates to the public? Why or why not?
5. Do you think that disseminating *hospital-specific* caesarean section rates to the public would have an influence on individual providers’ performance or clinical practice? Why or why not?
6. Do you think that disseminating *hospital-specific* caesarean section rates to the public would have an influence on a hospital’s performance or clinical practices? Why or why not?
7. If the Ministry of Health decided that all *hospital-specific* caesarean section rates were to be publicly disseminated, what challenges do you think they would face?
8. If all *hospital-specific* caesarean section rates were to be publicly disseminated, what would be the most appropriate format for dissemination?
   - 1. *Probe:* Website? Press release? Smartphone app? Something else?
9. Do you have any other comments or feedback on the public dissemination of caesarean section rates?
