# Supplementary material for: Optimising the use of caesarean section: a generic formative research protocol for implementation preparation
Source: Reprod Health. 2019 Nov 19;16:170. doi: 10.1186/s12978-019-0827-1 (PMC6862737; doi:10.1186/s12978-019-0827-1)
Supplement: Supplementary file 10 — Additional file 10. Qualitative module 6: Audit and feedback including external review of labour and delivery records and use of Robson classification as a feedback too. [file 12978_2019_827_MOESM10_ESM.docx]

# **Qualitative module 6: Audit and feedback including external review of labour and delivery records and use of Robson classification as a feedback tool**


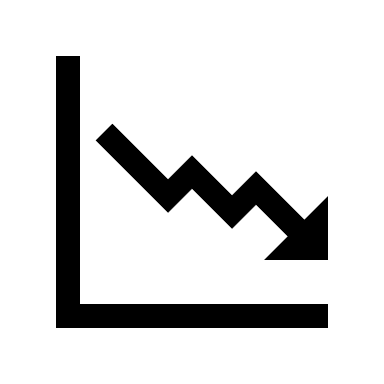

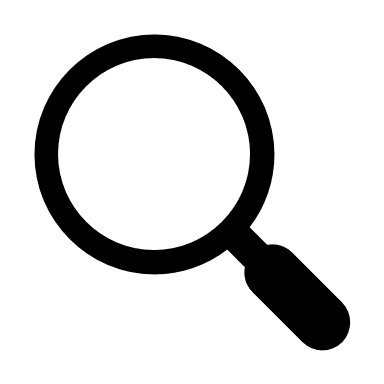


## **Overview of intervention**

### *Background*

The purpose of an audit and feedback process is to encourage individuals and teams to follow professional standards or targets. During an audit and feedback process in healthcare, an individual’s professional practice and/or performance is measured and compared to targets or professional standards (1). The results of this comparison are fed back to the individual by either a colleague, supervisor or third party, in the form of verbal or written communication (1). Typically, this also involves a discussion about areas for improvement, and is often done in conjunction with other interventions such as training, leadership, or quality improvement meetings (1). Feedback may be given once, a fixed number of times, or in a recurring manner. In the context of obstetric care, audit and feedback may be useful to explore:

- Critical incidents such as maternal or neonatal deaths or near miss;
- Indications for caesarean section;
- Decision-to-incision time intervals for caesarean section;
- Case fatality rates for women who received a caesarean section;
- Stillbirth and early neonatal death rates;
- Maternal and fetal outcomes;
- Administration of prophylactic antibiotics;
- Use of the partograph (or other tool to monitor the progression of labour).
- Appropriate management of obstetric complications;
- Decision-making processes for caesarean section;
- Appropriate management of uncomplicated labour; and
- Physician and facility-specific caesarean rates.

External review of labour and delivery records can help to ensure that caesarean sections are performed for clinically valid reasons, and identify priority areas for coaching, training, and support for healthcare providers (2). External review can be used as a teaching tool to help healthcare providers agree on the operationalisation of indications for caesarean section in their context.

Furthermore, to better understand contributing factors for the increasing trend and implement measures to reduce or increase caesarean section as needed, tools are needed to monitor and compare caesarean section rates over time in the same setting and between different settings (3). A WHO-led systematic review (4) concluded that the Robson 10 group classification system (5) was the best classification system to apply internationally due to its simplicity, clinically-relevant data, accountability, replicability, verifiability, and woman-centeredness. The Robson classification system is a classification system for all women giving birth in a specific setting (not only for women giving birth by caesarean section), and prospectively identifies groups of women who are admitted for childbirth. Each of the ten categories are mutually exclusive and totally inclusive, and every woman admitted for childbirth can be immediately classified based on a few obstetrical characteristics that are routinely collected in healthcare facilities worldwide (3). Classifying women using this model allows for comparison and analysis of caesarean section rates within and across the different groups of women, as well as comparisons to other facilities and countries globally in a standardised way (3, 6, 7).

### *Supporting evidence*

Evidence on the effect of audit and feedback and peer review with the objective to reduce unnecessary caesarean sections is available from two cluster-randomised trials from Canada, and three interrupted time series studies from Chile, Iran, and the United States of America (8). High-certainty evidence shows that the implementation of guidelines combined with audit and feedback slightly reduces caesarean section rates (in women with low-risk pregnancies), assisted vaginal delivery, major and minor neonatal morbidity, intrapartum and neonatal deaths, major trauma, and use of invasive mechanical ventilation (8, 9).

Several studies conducted in a range of low- and middle-income countries found that caesarean section record reviews highlighted key areas for improvement in record keeping (2, 10, 11). A cross-sectional study of caesarean section record review conducted in five low- and middle-income countries (Bangladesh, Guinea, Mali, Niger and Uganda) identified key areas to improve record keeping, which served as a practical guide to initiate audit and feedback processes (2). Another study conducted in Afghanistan found that caesarean section record reviews identified key areas for quality improvement such as improving decision-making and documentation around caesarean section deliveries (10). In Ethiopia, caesarean section record reviews identified that clinical management protocols for obstetric and newborn care were insufficient (11).

Based on this evidence, implementation of clinical guidelines combined with audit and feedback is recommended by WHO (8). The use of Robson classification is also recommended by WHO to monitor and compare caesarean section rates over time (6).

## **Theory of change**

Many theories exist to explain how audit and feedback may lead to quality improvement (1), including:

- Changing individuals beliefs and awareness about clinical practice and consequences;
- Changing social norms;
- Improving self-efficacy;
- Directing attention to a specific set of tasks.

Healthcare providers may be motivated to improve their practices if it is identified that their clinical skills or practice are inconsistent with clinical guidelines or their peers (1). Connecting the audit and feedback to clear targets and an action plan is likely to contribute to sustained quality improvement (1). Furthermore, in order to design and implement effective measures to optimise caesarean section rates, tools are needed to monitor and compare caesarean section rates in a specific context over time, and between different contexts. Historically, caesarean section rates have been monitored by calculating the overall percentage of deliveries by caesarean section or by indication for caesarean section (4). However, variations in an “overall caesarean section rate” between different contexts and over time are difficult to interpret and compare. A standardized, reliable, consistent, and action-oriented classification system would help to monitor and compare caesarean section rates at the facility level [3]. The classification system would provide a clear understanding of where, when, why, how, and on whom caesarean sections are being performed, which is a critical step to design and implement effective strategies to reduce or increase the rate of caesarean sections in order to improve maternal and perinatal health (3, 4).

## **Participants for qualitative research**

| **Data collection methods and participants** | | |
| --- | --- | --- |
| Population | In-depth interview (IDI) | Focus group discussion (FGD) |
| Women |  |  |
| Healthcare providers  (midwives, nurses, doctors) | 🗸 |  |
| Healthcare administrators  (matron-in-charge, medical director) | 🗸 |  |

## **Resources and estimated time required to complete this module**

- Trained research assistants
- Audio recorders and notebooks for field notes
- Printed handouts of the Robson classification groups and table for presenting Robson data
- Informed consent forms
- Private room for interview
- Interviews with healthcare providers and administrators: 30-45 minutes

| *Guiding principles* Audits and timely feedback to healthcare professionals involved in the decision-making process are recommended to reduce unnecessary caesarean section. Audit and feedback should be conducted as a teaching and learning activity, and should not be used as a punitive measure. Audit and feedback may be most useful when (1):   1. Health outcomes in the facilities are suboptimal; 2. It is provided regularly; 3. It is provided verbally and in writing; 4. It includes clear targets and an action plan; and 5. The person responsible for audit and feedback is a supervisor or colleague.   The Robson classification system can be used for all women giving birth (not just by caesarean section), thus allowing for comparison and analysis of caesarean section rates within and across different groups of women. WHO expects that using the Robson classification system will help healthcare facilities to [1]:   - Optimise caesarean section use by identifying and analysing the groups of women who most and least contribute to overall caesarean section rates, and by focusing interventions on these specific groups of women who are particularly relevant in each healthcare facility; - Assess the effectiveness of strategies or interventions targeted at optimizing the use of caesarean section; - Assess the quality of care and clinical management practices by analysing outcomes by groups of women; and - Assess the quality of the data collected and raise staff awareness about the importance of this data and its use. |
| --- |

**References**

1. Ivers N, Jamtvedt G, Flottorp S, Young JM, Odgaard-Jensen J, French SD, et al. Audit and feedback: effects on professional practice and healthcare outcomes. The Cochrane database of systematic reviews. 2012;6:CD000259.

2. Landry E, Pett C, Fiorentino R, Ruminjo J, Mattison C. Assessing the quality of record keeping for cesarean deliveries: results from a multicenter retrospective record review in five low-income countries. BMC Pregnancy Childbirth. 2014;14:139.

3. World Health Organization. Robson Classification: Implementation Manual. Geneva, Switzerland; 2017.

4. Torloni MR, Betran AP, Souza JP, Widmer M, Allen T, Gulmezoglu M, et al. Classifications for cesarean section: a systematic review. PLoS One. 2011;6(1):e14566.

5. Robson MS. Classification of caesarean sections. Fetal and Maternal Medicine Review. 2001;12(1):23-39.

6. World Health Organization. WHO Statement on Caesarean Section Rates. Geneva, Switzerland; 2015.

7. Betran AP, Vindevoghel N, Souza JP, Gulmezoglu AM, Torloni MR. A systematic review of the Robson classification for caesarean section: what works, doesn't work and how to improve it. PLoS One. 2014;9(6):e97769.

8. World Health Organization. WHO recommendations on non-clinical interventions to reduce unnecessary caesarean sections. Geneva, Switzerland: World Health Organization; 2018.

9. Chen I, Opiyo N, Tavender E, Mortazhejri S, Rader T, Petkovic J,, Yogasingam S, Taljaard M, Agarwal S, Laopaiboon M, Wasiak J, Khunpradit S, Lumbiganon P, Gruen RL, Betran AP. Non-clinical interventions for reducing unnecessary caesarean section. Cochrane Database of Systematic Reviews. Sep 28;9:CD005528. doi: 10.1002/14651858.CD005528.pub3.10. Kim YM, Tappis H Fau - Zainullah P, Zainullah P Fau - Ansari N, Ansari N Fau - Evans C, Evans C Fau - Bartlett L, Bartlett L Fau - Zaka N, et al. Quality of caesarean delivery services and documentation in first-line referral facilities in Afghanistan: a chart review. (1471-2393 (Electronic)).

11. Fesseha N, Getachew A Fau - Hiluf M, Hiluf M Fau - Gebrehiwot Y, Gebrehiwot Y Fau - Bailey P, Bailey P. A national review of cesarean delivery in Ethiopia. (1879-3479 (Electronic)).

**The 10 groups of the Robson classification** (3)

**
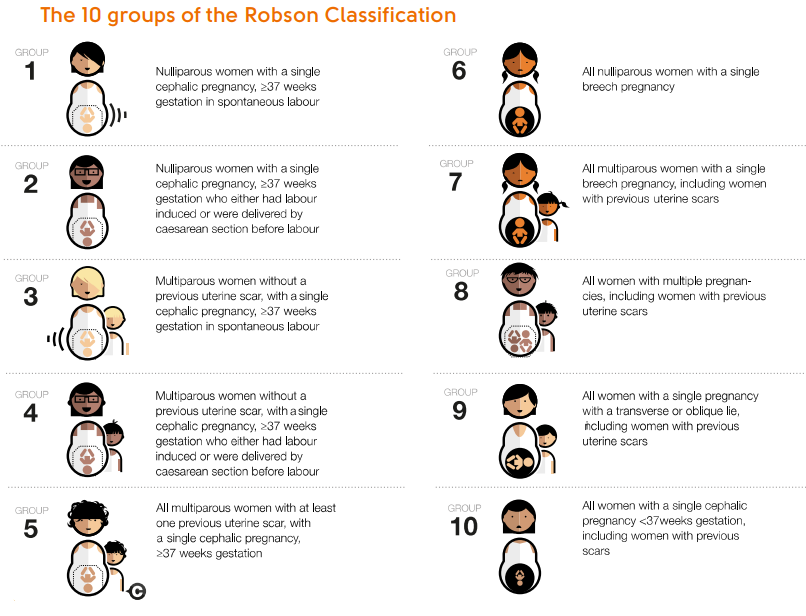
**

**The Robson classification report table** (3)

**
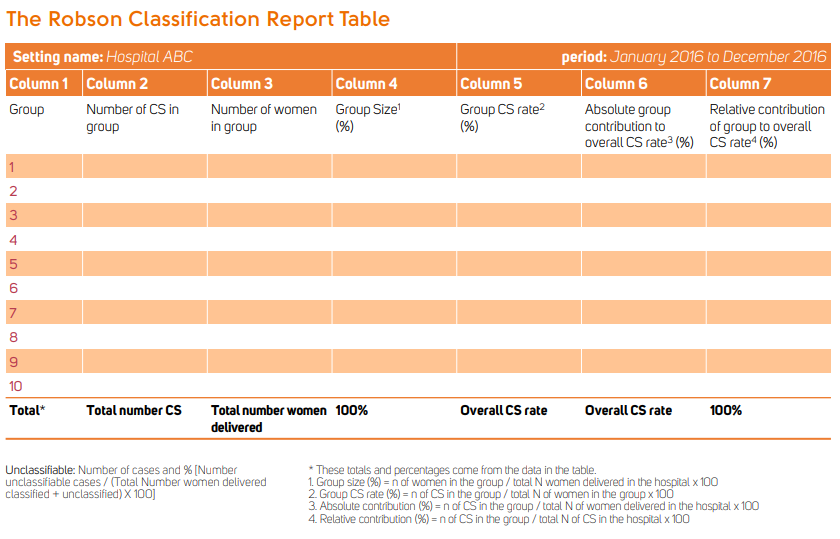
**

## **Interview guide for providers and administrators**

*Interviewer: The next part of the study is about* ***using audit and feedback as a tool for quality improvement****. The purpose of audit and feedback is to encourage individuals and teams to follow professional standards or targets and to monitor changes and outcomes when these are used. During an audit and feedback process, an individual’s or department’s professional practice and/or performance is measured and compared to targets or professional standards. The results of this comparison are fed back to the individual by either a colleague, supervisor or third party, in the form of verbal or written communication. I would like to ask you some questions about what you think about audit and feedback.*

1. Could you tell me about a time where you have been involved in an audit and feedback project?
   1. *If they haven’t been involved in an audit and feedback project, continue to #2 below.*
   2. *If they have been involved in an audit and feedback project:*
      1. What did you find helpful about the audit and feedback process?
      2. What did you find challenging about the audit and feedback process?
      3. What were the main things you learned from the audit and feedback process?
      4. Overall, what was your opinion regarding the audit and feedback process?
2. *Audit and feedback to improve obstetric care may include activities like critical case incident reviews, indications for caesarean section, time from decision to operation for caesarean section, decision-making processes for caesarean section, and appropriate management of complications. This may be done by reviewing individual patient records, labour and delivery logs, and observations of clinical practice.* How would you feel about the idea of a regular audit and feedback process in your health facility to address rising caesarean section rates?
   1. Do you think starting an audit and feedback process may change people’s behaviour in your department? Why or why not?
   2. Do you think starting an audit and feedback process may change health outcomes? Why or why not?
   3. What do you think would be the most important area to start audit and feedback in your health facility?
   4. What areas of health do you think would be most interesting and relevant for audit and feedback? For example, this might include reasons for caesarean section, severe morbidity. Why are these interesting?
   5. What might be some of the benefits of audit and feedback of these aspects related to caesarean section?
   6. What might be some of the challenges of audit and feedback of these aspects related to caesarean section?
   7. Do you think other healthcare providers would be accepting to have an audit and feedback process started in your health facility? Why or why not?
   8. What could be done in your health facility to ensure that audit and feedback is conducted in a supportive way that emphases learning?
   9. What could be done in your health facility to ensure that audit and feedback is not conducted in a punitive way? In other words, to ensure that healthcare providers are not punished for certain behaviours?
   10. What type of character traits would a person need to have to be successful in leading audit and feedback processes in your health facility?
   11. What type of person would be the most appropriate person to conduct audit and provide feedback in your health facility?
       1. *Probe:* Would you prefer that this person were a colleague, supervisor, or someone external? Why?
   12. How can audit and feedback be structured to ensure that any information gathered is “actionable” so that an individual can work to improve their practice?
   13. Approximately how often do you think that audit and feedback processes should occur in your health facility? Please explain.
   14. In your opinion, could incentives that reward changed behaviours help to encourage quality improvement? Explain
       1. What type of incentives would be appropriate in your health facility?

*Interviewer: The next part of the study is about* ***external review of labour and delivery records****, specifically considering caesarean section. This refers to individuals external to a specific health facility reviewing and assessing labour and delivery records, in order to identify areas for improvement around the appropriate use and quality of caesarean section a facility-level. This typically includes reviewing medical records of individual women who received a caesarean section, and may include assessment of indication for caesarean section, case fatality rates, maternal and fetal outcomes, decision-to-incision intervals, administration of prophylactic antibiotics, and use of the partograph or other labour monitoring tool. I would like to ask you some questions about what you think about the external review of labour and delivery records.*

1. Could you tell me about how records are prepared and kept in your health facility?
   1. What is your perception regarding the completeness of labour and delivery records in your health facility?
      1. *Probe:* Do you think that labour and delivery records are complete and accurate for all or most women in your health facility? Why or why not?
   2. *Probe:* Who is responsible for recording in the medical records?
   3. *Probe:* In addition to the individual patient’s record, how else is data collected and recorded on the labour and delivery ward?
      1. *Probe: is there a facility-level logbook? If so, who is responsible for this? What type of data is recorded?*
2. How do you feel about the idea that an external group of people would review labour and delivery records in your health facility?
   1. What are some of the benefits of having an external group of people review these records?
   2. What are some of the challenges of having an external group of people review these records?
   3. Do you think other healthcare providers in your health facility would be accepting to have an external group of people review these records? Why or why not?
      1. *Probe*: Do you think that midwives/nurses and doctors would feel similarly or differently regarding the acceptability of external review of labour and delivery records? Why or why not?
3. Who do you think would be the most appropriate group to review these records?
   1. *Probe:* what type of qualifications would they have?
   2. *Probe:* what type of personal characteristics would they have?
4. *Typically when an external group reviews labour and delivery records, they provide feedback on areas to improve quality. The purpose of the feedback is to learn and improve, not to blame individuals*. What type of format for the feedback would be most appropriate in your health facility?
   1. *Probe*: Would you prefer verbal or written feedback, or both? Explain.
   2. How do you think the feedback could be integrated to improve how care is provided in your facility?
5. Do you have any other comments or feedback about external review of labour and delivery records in your health facility?

*Interviewer: The next part of the study is about the* ***audit and feedback tools for classifying caesarean sections****. These tools may be useful for healthcare providers and administrators to monitor which women are receiving caesarean sections, and also to help to compare caesarean section rates over time or across different health facilities and countries. This may help to design and implement interventions to make sure that an optimal caesarean section rate can occur in a specific health facility. I would like to ask you some questions about what you think of such audit and feedback tools. In order to understand drivers of rising Caesarean section rates, we need to have tools to monitor and compare caesarean section rates in a setting over time. One way to do this is the Robson classification system, which prospectively classifies women admitted for childbirth into one of ten groups.*

1. Have you heard of the Robson classification system before?
   1. *If yes:* Can you tell me what you know about the Robson classification system?
   2. *If no,* Do you know of any other classification systems to classify women giving birth?

*Please show the Robson classification diagram to the participant, and explain: The Robson classification was created to prospectively identify well-defined, clinically relevant groups of women admitted for delivery and to investigate differences in CS rates within these relatively homogeneous groups of women. The Robson Classification is for “all women” who deliver at a specific setting (e.g. a maternity or a region) and not only for the women who deliver by CS. It is a complete perinatal classification. Since this system can be used prospectively and its categories are totally inclusive and mutually exclusive, every woman who is admitted for delivery can be immediately classified, based on a few basic characteristics which are usually routinely collected by obstetric care providers worldwide:*

1. *To implement the Robson classification system, we need the following information: parity, previous caesarean section, onset of labour, gestational age, fetal presentation/lie and number of foetuses from medical records.*
2. Are these data routinely collected in your setting:
   - 1. Parity (nulliparous or multiparous)?
        1. If yes, is there a uniform definition? What is it?
        2. What are some potential challenges regarding incomplete medical records that might complicate data collection about parity?
     2. Previous caesarean section (none, 1 or more)?
        1. If yes, is there a uniform definition? What is it?
        2. What are some potential challenges regarding incomplete medical records that might complicate data collection about previous caesarean section?
     3. Onset of labour (spontaneous, induced, no labour)?
        1. If yes, is there a uniform definition? What is it?
        2. What are some potential challenges regarding incomplete medical records that might complicate data collection about onset of labour?
     4. Augmentation of labour?
        1. If yes, is there a uniform definition? What is it?
        2. What are some potential challenges regarding incomplete medical records that might complicate data collection about augmentation of labour?
     5. Induction of labour?
        1. If yes, is there a uniform definition? What is it?
        2. What are some potential challenges regarding incomplete medical records that might complicate data collection about induction of labour?
     6. Gestational age (preterm <37 weeks, term > 37 weeks)?
        1. If yes, is there a uniform definition? What is it?
        2. What are some potential challenges regarding incomplete medical records that might complicate data collection about gestational age?
     7. Fetal presentation/lie (cephalic, breech, transverse)?
        1. If yes, is there a uniform definition? What is it?
        2. What are some potential challenges regarding incomplete medical records that might complicate data collection about fetal presentation/lie?
     8. Number of foetuses (singleton, multiple)?
        1. If yes, is there a uniform definition? What is it?
        2. What are some potential challenges regarding incomplete medical records that might complicate data collection about number of fetuses?
     9. Do you foresee any other challenges that may complicate data collection?
3. What can be done to ensure that this data is routinely captured for every woman when she is admitted for childbirth in this health facility?
4. *Data from the Robson classification is usually presented in a table and discussed among the healthcare team*. (Interviewer: show participant the Robson classification report table)
   1. How often would you like to see this data presented (for example, weekly, fortnightly, monthly?)
   2. How do you think the Robson classification report should be presented and discussed among the healthcare team?
   3. How do you think the Robson classification report should be displayed (for example, in a report, on a poster, available to the community)?
   4. Would you like to see any health outcomes associated with the different Robson groups?
      1. If yes, what other health outcomes would you like to see?
         1. Would these health outcomes be routinely collected and recorded in your facility?
5. *There are different ways that women can be classified into the 10 groups. For example, someone can manually go through each patient record to retrieve core variables and add a handwritten note to the medical record. Or, information specialists (technology team) can create a software that picks the core variables in an electronic patient record and assigns the Robson group to each record based on a formula.* What do you think would work best in your health facility, and why?
6. Do you have any other comments or feedback about audit and feedback?
