# Supplementary material for: Optimising the use of caesarean section: a generic formative research protocol for implementation preparation
Source: Reprod Health. 2019 Nov 19;16:170. doi: 10.1186/s12978-019-0827-1 (PMC6862737; doi:10.1186/s12978-019-0827-1)
Supplement: Supplementary file 11 — Additional file 11. Qualitative module 7: Mandatory second opinion before conducting a caesarean section. [file 12978_2019_827_MOESM11_ESM.docx]

# **
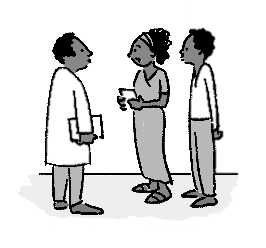
Qualitative module 7: Mandatory second opinion before conducting a caesarean section**

## **Overview of intervention**

### *Background*

Mandatory second opinion refers to the practice of having a physician providing a second opinion on the decision for a non-emergency caesarean section. The physician providing the second opinion should [1]:

1. Have clinical qualifications equal to or higher than the attending physician;
2. Be working in the same hospital;
3. Selected by the obstetrics department; and
4. Have agreed to follow the clinical guidelines, which provide the evidence base to inform the decision to conduct a caesarean section;

and the second opinion should be sought before conducting a non-emergency caesarean section. In this intervention, the physician providing the second opinion assesses each clinical case using pre-specified clinical guidelines and decision-flowcharts for the primary indications for caesarean section [2]. Both physicians discuss the clinical case using the decision-flowcharts to guide their discussion, and the physician providing the second opinion makes the final decision [2].

### *Supporting evidence*

Evidence on the effect of a policy of mandatory second opinion for caesarean section on reducing caesarean section rates are available from one multi-centre cluster randomised trial in Latin America [2] and one interrupted time series study in Taiwan (China) [3]. High certainty evidence shows that implementation of evidence-based clinical guidelines with mandatory second opinion slightly reduces caesarean section rates, but has little or no effect on maternal and perinatal outcomes [1, 2].

Based on this evidence, mandatory second opinion is recommended by WHO, in settings with adequate resources and senior clinicians able to provide mandatory second opinion for caesarean section indication [1].

## **Theory of change**

The proposed theory of change for the impact of mandatory second opinion on caesarean section rates is that the consultation would impact physicians’ attitudes towards indications for caesarean section. The consultation discussion includes the confirmation, re-diagnosis, and options for managing of clinical indications for caesarean section, and thus may influence diagnoses and management plans. Furthermore, mandatory secondary opinion may reduce the likelihood of the use of caesarean section based on provider convenience or scheduling.

## **Participants for qualitative research**

| **Data collection methods and participants** | | |
| --- | --- | --- |
| Population | In-depth interview (IDI) | Focus group discussion (FGD) |
| Women |  | 🗸 |
| Healthcare providers  (midwives, nurses, doctors) | 🗸 |  |
| Healthcare administrators  (matron-in-charge, medical director) | 🗸 |  |

| **Population of women** | | |
| --- | --- | --- |
| Nulliparous | Multiparous with previous CS | Multiparous without previous CS |
|  | 🗸 | 🗸 |

## **Resources and estimated time required to complete this module**

- Trained research assistants
- Audio recorders and notebooks for field notes
- Informed consent forms
- Private room for interviews and group discussions
- Interviews with women: 5-10 minutes
- Interviews with healthcare providers and administrators: 20 minutes

| *Guiding principles*  - The physician providing the second opinion should:   - Have clinical qualifications equal to or higher than the attending physician;   - Assess each clinical case using pre-specified clinical guidelines and decision-flowcharts (evidence-based decision);   - Be working in the same hospital; and   - Selected by the obstetrics department. - The second opinion should always be sought *prior* to conducting the caesarean section. - There should be a transparent process of documenting the decision-making process and final decision. - Obtaining a second opinion should be considered a learning opportunity, and assessment should not lead to punitive measures against the attending physician (in the case of disagreement about clinical indication for caesarean section). |
| --- |

**References**

1. World Health Organization. WHO recommendations on non-clinical interventions to reduce unnecessary caesarean sections. Geneva, Switzerland: World Health Organization; 2018.

2. Althabe F, Belizan JM, Villar J, Alexander S, Bergel E, Ramos S, et al. Mandatory second opinion to reduce rates of unnecessary caesarean sections in Latin America: a cluster randomised controlled trial. Lancet. 2004;363(9425):1934-40.

3. Liang WH, Yuan CC, Hung JH, Yang ML, Yang MJ, Chen YJ, et al. Effect of peer review and trial of labor on lowering cesarean section rates. Journal of the Chinese Medical Association : JCMA. 2004;67(6):281-6.

## **Focus group discussion guide for women**

*Interviewer: The next section of this discussion is about how healthcare is organized in the health facility, and how doctors make decisions about caesarean sections. In some health facilities, doctors must consult with another doctor to discuss why they think the woman should have a caesarean section. The two doctors use some decision-making tools to ensure that a caesarean section is the most appropriate intervention to help the woman and the baby be healthy. This type of intervention is called “mandatory second opinion”. I would like to ask you some questions about what you think about doctors seeking a mandatory second opinion before a woman has a caesarean section.*

1. How do you feel about the idea that a doctor would be required to get a second opinion before deciding that a woman needs a non-emergency caesarean section?
   1. *Probe:* How might women and their babies benefit from doctors getting a second opinion before a caesarean section?
   2. *Probe:* Do you think there are any risks to women and their babies if doctors get a second opinion? Please explain.
2. Could you tell me what you remember about how the decision was made that you would have a caesarean section?
   1. *Probe*: Who was involved in making the decision?
   2. *Probe*: Who had the final say in whether the caesarean section happened?
   3. *Probe*: Do you remember if your doctor said that they would consult with another doctor before deciding for a caesarean section? Please explain.
   4. *Probe:* Do you remember seeing your doctor consult your case with anyone else? Please explain.
      1. *If yes:* How did you feel about your doctor discussing your case with another doctor?
      2. *If no:* How do you think you would have felt if your doctor discussed your case with another doctor?
3. Imagine that the health facility you gave birth in decided to require all doctors to get a second opinion before a non-emergency caesarean section. From your perspective, what might the doctors and administrators need to consider before changing this requirement?
4. Do you have any other comments or feedback about mandatory second opinion for caesarean section?

## **Interview guide for providers and administrators**

*Interviewer: The next section of this interview is about how doctors make decisions about caesarean sections. In some health facilities, doctors must consult with another doctor to discuss if and why they think the woman should have a caesarean section. The doctor who they consult must have clinical qualifications at the same level or higher to the doctor requesting the consult. The two doctors use clinical guidelines and decision-making tools to ensure that a caesarean section is the most appropriate intervention to help the woman and the baby. This type of intervention is called “mandatory second opinion”. I would like to ask you some questions about what you think about doctors seeking a mandatory second opinion before a woman has a caesarean section.*

1. In the health facility where you currently work, what are the clinical indications for caesarean section?
2. Could you tell me about any clinical guidelines to diagnose indications for caesarean section and management for caesarean section used in your health facility? Please explain.
3. Are there any decision-making tools that are used in your health facility to help midwives and doctors to diagnose indications for caesarean section and manage caesarean section? For example, this may include posters, flowcharts or other tools?
   1. How often are these decision-making tools used? (*Probe:* for all women, daily, not used)
4. How do you feel about the idea that a doctor would need to get a second opinion before deciding that a woman needs a non-emergency caesarean section?
   1. *Probe*: How might women and their babies benefit from doctors getting a second opinion before a non-emergency caesarean section?
   2. *Probe:* What challenges would there be to implement a mandatory second opinion before a non-emergency caesarean section?
   3. *Probe*: Do you think there are any risks to women and their babies if doctors get a second opinion? Please explain.
   4. *Probe*: How would you feel if you were now required to obtain a second opinion before a non-emergency caesarean section?
   5. *Probe*: How do you think the other doctors working in your health facility would feel if they were required to obtain a second opinion before a non-emergency caesarean section?
5. *Interviewer: Having a mandatory second opinion for a non-emergency caesarean section requires adequate resources, for example, there needs to be always a second doctor available to provide the consult. This second doctor must have clinical qualifications at the same level or higher to the doctor requiring the consult.* What changes do you think would be required to implement this policy in your facility?
   1. *Probe:* What type of training would need to be provided?
   2. *Probe:* What type of changes to remuneration/salaries need to occur?
   3. *Probe:* What type of changes to supervision would need to occur?
6. How should the health information systems be best adapted to monitor the impact of implementing a mandatory second opinion for caesarean section?
7. Do you have any other comments or feedback about mandatory second opinion for caesarean section in your health facility?
