# Supplementary material for: Optimising the use of caesarean section: a generic formative research protocol for implementation preparation
Source: Reprod Health. 2019 Nov 19;16:170. doi: 10.1186/s12978-019-0827-1 (PMC6862737; doi:10.1186/s12978-019-0827-1)
Supplement: Supplementary file 12 — Additional file 12. Qualitative module 8: In-service training and implementation of clinical practice guidelines. [file 12978_2019_827_MOESM12_ESM.docx]

# **
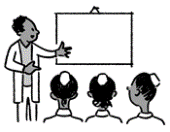
Qualitative module 8: In-service training and implementation of clinical practice guidelines**

## **Overview of intervention**

### *Background*

Across the world, all groups of decision-makers (healthcare providers, patients, administrators, and policy-makers) are facing challenges in using research evidence to inform healthcare decisions. In the context of maternal health, most of the estimated 300,000 maternal deaths annually are preventable (1). This highlights the importance of ensuring the availability of high quality of care, including the implementation of interventions known to be effective (2). Adaptation and implementation of clinical and health systems guidelines is a critical aspect to improve quality of care and accountability. By guidelines, we are referring to recommendations for clinical or public health practice that provides options on what can be done in a specific situation in order to achieve the best outcomes (3). Guideline recommendations can help end-users to select and prioritise across a range of potential interventions (3).

Typically, these guideline recommendations are adapted for use in a specific context, for example, in a specific health facility. The subsequent set of practical statements for implementing these best clinical practices may take the form of a clinician decision-making support tool, referred to as a clinical practice guideline or protocol. For example, this may include a clinical protocol for managing post-partum haemorrhage in health facility A, which would account for local constraints and opportunities including availability of drugs and health worker mix. Clinical practice guidelines can be useful tools to reduce patient harm by improving standardisation and communication.

### *Supporting evidence*

Evidence on the effect of implementation of evidence-based clinical practice guidelines, combined with audit and feedback or mandatory second opinion is available from three cluster-randomised trials, and four interrupted time series studies (4).

High-certainty evidence shows that the implementation of guidelines combined with audit and feedback slightly reduces caesarean section rates (in women with low-risk pregnancies), assisted vaginal delivery, major and minor neonatal morbidity, intrapartum and neonatal deaths, major trauma, and use of invasive mechanical ventilation (4). Similarly, high-certainty evidence shows that the implementation of guidelines combined with mandatory second opinion slightly reduces caesarean section rates (4).

Based on this evidence, implementation of clinical guidelines combined with audit and feedback and/or mandatory second opinion is recommended by WHO (4).

## **Theory of change**

Social influence theory can provide a framework for understanding how relationships and social processes can influence the successful implementation of clinical practice guidelines (5). For example, the perceived values, preferences, attitudes, and opinions of peers and opinion leaders can influence attitudes of individual healthcare providers and their decisions to adopt guidelines into their practice (5). Clinical practice implementation strategies that account for social influence theory may include components such as using opinion leaders, mass media education, and audit and feedback (5). Furthermore, engagement with healthcare providers using consultative and consensus processes is more likely to result in behaviour change, and represents how social influence strategies can be used to build ownership (5).

## **Participants for qualitative research**

| **Data collection methods and participants** | | |
| --- | --- | --- |
| Population | In-depth interview (IDI) | Focus group discussion (FGD) |
| Women |  |  |
| Healthcare providers  (midwives, nurses, doctors) | 🗸 |  |
| Healthcare administrators  (matron-in-charge, medical director) | 🗸 |  |

## **Resources and estimated time required to complete this module**

- Trained research assistants
- Audio recorders and notebooks for field notes
- Informed consent forms
- Private room for interview
- Interviews with healthcare providers and administrators: 15-20 minutes

| *Guiding principles* In order to be effective, clinical and health system guidelines must be appropriately disseminated and implemented. A range of changes will likely be required in the context of specific interventions to encourage the use of guidelines, with the goal of having the guidelines become part of the process of care. Many strategies may be effective in changing providers’ behaviours and impacting health outcomes, such as educational materials, the use of opinion leaders, reminder systems, continuous quality improvement including audit and feedback, local adaptation, and local involvement in implementation and evaluation (6). Adapting and implementing guidelines should account for geographic, demographic, resource, and other factors, and should include clinicians in the decision-making process (6). Successful guideline implementation may include (5):   - Identification of factors affecting implementation from the perspectives of healthcare providers; - Assessment of healthcare provider and health system readiness to change; - Use of multiple methods of implementation as healthcare providers will be in various stages of change and face various factors affecting implementation; - Use of participatory and interactive education for healthcare providers that includes knowledge, attitudes and skill development; - Use of social influence of peers and opinion leaders to facilitate change; and - Environmental and organisational support to maintain change. |
| --- |

**References**

1. World Health Organization. Trends in maternal mortality: 1990 to 2015: estimates by WHO, UNICEF, UNFPA, World Bank Group and the United Nations Population Division.; 2015.

2. Vogel JP, Moore JE, Timmings C, Khan S, Khan DN, Defar A, et al. Barriers, Facilitators and Priorities for Implementation of WHO Maternal and Perinatal Health Guidelines in Four Lower-Income Countries: A GREAT Network Research Activity. PLOS ONE. 2016;11(11):e0160020.

3. World Health Organization. World Health Organization Handbook for Guideline Development. 2nd ed. Geneva, Switzerland: World Health Organization,; 2014.

4. World Health Organization. WHO recommendations on non-clinical interventions to reduce unnecessary caesarean sections. Geneva, Switzerland: World Health Organization; 2018.

5. Moulding NT, Silagy CA, Weller DP. A framework for effective management of change in clinical practice: dissemination and implementation of clinical practice guidelines. Quality in Health Care. 1999;8(3):177.

6. Oxman AD, Thomson MA, Davis DA, Haynes RB. No magic bullets: a systematic review of 102 trials of interventions to improve professional practice. CMAJ: Canadian Medical Association Journal. 1995;153(10):1423-31.

## **Interview guide for providers and administrators**

*Interviewer: The next part of the study is about continuous training and implementation of clinical practice guidelines. This refers to the processes by which guideline recommendations are used to help healthcare providers make informed decisions about how and when to provide care in order to achieve the best health outcomes. I would like to ask you some questions about what you think about guideline implementation.*

1. Are you aware of any clinical practice guidelines related to obstetrics?
   1. *If yes, probe:*
      1. Which clinical practice guidelines are you familiar with? (*Interviewer probe the following:*
         1. Managing normal birth
         2. Postpartum haemorrhage
         3. Sepsis
         4. Indications for caesarean section
         5. Pre-eclampsia and eclampsia
         6. Augmentation of labour
         7. Induction of labour
      2. Are these clinical practice guidelines used in your current health facility? Please explain.
   2. *If no, probe:*
      1. How do healthcare providers in your facility make decisions about how to manage patients?
2. In your health facility, are clinical practice guidelines currently used in obstetrics?
   1. *If yes, probe:*
      1. Which clinical practice guidelines are currently used in obstetrics?
      2. What type of training did you have to understand how to use these clinical practice guidelines?
      3. In your opinion, how valuable are the obstetrics clinical practice guidelines to your practice?
      4. In your opinion, how accessible are these clinical practice guidelines to healthcare providers?
         1. *Probe:* what could be done to improve the accessibility of clinical practice guidelines to other healthcare providers?
      5. Could you describe the process of how obstetrics clinical practice guidelines are prioritised in your health facility?
      6. How are the clinical practice guidelines communicated to other healthcare providers?
      7. How do you use clinical practice guidelines in your practice?
   2. *If no, probe:*
      1. In your opinion, would obstetrics clinical practice guidelines be valuable to your practice? Why or why not?
      2. In your opinion, what could be done to improve the accessibility of clinical practice guidelines to other healthcare providers?
      3. Could you describe the process you would follow to make decisions about how to care for a patient throughout labour?
      4. In your opinion, would obstetrics clinical practice guidelines be a useful addition to your practice? Why or why not?
3. *Imagine that your health facility will start a process of adapting and implementing obstetrics clinical* practice guidelines*.* Who would need to support this initiative in order for it to be successful?
   1. *Probe:* Why would this person/these people need to support the initiative?
   2. *Probe:* How would this person/these people best support the initiative?
   3. What type of training would be helpful to ensure that all staff understand the clinical practice guidelines?
      1. What type of topics would you like to have covered during the training?
         1. Would you be interested to learn about how clinical practice guidelines were developed? Why or why not?
         2. Would you be interested to learn about the evidence behind the recommendations in clinical practice guidelines, such as the systematic reviews or clinical trials?
      2. How long should the training last for?
      3. How often should the training be repeated?
   4. What resources would be needed in order to successful implement obstetrics clinical protocols?
   5. In your opinion, what are some barriers to successful implementation of obstetrics clinical practice guidelines?
   6. In your opinion, what are some facilitators to successful implementation of obstetrics clinical practice guidelines?
   7. What could be done in your facility to ensure that the implementation of obstetrics clinical practice guidelines is a learning process?
   8. *Usually when clinical practice guidelines* *are implemented in health facilities, there are activities to evaluate if the guidelines are being implemented correctly and consistently*. What type of evaluation activities would be helpful to assess if obstetrics clinical practice guidelines were being implemented correctly and consistently?
      1. What format would be appropriate to feedback the evaluations to healthcare providers?
      2. If meetings were held to feedback on the progress of obstetric clinical practice guidelines implementation, what would you like to hear discussed?
         1. Who would attend these meetings and why?
         2. How often would these meetings be held?
4. Do you have any other comments or feedback about the obstetrics clinical practice guidelines and implementing the guidelines?
