# Supplementary material for: Optimising the use of caesarean section: a generic formative research protocol for implementation preparation
Source: Reprod Health. 2019 Nov 19;16:170. doi: 10.1186/s12978-019-0827-1 (PMC6862737; doi:10.1186/s12978-019-0827-1)
Supplement: Supplementary file 13 — Additional file 13. Qualitative module 9: Equalising physician pay for vaginal and caesarean birth. [file 12978_2019_827_MOESM13_ESM.docx]

# **
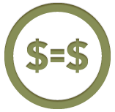
Qualitative module 9: Equalising physician pay for vaginal and caesarean birth**

## **Overview of intervention**

### *Background*

In many settings, providers are paid a higher rate for conducting caesarean sections, compared to vaginal births, partially due to the perceived additional resources needed to conduct an operative birth. It has been hypothesised that this favourable reimbursement scheme has contributed to the rising caesarean section rates. In response, some governments and insurers have enacted policies to equalise physician payment for caesarean and vaginal births. However, these policy interventions have often been met with mixed results (1). A key limitation in previous research is the inability to distinguish between elective and clinically indicated caesarean sections, which compromises the ability to detect the impact of equalised pay (2).

### *Supporting evidence*

Two interrupted time series studies have explored financial strategies to equalise physician fees for vaginal and caesarean births (including vaginal birth after caesarean) in Taiwan and the United States of America (3, 4). The certainty of evidence for the impact of this intervention on caesarean section rates is uncertain. However, financial incentives remain a major determinant of caesarean births in all settings (5).

Based on this evidence, financial strategies to equalise physician fees for vaginal and caesarean birth is recommended by WHO, in the context of rigorous research on: (1) the impact on caesarean section rates; (2) acceptability to key stakeholders; and (3) feasibility of implementation.

## **Theory of change**

Economic theories of provider behaviour posit that when healthcare providers choose the type of services provided to their patients, cost-based reimbursement systems may result in too many services being provided (6). In many settings, physicians are the dominant decision-makers for the level and type of services provided, including caesarean section, thus suggesting that supply-side incentives (such as equalising physician pay for vaginal and caesarean birth) may be more effective than demand-side incentives (e.g. that target women and their families to reduce demand for caesarean section) (6). Economic theories of provider behaviour also suggest that healthcare providers are not always acting as rational agents, due to the influence of the economic power of health facilities and perceived benefit at an individual-level (6). Equalising pay for vaginal and caesarean birth may therefore reduce financial incentives to the healthcare provider, and may contribute to a reduction in unnecessary caesarean sections.

## **Participants for qualitative research**

| **Data collection methods and participants** | | |
| --- | --- | --- |
| Population | In-depth interview (IDI) | Focus group discussion (FGD) |
| Women |  |  |
| Healthcare providers  (midwives, nurses, doctors) | 🗸 |  |
| Healthcare administrators  (matron-in-charge, medical director) | 🗸 |  |

## **Resources and estimated time required to complete this module**

- Trained research assistants
- Audio recorders and notebooks for field notes
- Informed consent forms
- Private room for interview
- Interviews with healthcare providers and administrators: 15-20 minutes

| *Guiding principles*  - To minimise conflict, it may be helpful to ensure that provider perspectives on changing financial incentives are taken into account prior to any change in structure. - Vaginal birth may be more time intensive compared to caesarean birth. Therefore, even if physician pay for vaginal and caesarean birth are equalised, caesarean birth may still be chosen without medical indication. - Facility structure, materials and human resources may need to be adapted to accommodate a potential increase in the number of vaginal births. |
| --- |

**References**

1. Chen I, Opiyo N, Tavender E, Mortazhejri S, Rader T PJ, Yogasingam S, Taljaard M, Agarwal S, Laopaiboon M, Wasiak J, Khunpradit S, Lumbiganon P, GR, Betran AP. Non-clinical interventions for reducing unnecessary caesarean section. Cochrane Database of Systematic Reviews. Sep 28;9:CD005528. doi: 10.1002/14651858.CD005528.pub3.

2. Chen C-S, Liu T-C, Chen B, Lin C-L. The failure of financial incentive? The seemingly inexorable rise of cesarean section. Social Science & Medicine. 2014;101:47-51.

3. Keeler EB, Fok T. Equalizing physician fees had little effect on cesarean rates. Medical care research and review : MCRR. 1996;53(4):465-71.

4. Lo JC. Financial incentives do not always work: an example of cesarean sections in Taiwan. Health Policy. 2008;88(1):121-9.

5. World Health Organization. WHO recommendations on non-clinical interventions to reduce unnecessary caesarean sections. Geneva, Switzerland: World Health Organization; 2018.

6. Ellis RP, McGuire TG. Provider behavior under prospective reimbursement: Cost sharing and supply. Journal of Health Economics. 1986;5(2):129-51.

**Interview guide for providers and administrators**

*Interviewer: The next part of the study is about the financial costs associated with caesarean section and vaginal birth. I would like to ask you some questions about what you think about the financial costs associated with different modes of childbirth.*

1. In your opinion, are there financial incentives for healthcare providers or health facilities to provide caesarean sections?
2. *Probe:* Why or why not?
3. *Probe:* If so, what are the financial incentives?
4. *Probe:* If not, why do you think there are no financial incentives?
5. From your perspective, is a caesarean section more or less work for a healthcare provider, compared to a vaginal birth? Please explain.
6. *In some places, there are policies to equalise the cost of caesarean sections and vaginal births. Typically, this means increasing the cost of vaginal births to the current rate of caesarean section, rather than reducing the cost of caesarean section to the current rate of vaginal births.* What do you think about this type of policy?
7. How would you feel if this type of policy was implemented in your health facility or your country?
8. Do you think that healthcare providers in your facility or your country would support this type of policy? Why or why not?
9. What type of challenges do you think there would be if this type of policy were implemented in your health facility or your country?
10. What type of benefits do you think there would be if this type of policy were implemented in your health facility or your country?
11. What impact would this type of policy have on healthcare providers?
12. What impact would this type of policy have on the health facility?
13. What impact would this type of policy have on women?
14. What resources would your health facility need to implement this type of policy?
15. Do you have any other comments or feedback about equalising pay for vaginal and caesarean birth?
