# Supplementary material for: Optimising the use of caesarean section: a generic formative research protocol for implementation preparation
Source: Reprod Health. 2019 Nov 19;16:170. doi: 10.1186/s12978-019-0827-1 (PMC6862737; doi:10.1186/s12978-019-0827-1)
Supplement: Supplementary file 14 — Additional file 14. Qualitative module 10: Opinion leader education. [file 12978_2019_827_MOESM14_ESM.docx]

# **
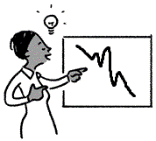
Qualitative module 10: Opinion leader education**

## **Overview of intervention**

### *Background*

Opinion leader education is an intervention that aims to change the culture and norms of healthcare provider peer groups using an influential individual to drive change. In the context of caesarean section, opinion leaders have been trained on emerging research and clinical recommendations related to optimising the use of caesarean section, as well as educational tools for behaviour change and quality improvement in a healthcare setting [1, 2]. For example, this might include how to implement trial of labour for women who have a previous caesarean section, in order to increase vaginal birth after caesarean section. Opinion leaders then lead the knowledge translation from published clinical recommendations to appropriate implementation in a specific healthcare setting. Opinion leaders are typically identified by their peers, based on individuals who best meet a set of prespecified criteria [1], such as those who are knowledgeable, good communicators and humanistic [3].

### *Supporting evidence*

High-certainty evidence shows that the use of local opinion leader education as a method to implement guidelines reduces rates of elective caesarean section, and increases the proportion of women who have vaginal births [1, 2]. The available evidence is from a multifaceted intervention comprising implementation of evidence-based clinical practice guidelines (onsite training, facilitation of implementation by a local opinion leader, supportive supervision) and audit and feedback.

Based on this evidence, the use of opinion leaders for the facilitation of implementation of interventions such as on-site training in evidence-based clinical practice is recommended by WHO together with supportive supervision.

## **Theory of change**

Evidence has demonstrated that the application of research findings and guideline implementation can be slow, and that traditional dissemination approaches (e.g. publication of guidelines or journal articles) is unlikely to lead to changes in practice [3]. More recently, there has been greater awareness of other environmental factors that influence behaviours and practices, which need to be accounted for during implementation [3]. Research has demonstrated that healthcare providers share common beliefs and group norms with their peer groups, and that these norms can directly influence individual behaviours [3]. The concept of opinion leadership proposes that influential healthcare providers take advantage of the structure of these influential group networks to change norms and drive quality improvement in a healthcare setting.

## **Participants for qualitative research**

| **Data collection methods and participants** | | |
| --- | --- | --- |
| Population | In-depth interview (IDI) | Focus group discussion (FGD) |
| Women |  |  |
| Healthcare providers  (midwives, nurses, doctors) | 🗸 |  |
| Healthcare administrators  (matron-in-charge, medical director) | 🗸 |  |

## **Resources and estimated time required to complete this module**

- Trained research assistants
- Audio recorders and notebooks for field notes
- Informed consent forms
- Private room for interview
- Interviews with healthcare providers and administrators: 5-10 minutes

| *Guiding principles*  - The opinion leader(s) should be chosen by their peers in a transparent method. For example, this may include by a survey of other healthcare providers. - Adequate resources should be provided to opinion leader(s) to be able to implement change in their context. |
| --- |

**References**

1. Lomas J, Enkin M, Anderson GM, Hannah WJ, Vayda E, Singer J. Opinion leaders vs audit and feedback to implement practice guidelines: Delivery after previous cesarean section. JAMA. 1991;265(17):2202-7.

2. World Health Organization. WHO recommendations on non-clinical interventions to reduce unnecessary caesarean sections. Geneva, Switzerland: World Health Organization; 2018.

3. Grimshaw JM, Eccles MP, Greener J, Maclennan G, Ibbotson T, Kahan JP, et al. Is the involvement of opinion leaders in the implementation of research findings a feasible strategy? Implementation Science. 2006;1(1):3.

## **Interview guide for providers and administrators**

*Interviewer: The next part of the study is about using opinion leaders in a specific health facility to act as champions for change.* *Opinion leaders are influential individuals who are nominated by their peers to change the culture and norms of healthcare provider peer groups. For example, these individuals may be responsible for adapting clinical guidelines to a specific health facility context, and identifying measures to ensure quality improvement. I would like to ask you some questions about what you think about the use of opinion leaders in your health facility.*

1. What do you think are the characteristics of a good opinion leader?
2. What do you think about the idea of using opinion leaders to adapt clinical guidelines to your health facility?
3. What type of healthcare provider would be most appropriate to act as an opinion leader for caesarean section? *(probe: nurse/midwife/doctor, what level of training)*
4. How do you think an opinion leader would be received by other healthcare providers in your health facility?
5. What challenges do you think an opinion leader would face if they tried to adapt and implement clinical guidelines in your health facility?
6. What type of *training* would an opinion leader need to succeed?
   1. *Probe:* training on clinical guideline adaptation?
   2. *Probe:* training on behaviour change?
   3. *Probe:* training in human relations?
   4. *Probe:* training in quality improvement?
7. What resources would an opinion leader need to succeed? *(Probe: Human resources? Financial resources?)*
8. Do you have any other comments or feedback about using opinion leaders in your health facility to act as champions for change of caesarean section practices?
