# Supplementary material for: Optimising the use of caesarean section: a generic formative research protocol for implementation preparation
Source: Reprod Health. 2019 Nov 19;16:170. doi: 10.1186/s12978-019-0827-1 (PMC6862737; doi:10.1186/s12978-019-0827-1)
Supplement: Supplementary file 15 — Additional file 15. Qualitative module 11: Setting a goal for caesarean section rate at a facility-level. [file 12978_2019_827_MOESM15_ESM.docx]

# **
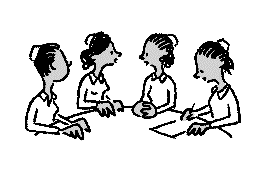
Qualitative module 11: Setting a goal for caesarean section rate at a facility-level**

## **Overview of intervention**

### *Background*

Setting goals for caesarean section rates at a hospital level may help to reduce caesarean section rates for low-risk women. Hospital-specific goals may be a useful way to ensure the flexibility of an “ideal” caesarean section rate, as the target can be adjusted based on the level of hospital (e.g. tertiary vs primary), and the characteristics of the population (e.g. higher or lower risk women). Goals may be agreed upon by hospital leaders, in consultation with their staff and external stakeholders such as health managers or community groups. Goals may target specific Robson groups as opposed to overall caesarean section rate [1, 2]. Over time, goals and targets can progressively be reassessed and modified to reflect the dynamics of quality improvement initiatives and progress.

### *Supporting evidence*

There is limited evidence to support or refute the hypothesis that goal setting at a hospital level can reduce caesarean section rates, thus studies evaluating the effects of this intervention are needed [3]. Ideally, these studies will include goal setting at the hospital level as one component of a multifaceted strategy [3]. One interrupted time series study implemented a multifaceted strategy including evidence-based guidelines, 24 hour coverage by an attending physician and a hospital-level caesarean section target [4]. This study found that the overall caesarean section rate decreased over a six year period from 23.2% to 16.0%, primary caesarean section rates decreased from 13.8% to 9.7%, and repeat caesarean section rates decreased from 9.4% to 6.3% [4].

Based on the limited evidence, WHO has called for more research to explore the impact of goal setting for caesarean section rates at a hospital level [3].

## **Theory of change**

The process by which goals are identified and set inherently implies an expression of dissatisfaction with current conditions and desire to change or improve for the future [5]. Prior to setting goals, it is important to conduct a problem analysis to understand the issue in a given context, and select an intervention or interventions that will address the issue; this may be referred to as a problem-based goal-setting approach [6]. A key underpinning of goal-setting theories is the power of goals to motivate provider behaviour change in order to achieve the desired change or outcome [5]. Goals may motivate better individual task performance by inspiring behaviour change around the goal-relevant behaviours [5]. In the context of reducing caesarean section rates, goal setting at the hospital level may inspire, motivate, and stimulate progress [7] of individual providers and teams to focus on improving their performance and capacity to achieve the desired outcome.

**Participants for qualitative research**

| **Data collection methods and participants** | | |
| --- | --- | --- |
| Population | In-depth interview (IDI) | Focus group discussion (FGD) |
| Women |  |  |
| Healthcare providers  (midwives, nurses, doctors) | 🗸 |  |
| Healthcare administrators  (matron-in-charge, medical director) | 🗸 |  |

## **Resources and estimated time required to complete this module**

- Trained research assistants
- Audio recorders and notebooks for field notes
- Printed handouts of the Robson classification groups and table for presenting Robson data
- Informed consent forms
- Private room for interview
- Interviews with healthcare providers and administrators: 5-10 minutes

| *Guiding principles* Successful goal setting and provider behaviour change may be more likely to be successful when [4, 8]:   - Healthcare providers agree that rising caesarean section rates are a problem; - Goals are contextualised and are meaningful for providers; - Individual healthcare providers and departments adopt evidence-based guidelines in their daily practice; - Opinion leaders advocate for change and are respected by their colleagues; - Consensus, collaboration, and participatory decision-making are prioritised; - Audit and feedback are integrated in a consistent manner; and - Healthcare providers are ready and willing to change behaviours. |
| --- |

**References**

1. World Health Organization. WHO Statement on Caesarean Section Rates. Geneva, Switzerland; 2015.

2. World Health Organization. Robson Classification: Implementation Manual. Geneva, Switzerland; 2017.

3. World Health Organization. WHO recommendations on non-clinical interventions to reduce unnecessary caesarean sections. Geneva, Switzerland: World Health Organization; 2018.

4. Poma PA. Effect of Departmental Policies on Cesarean Delivery Rates: A Community Hospital Experience. Obstetrics & Gynecology. 1998;91(6):1013-8.

5. Locke EA, Latham GP. New Directions in Goal-Setting Theory. Current Directions in Psychological Science. 2006;15(5):265-8.

6. Locke EA, Latham GP. Building a practically useful theory of goal setting and task motivation. A 35-year odyssey. The American psychologist. 2002;57(9):705-17.

7. Nanji KC, Ferris TG, Torchiana DF, Meyer GS. Overarching goals: a strategy for improving healthcare quality and safety? BMJ Quality &amp;amp; Safety. 2013;22(3):187.

8. Ivers N, Jamtvedt G, Flottorp S, Young JM, Odgaard-Jensen J, French SD, et al. Audit and feedback: effects on professional practice and healthcare outcomes. The Cochrane database of systematic reviews. 2012;6:CD000259.

**The 10 groups of the Robson classification** [2]

**
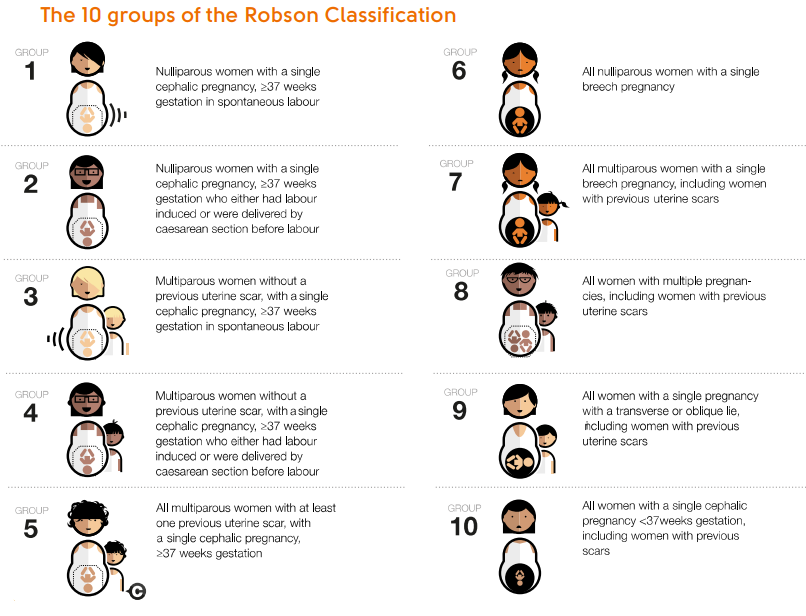
**

## **Interview guide for providers and administrators**

*Interviewer: The next part of the study is about setting goals for caesarean section rates at a hospital level. Setting goals or targets may help to reduce caesarean section rates. For example, a hospital-level goal may be to reduce caesarean section rates from 45% to 30% from June to December 2019. I would like to ask you some questions about what you think about setting goals for caesarean section rates at a hospital level.*

1. How would you feel about setting a hospital-specific caesarean section rate?
   1. What are some of the benefits of setting a hospital-specific caesarean section rate?
   2. What are some of the challenges of setting a hospital-specific caesarean section rate?
   3. Would you be supportive of having a hospital-specific caesarean section rate in your current hospital? Why or why not?
   4. Which do you think would be more useful to you and your colleagues: (1) to have an overall target caesarean section rate for ALL women; or (2) to have a target caesarean section rate for women with different characteristics, such as by each Robson classification group? Please explain.
2. If your hospital were setting a hospital-specific caesarean section rate, who do you think would be the most appropriate people to develop the target?
   1. Do you think that other healthcare providers should be consulted to develop the rate? Why or why not?
   2. Should any other external stakeholders be involved in developing the target?
      1. *Probe:* insurance companies? Health system managers? Community groups?
3. If your hospital were setting a hospital-specific caesarean section rate, how often would you like to see progress towards achieving this rate evaluated?
   1. *Probe:* for example, this might be monthly, once every 3 or 6 months, or once a year?
   2. *Probe:* Why do you think this is the most appropriate feedback interval?
   3. How would you like to see the progress towards achieving this rate reported?
      1. *Probe*: through reports? Wall charts? Presentations?
   4. What do you think you would need to achieve the targets?
   5. Do you think that there should be rewards or incentives for reaching the goal?
      1. *Probe:* Why or why not?
      2. If yes, what type of reward or incentive would be appropriate?
      3. If no, what would you think could help motivate people to try to reach the target?
4. Which stakeholders do you think should be updated on progress towards achieving the target caesarean section rate?
   1. *Probe:* insurance companies? Health system managers? Community groups?
5. Do you have any other comments or feedback about setting a hospital-specific caesarean section rate?
