# Supplementary material for: Optimising the use of caesarean section: a generic formative research protocol for implementation preparation
Source: Reprod Health. 2019 Nov 19;16:170. doi: 10.1186/s12978-019-0827-1 (PMC6862737; doi:10.1186/s12978-019-0827-1)
Supplement: Supplementary file 16 — Additional file 16. Qualitative module 12: Policies limiting legal liability and malpractice lawsuits. [file 12978_2019_827_MOESM16_ESM.docx]

# **
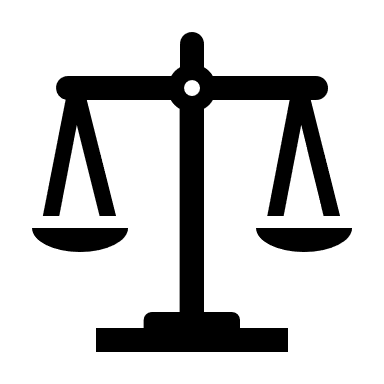
Qualitative module 12: Policies limiting legal liability and malpractice lawsuits**

## **Overview of intervention**

### *Background*

Fears of legal liability and medical malpractice litigation may be a factor influencing rising caesarean section rates. These fears may influence providers to favour caesarean section over vaginal birth, as well as discourage vaginal birth after caesarean section (VBAC) [1]. In some settings, obstetricians pay more for liability insurance coverage and are sued more frequently, compared to physicians in other specialties [1]. Caesarean section may be seen as a protective mechanism to avoid litigation, rather than an alternative mode of birth when vaginal birth is not possible [1]. Evidence of the association between legal liability and caesarean section rates is mixed, as some studies have demonstrated a positive association [2-5], and others have demonstrated no relationship [6-8]. Mixed evidence of the association between legal liability and caesarean section may not be surprising given the variance in legal structures and practices across contexts.

### *Supporting evidence*

There is limited evidence to support or refute the hypothesis that policies limiting legal liability and malpractice lawsuits can reduce caesarean section rates, thus studies evaluating the effects of these policies are needed [9]. Ideally, these studies will include policies at the hospital- or health system-level as one component of a multifaceted strategy [9]. A modelling study in the United States of America explored the impact of state-level liability environments on the rates of caesarean section and found that malpractice premiums were positively associated with rates of caesarean section, and negatively associated with VBAC [1]. Furthermore, two types of legislative reform (caps on noneconomic damages and pretrial screening panels) were associated with lower caesarean rates and higher VBAC rates [1].

Based on the limited evidence, WHO has called for more research to explore the impact of policies limiting legal liability and malpractice lawsuits on caesarean section rates [9].

## **Theory of change**

Some research has demonstrated that obstetricians may view caesarean sections as a way to limit their exposure to malpractice lawsuits [1]. If policies were enacted to limit an obstetrician’s legal liability in the case of obstetric complications, then obstetricians may be less fearful of legal liability, and consequently reduce their use of caesarean section as a defensive measure against litigation.

## **Participants for qualitative research**

| **Data collection methods and participants** | | |
| --- | --- | --- |
| Population | In-depth interview (IDI) | Focus group discussion (FGD) |
| Women |  |  |
| Healthcare providers  (midwives, nurses, doctors) | 🗸 |  |
| Healthcare administrators  (matron-in-charge, medical director) | 🗸 |  |

## **Resources and estimated time required to complete this module**

- Trained research assistants
- Audio recorders and notebooks for field notes
- Informed consent forms
- Private room for interview
- Interviews with healthcare providers and administrators: 10 minutes

| *Guiding principles*  - Over-medicalization of childbirth does not result in better outcomes; a caesarean section can effectively prevent maternal mortality and morbidity only when it is medically justified. However, there is no evidence showing the benefits of a caesarean delivery for women or infants who do not need the procedure. - Providers may need support and a reliable legal framework so that they are not legally vulnerable, even if they deliver the best evidence-based care. - Legal frameworks should not protect against negligence; legal frameworks should clearly differentiate between adverse outcomes following implementation of best practices and the failure to provide appropriate care. |
| --- |

**References**

1. Yang YT, Mello MM, Subramanian SV, Studdert DM. Relationship Between Malpractice Litigation Pressure and Rates of Cesarean Section and Vaginal Birth After Cesarean Section. Medical care. 2009;47(2):234-42.

2. Murthy K, Grobman WA, Lee TA, Holl JL. Association between rising professional liability insurance premiums and primary cesarean delivery rates. Obstetrics and gynecology. 2007;110(6):1264-9.

3. Dubay L, Kaestner R, Waidmann T. The impact of malpractice fears on cesarean section rates. J Health Econ. 1999;18(4):491-522.

4. Localio AR, Lawthers AG, Bengtson JM, Hebert LE, Weaver SL, Brennan TA, et al. Relationship between malpractice claims and cesarean delivery. JAMA. 1993;269(3):366-73.

5. Tussing AD, Wojtowycz MA. The cesarean decision in New York State, 1986. Economic and noneconomic aspects. Med Care. 1992;30(6):529-40.

6. Baldwin LM, Hart LG, Lloyd M, Fordyce M, Rosenblatt RA. Defensive medicine and obstetrics. JAMA. 1995;274(20):1606-10.

7. Sloan FA, Entman SS, Reilly BA, Glass CA, Hickson GB, Zhang HH. Tort liability and obstetricians' care levels. International Review of Law and Economics. 1997;17(2):245-60.

8. Baicker K, Chandra A. The Effect of Malpractice Liability on the Delivery of Health Care. National Bureau of Economic Research Working Paper Series. 2004;No. 10709.

9. World Health Organization. WHO recommendations on non-clinical interventions to reduce unnecessary caesarean sections. Geneva, Switzerland: World Health Organization; 2018.

## **Interview guide for providers and administrators**

*Interviewer: The next part of the study is about legal liability for complications during childbirth. In many settings, doctors are held responsible for these complications, and may be sued by women and their families if something went wrong. I would like to ask you some questions about what you think about legal liability for complications during childbirth.*

1. Do you feel afraid of malpractice lawsuits in your current work? Please explain.
2. What strategies do you employ to minimise the risk of a malpractice lawsuit?
   1. Do you think these strategies are reasonable?
3. How do you feel about the current environment around malpractice lawsuits and legal liability for doctors?
   1. Do you feel that the health system or your health facility would support you in a legal case?
   2. How do you think that the legal environment may influence your own, or your colleagues’, medical practice?
4. Do you think that changes should be made to regarding legal liability and malpractice for doctors? Please explain.
   1. *Probe:* What type of changes do you think should be made?
   2. *Probe:* What impact do you think these changes may have?
5. Is trial of labour for women with a previous caesarean section, or vaginal birth after caesarean section, something that is practiced in your health facility? Please explain.
   1. What are some of the challenges associated with offering a trial of labour for women with a previous caesarean section?
   2. What are some of the benefits associated with offering a trial of labour for women with a previous caesarean section?
   3. How comfortable do you feel offering a trial of labour for women with a previous caesarean section? Please explain.
   4. If something went wrong during a trial of labour for women with a previous caesarean section, to what extent should the doctor be held responsible?
